# Pfizer Versus Moderna COVID-19 Vaccine Effectiveness Against Mortality: Evidence from Linked Mortality and Vaccination Records in Indiana

**DOI:** 10.3390/vaccines14080717

**Published:** 2026-08-20

**Authors:** Sadia Farzana, Francesco Maria Rossi, Qian Eric Luo, Jeff Whittle, Kevin McGurk, Benjamin W. Weston, Andy Ye Yuan, Ali Moghtaderi, Vladimir Atanasov, Bernard S. Black

**Affiliations:** 1 Pritzker School of Law, Northwestern University, Chicago, IL 60611, USA; francesco.rossi@law.northwestern.edu (F.M.R.); bblack@kellogg.northwestern.edu (B.S.B.); 2 Department of Health Policy and Management, Milken Institute School of Public Health, George Washington University, Washington, DC 20053, USA; qluo@gwu.edu (Q.E.L.); moghtaderi@email.gwu.edu (A.M.); 3 Department of Medicine, Medical College of Wisconsin, Wauwatosa, WI 53226, USA; jwhittle@mcw.edu (J.W.); kjmcgurk@mcw.edu (K.M.); beweston@mcw.edu (B.W.W.); 4 Levin College of Law, University of Florida, Gainesville, FL 32611, USA; yuan@law.ufl.edu; 5 Raymond A. Mason School of Business, William & Mary, Williamsburg, VA 23185, USA; vladimir.atanasov@mason.wm.edu

**Keywords:** COVID-19, COVID-19 mortality, COVID excess mortality percentage, COVID-19 vaccine remaining mortality risk, healthy-vaccinee bias

## Abstract

**Objective**: It is commonly believed in the public health community that the mRNA vaccines from Pfizer and Moderna were broadly similar in preventing COVID-19 mortality, with perhaps a small advantage for Moderna. We revisit this question, using a research design that controls for healthy-vaccinee bias. **Methods**: We studied deaths among persons aged 15 years or greater from January 2021 through December 2022 in Indiana, linked to vaccination records, using an outcome measure, namely COVID-19 Excess Mortality Percentage (CEMP), which uses non-COVID natural mortality to control for HVB. **Results**: We find both large healthy-vaccinee bias for both vaccines, and larger bias for Pfizervaccinees: Pfizer recipients were healthier and thus would have faced lower COVID-19 mortality without vaccination. For ages 60+, we find almost 50% higher COVID-19 remaining mortality risk (RMR) for Pfizer vaccinees receiving primary vaccination over a “core period” from 2Q2021 through 1Q2022: the average Pfizer/Moderna RMR ratio was 1.49. For ages 15–59, this ratio was 1.25, but was not statistically significant. For booster dose for ages 60+, the average Pfizer/Moderna RMR ratio was 1.34. For 2Q2022 through 4Q2022, after the major Omicron wave, we find much lower vaccine effectiveness and no significant Pfizer–Moderna difference. **Conclusions**: During the period with both good vaccine availability and high COVID-19 mortality (2Q-2021–1Q2022), the Moderna vaccine was associated with lower COVID-19 mortality, relative to the Pfizer vaccine. A possible explanation is the higher Moderna dose (100 μg vs. 30 for Pfizer). After that period, a high percentage of the population had natural immunity from prior COVID-19 infection; we find only small mortality gains from vaccination, with no significant Pfizer–Moderna difference. **Policy Implications**: Controlling for healthy-vaccinee bias, Moderna was associated with lower COVID-19 mortality, relative to Pfizer. This difference was not known in real-time due to the failure of the available studies to address healthy-vaccinee bias. Controlling for such bias is vital when making vaccination policy.

## 1. Introduction

The Pfizer–BioNTech (BNT162b2) and Moderna (mRNA1273) COVID-19 vaccines provided important protection against COVID-19 infection, severe disease, and death during the COVID-19 pandemic (for succinctness, we cite principally systematic reviews) [1,2]. These vaccines are commonly viewed in the public health community as providing similar protection against death with perhaps a modest advantage for Moderna, which wanes more slowly. For example, a study of VA patients by Dickerman finds a Moderna advantage in preventing hospitalization but a small, statistically insignificant Moderna advantage against mortality, with a wide confidence interval due to few events [3]; other VA studies found mixed results [4,5]. However, most Pfizer-vs-Moderna comparisons do not carefully address selection effects. In prior work using data from Milwaukee County, Wisconsin, we found evidence that (i) Pfizer vaccinees were typically healthier than Moderna vaccinees, with health proxied by group mortality rates from all non-COVID natural causes; and (ii) after controlling for these differences in background health, primary vaccination with Moderna was associated with lower risk of COVID-19 death, compared to Pfizer [6]. For booster recipients, the Pfizer–Moderna gap narrowed and became statistically insignificant. We found no significant Pfizer-vs-Moderna difference in mortality risk for younger persons.

We revisit here the question of whether Moderna was superior to Pfizer in preventing COVID-19 death, and if so, for which age groups; using a much larger sample than our prior study [6]—the entire state of Indiana (roughly 7.5× the population of Milwaukee County). We also have a much larger sample than Dickerman et al. [3], a population-representative sample, unlike their study, which is limited to VA patients (mostly older men), and a longer time period, including the Delta and Omicron waves (their sample period runs only through June 2021). We study the period through the end of 2022; the Milwaukee study ended with 2Q2022. We study primary vaccination (one or two doses) and booster vaccination (3+ doses).

COVID-19 vaccinees are generally healthier than unvaccinated persons, a phenomenon known as healthy-vaccinee bias (HVB) [6,7,8,9,10,11,12]. It also turns out that Pfizer vaccinees are generally healthier than Moderna vaccinees. To address these selection effects, we use the COVID-19 Excess Mortality Percentage (CEMP) as our principal outcome. CEMP is defined as COVID-19 deaths divided by non-COVID natural deaths for groups defined by age and vaccination status, converted to a percentage. The idea behind this measure is that the CEMP denominator (non-COVID-19 natural deaths) acts as an imperfect proxy for population health, that can control for health differences between two groups, including vaccinated versus unvaccinated or, in this study, Pfizer-versus-Moderna vaccinees. We have used the CEMP-based approach in our prior work [6,7,8,13,14,15], and have confirmed that non-COVID-19 natural mortality is a strong predictor of COVID-19 mortality and provide additional evidence here.

We provide evidence on HVB and on Pfizer-versus-Moderna selection bias. We then use CEMP as an outcome and measure the COVID-19 relative mortality risk (RMR) for Pfizer and Moderna vaccinees versus the unvaccinated, and versus each other, for Indiana over January 2021 through December 2022. However, we focus attention primarily on a “core” period from 2Q2021 to 1Q2022. This is the period during which vaccines were widely available, but COVID-19 mortality remained high, including the Alpha, Delta, and early Omicron COVID-19 variants.

There are many studies of COVID-19 vaccine effectiveness, including effectiveness against mortality (VE = 1 − RMR) [1,2]. However, most studies have limited controls for selection effects. Some control for selected individual characteristics (e.g., age, sex) [16,17,18,19], or study short time periods [16,20,21,22]. Most U.S.-based studies lack population-level data or have only limited health information [4,18,23,24,25]. Many studies use test-negative designs [4,20,24], which are known to be subject to selection bias [25,26,27]. The tendency for healthier people to become vaccinated is called healthy-vaccinee bias (HVB) [9,10,11,28]. If HVB affects COVID-19 vaccination decisions, lower vaccinee mortality rates will partly reflect their better health and thus lower the inherent risk of COVID-19 mortality.

## 2. Data, Methods, and Sources of Potential Bias

### 2.1. Data and Basic Assumptions

We obtained mortality and vaccination records from the Indiana Health Insurance Exchange (IHIE), for all Indiana residents aged 15+, with age measured at the time of the first vaccine dose. These records are linked at the individual level. Of these persons, 3,840,232 (70.3%) received at least one COVID-19 vaccine dose, including 1,334,601 persons aged 60+. The mortality records include age at death, sex, manner of death, cause of death and conditions contributing to death. We lack data on COVID-19 infections.

We used Census population estimates as of July 1, 2020; we did not use time-varying estimates due to the limited time period; these in any case would not affect the Pfizer-versus-Moderna comparisons. We used IHIE data to count vaccinees and subtracted the vaccinee counts from the total population to determine the number of unvaccinated individuals in each group and time period. We used text analysis of death certificates to identify deaths due to COVID-19. COVID-19 death counts were similar using ICD-10 codes based on the text fields. (Supplementary Table S1). We excluded immunocompromised decedents.

We considered vaccination to be effective against mortality starting 30 days after receipt; this allows both time (1–2 weeks) for the vaccine dose to become fully effective, and a lag (typically 2–3 weeks) between infection and death. We counted persons vaccinated with a given number of doses as of the first of each month, allowing for this lag. Our results are not sensitive to the lag period.

We excluded immune-compromised persons, identified either from death records or because they received a third dose before a third dose was authorized for non-immune-compromised persons.

U.S. vaccination rules required persons to receive either Pfizer or Moderna for their first two doses. For both, primary vaccination consisted of two doses, at least 21 days apart for Pfizer and 28 days apart for Moderna. Crossover was allowed for the booster dose, but remained uncommon; 8.7% of booster recipients received mixed Pfizer and Moderna vaccines. We treat persons who received one to three vaccine doses as vaccinated with Pfizer (P) or Moderna (M) if they received only Pfizer or only Moderna. We treat persons who received four vaccine doses as vaccinated with P or M if the first three doses were all P or all M. We exclude a small number of persons with other vaccination patterns (see Supplementary Table S2 for counts). The final sample includes 665,961 Pfizer and 574,779 Moderna vaccinees as of year-end 2022 (Table 1).

Table 1 provides summary statistics for Indiana vaccinees and unvaccinated persons, aged 60+. The total estimated population is 1,545,080 persons. Vaccinee counts are at the end of each indicated period; deaths are totals for each period; and ratios are averages for the period. For example, COVID-MR = (COVID-19 deaths/(beginning population + ending population)/2). Supplementary Table S3 provides a similar table for ages 15–59. Note that the total population slightly exceeds the sum of the numbers unvaccinated and with one to two or 3+ doses due to a few persons with unusual vaccination patterns. CEMP, RMR, and HVBCF are for all vaccinated persons, with any number of doses.

### 2.2. Variables and Regression Methods

We define CEMP for persons with *x* doses of vaccine type *v*, (*CEMP_x,v_*), where *v* = P or M, as

$$CEMP_{x,v}=100\times \frac{COVID\;deaths_{x,v}}{non-COVID\;natural\;deaths_{x,v}}$$

We calculate vaccinee relative mortality risk (RMR), that is, the fractional increase in deaths due to COVID in vaccinees relative to unvaccinated persons as

$${RMR}_{xv,unvax}=\frac{CEMP_{x,v}\;}{CEMP_{unvax}}$$

$An\;RMR_{xv,unvax}$ of zero implies perfect vaccine protection; RMR of 1.0 implies no protection.

Many studies report vaccine effectiveness (VE) against COVID-19-related death, which is the fractional reduction in mortality due to vaccination, instead of RMR. The two are closely related: VE = 1 − RMR.

We can also measure relative RMR for Pfizer versus Moderna:

$$MR_{P,M}^{x}=\frac{CEMP_{x,P}\;}{CEMP_{x,M}}$$

A relative $RMR_{P,M}$ of 1.0 implies similar protection from each vaccine. A relative $RMR_{P,M}$ greater than 1.0 implies less protection from the Pfizer than the Moderna vaccine. Note that one can also estimate ${RMR}_{xv,unvax}$ or $RMR_{P,M}$ as an odds ratio from logistic regression for a sample of decedents containing both the decedents in the numerator and the decedents in the denominator.

It is useful to also define a measure of HVB. Let NCNMR be the non-COVID natural mortality rate for a group with x doses of vaccine type *v*:

$$NCNMR_{x,v}=\frac{non-COVID\;natural\;deaths_{x,v}}{Pop_{x,v}}$$

We can also define a healthy-vaccinee bias *correction factor* (HVB_CF), which equals 1.0 if there is no selection bias. Lower ratios, below 1.0, indicate increasing bias: vaccinated people are healthier than unvaccinated people, and thus have lower NCNMR rates:

$$HVB\text{\_}CF_{xv,unvax}=\frac{NCNMR_{x,v}}{NCNMR_{unvax}}$$

This measure assumes that COVID-19 vaccination does not strongly affect non-COVID natural mortality.

The remaining steps are not directly used in our analysis, but may be helpful in understanding the connection between the CEMP-based RMR that we rely on, a “raw” RMR measure that is not adjusted for background health, and healthy-vaccinee bias, and thus the importance of addressing healthy-vaccinee bias. Define *RMR*^raw^, based on unadjusted COVID-19 mortality rates (*COVIDMR*), as

$$RMR_{xv,unvax}^{raw}=\frac{COVIDMR_{xv}}{COVIDMR_{unvax}}$$

CEMP-based *RMR*^CEMP^, *RMR*^raw^, and *HVB_CF* are algebraically related; see Supplementary Material for derivation:

$$RMR_{xv,unvax}^{CEMP}=\frac{RMR_{xv,unvax}^{raw}}{HVB\text{\_}CF_{xv,unvax}}$$

For example, if raw vaccine effectiveness against death is 90% (RMR of 0.10 versus the unvaccinated), and *HVB_CF* is 0.5 (vaccinees have half the background mortality risk of the unvaccinated), then $RMR^{CEMP}=\frac{0.1}{0.5}=0.20.$ (twice the raw rate).

### 2.3. Logistic Regression and Confidence Intervals

$RMR_{xv,unvax}$ and $RMR_{P,M}$ are ratios of CEMPs for two groups. CEMP, in turn, is the odds, for a sample of decedents, of dying from COVID-19 versus other natural causes. The RMRs are thus odds ratios. They can be obtained algebraically, as above, or from logistic regression on a sample of decedents. We use logistic regression to obtain 95% confidence intervals (CIs) for RMRs on groups of decedents defined by age and vaccination status. We obtain confidence intervals for $HVB\text{\_}CF_{xv,unvax}$ using Poisson regression.

In addition to the univariate estimates of RMR, described above, we also conducted a multivariate logistic estimation of $RMR_{x,unvax}^{CEMP}$, in which we adjusted for other predictors of mortality risk, available from death certificates: age, age^2^, sex, race/ethnicity, education level, marital status, and area-SES. See Supplementary Section S6.B for details.

### 2.4. Assessing the CEMP Approach as a Correction for Healthy-Vaccinee Bias

The CEMP approach uses NCNMR as a reasonable but imperfect proxy for the overall health of a given group, and thus the likelihood of mortality if not vaccinated. We assessed the validity of this approach in several ways. An initial step involved comparing mortality from all natural causes in the pre-COVID period from April–December 2019 to COVID-19 mortality over the same months in 2020. A further step involved conducting a multivariate logistic estimation of ${RMR}_{x,unvax}$ in which we adjusted for other predictors of mortality risk, available from death certificates, namely age, age^2^, sex, race/ethnicity, education level, marital status, and zip-code level socioeconomic status, measured using the Graham Social Deprivation Index (zip-SES) [29].

### 2.5. Counting Vaccine Doses

Our dataset, obtained from IHIE, contains vaccinees matched to death records. However, we do not know the matching algorithm that IHIE uses. There is evidence in the IHIE data that IHIE sometimes combined two vaccinated people into a single person record. This appears to affect 1–2% of vaccinees. In Supplementary Material Section S3, we develop rules to estimate the number of doses received by an actual person.

### 2.6. Core Study Period

We studied the period from 1Q2021 to 4Q2022, but focused on the “core” period, from 2Q2021 to 1Q2022. We excluded 1Q2021 for several reasons; see Supplementary Section S5.B for additional discussion. The primary reason was limited vaccine availability. In 1Q2021, the mRNA vaccines were in limited supply and were being rolled out with complex priority rules. Priority was given to healthcare workers, other first responders, older persons, and other persons at higher risk for severe COVID-19. Local governments set their own local priority rules; for example, allowing vaccination above age 80 through date X, then above age 78 through date Y, etc. The vaccination priority rules were generally dropped during April 2021. Thus, 2Q2021 is the first quarter with widespread vaccine availability.

We ended the core period with 1Q2022, based on much lower mortality rates in 2Q2022, leading to wide confidence intervals, and evidence that RMR versus the unvaccinated was substantial through 1Q2022, but small and statistically insignificant thereafter [8]. After the large Omicron wave in 1Q2022, a large proportion of vaccinees were also already infected. Thus, the Pfizer-vs-Moderna comparison had become, for many people, a comparison between two types of hybrid immunity, namely Pfizer-plus-prior-infection vs. Moderna-plus-prior-infection, where the protection provided by prior infection would likely mute any Pfizer-vs-Moderna difference. We in fact found no evidence for a meaningful Moderna advantage from 2Q2021 on.

### 2.7. Subsample Analyses

In the text, we report analyses for two broad age groups: 15–59 (3,914,000 persons) and 60+ (1,545,000 persons). We also conduct analyses separately for ages 60–79 (1,285,000 persons) and 80+ (260,000 persons), and separately for men (48.9% of the overall sample) and women.

### 2.8. Possible Sources of Bias

There is evidence of undercounting of COVID-19 deaths in the U.S. [30,31]. We also find some evidence of undercounting in Indiana (Supplementary Figure S4). This implies that CEMP values are too low. However, RMR estimates will be biased only if undercounting differs between the two comparison groups (vaccinated-versus-unvaccinated persons, or Pfizer-versus-Moderna vaccinees). We have no reason to expect overall undercounting to produce relative Pfizer-versus-Moderna bias, but cannot assess possible bias with our data.

Second, we observe the undercounting of vaccinee deaths over August–October 2021, presumably due to IHIE failing to fully match vaccinee records to mortality records during the period (Supplementary Section S3, Figure S5). An undercount of vaccinee deaths implies overcount for unvaccinated persons. If vaccination reduces COVID-19 mortality, these overcounted deaths of unvaccinated persons will disproportionately involve non-COVID causes. This will cause downward bias in CEMP_unvax_ and upward bias in $RMR_{xv,unvax}$ = $\frac{CEMP_{xv}\;}{CEMP_{unvax}}$. We address this issue by adjusting for the apparent undercounting. In brief, we interpolate counts of vaccinated decedents from July 2021 to November 2021, to obtain adjusted counts. We increase the monthly numbers of vaccinated deaths to follow the interpolation line and correspondingly decrease unvaccinated deaths. We assume that the missing vaccinee deaths reflect random matching error, unrelated to the cause of death or which vaccine someone received. See Supplementary Section S3. A for details. The corrections are small in percentage terms. Moreover, these matching errors will not affect the Pfizer-versus-Moderna comparison, unless the matching errors were somehow associated with vaccine type.

Third, we learned in 2021 that mRNA vaccine effectiveness was subject to rapid waning; this gave rise to the approval of booster doses. Bias could arise if there were timing differences in Pfizer-versus-Moderna uptake, relative to peak COVID-19 periods. However, we found little difference in uptake timing (Supplementary Figure S15).

Fourth, we excluded immune-compromised persons, but have no reason to believe that this exclusion would have lead to bias for the Pfizer-versus-Moderna comparison.

## 3. Results

### 3.1. Assessing the CEMP Measure

CEMP relies on group NCNMR as a reasonable proxy for group health, and thus risk of COVID-19 mortality. We confirmed in prior work that NCNMR correlates strongly with COVID-19 mortality [6,7,8,13,14,15]. We provide a summary here, plus additional evidence specific to the Pfizer-versus-Moderna comparison. Supplementary Figure S1 shows the strong correlation between natural mortality counts in Indiana in April–December 2019 (pre-COVID period) and COVID-19 mortality counts in the same months of 2020 (COVID period, but pre-vaccine) for groups defined by age (15–39, 40–49, 50–59, 60–69, 70–79, 80–89, and 90+), sex, race/ethnicity, and quintiles of zip-code level socioeconomic status measured using the Social Deprivation Index (zip-SES) [29]. Non-COVID natural mortality strongly predicts COVID mortality for unvaccinated persons (Pearson correlation coefficient r = 0.963). Supplementary Figure S2 shows a similar correlation for *rates* (instead of counts); r = 0.948. Supplementary Figure S3 confirms that all-cause natural mortality in Indiana in 2019 is highly correlated with NCNMR in 2020 (r = 0.999).

We also conducted multivariate logistic regression in which we estimated ${RMR}_{x,unvax}$ controlling for age, age^2^, sex, race/ethnicity, education level, marital status, and area-SES. RMR estimates, within groups defined by age and time period, were very similar to univariate CEMP-based estimates, with overlapping confidence intervals (Supplementary Table S7). For example, for 3Q2021, the CEMP-based Pfizer/Moderna RMR ratio for primary vaccination is 1.90; the multivariate estimate is 1.94. The same is true for multivariate versus univariate estimates of the Pfizer/Moderna RMR ratio (Supplementary Table S8). This provides evidence that using CEMP as an outcome does a good job of adjusting for the association between these other predictors and COVID-19 mortality. We caution that the group NCNMR provides only a rough proxy for individual-level COVID-19 risk.

### 3.2. Summary Statistics

Table 1 provides quarterly summary statistics for ages 60+ over the sample period. By year-end 2022, around 70% of Indiana residents aged 15+ had received 1+ vaccine doses (Supplementary Table S2), including over 90% of people aged 60+. Most vaccinated persons received a booster (Table 1). Supplementary Figure S6 provides additional information on vaccination rates over time. COVID-19 deaths, and thus CEMP, fell sharply beginning in 2Q-2022; while $RMR_{vax,unvax}^{CEMP}$ rose.

### 3.3. Evidence on Healthy-Vaccinee Bias

**Figure 1:**
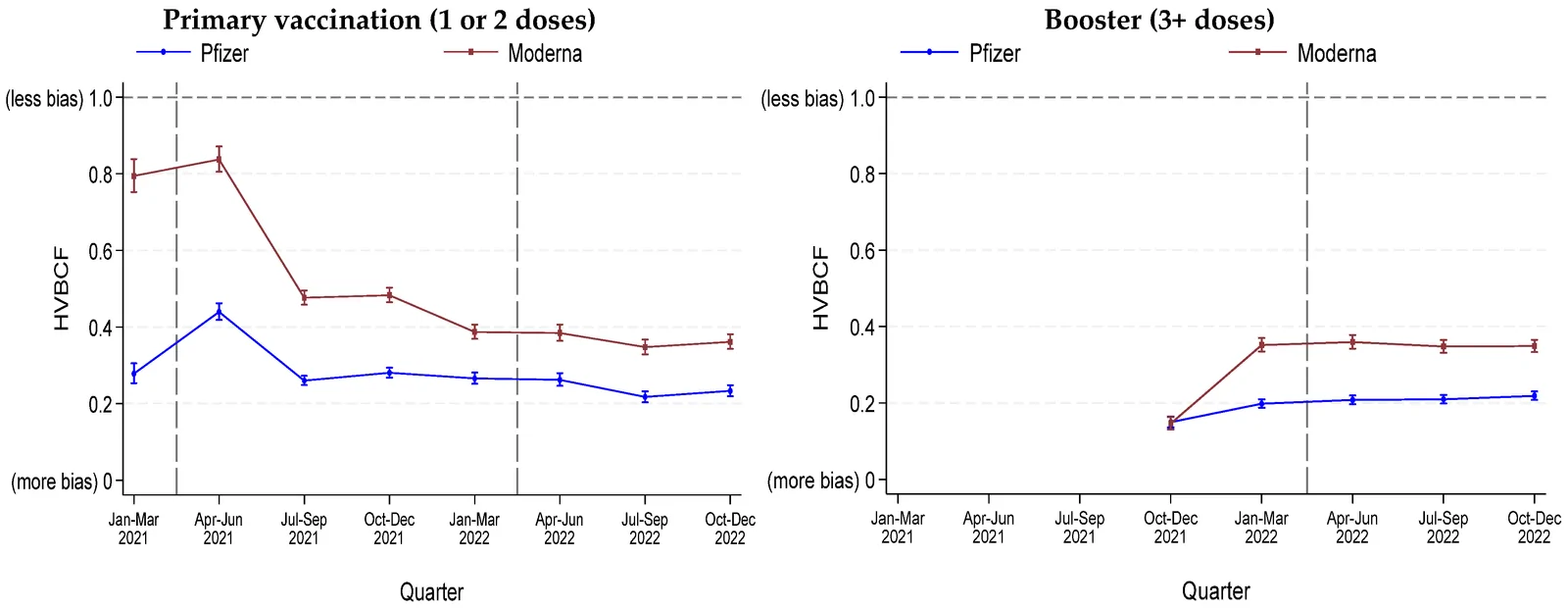
Healthy-Vaccinee Bias Correction Factor (Ages 60+). Figure shows healthy-vaccinee bias correction factor (HVB_CF), separately for Pfizer and Moderna vaccinees aged 60+, for primary vaccination (left-hand graph) and booster (right-hand graph) by quarter over the sample period. HVB_CF is ratio of non-COVID NMR for vaccinated (with indicated number of doses)/non-COVID NMR for unvaccinated. Estimates for 3Q–4Q2021 are adjusted for IHIE undercount of vaccinated decedents during this period. See Supplementary Table S4 for underlying data. Sample excludes immune-compromised persons. Vertical lines show 95% confidence intervals. Vertical dashed lines show start and end of core, vaccine-available, high-mortality period (2Q2021–1Q2022).

Figure 1 provides $HVB\text{\_}CF_{x,unvax}$ values for ages 60+. See Supplementary Table S3 and Figure S8 for ages 15–59. HVB was substantial for both primary and booster vaccination, with tight CIs. It was larger (lower HVB_CF) for Pfizer than Moderna. Thus, vaccinees were healthier than the unvaccinated, and Pfizer vaccinees were healthier than Moderna vaccinees.

### 3.4. Pfizer-Versus-Moderna RMRs for Ages 60+

**Figure 2:**
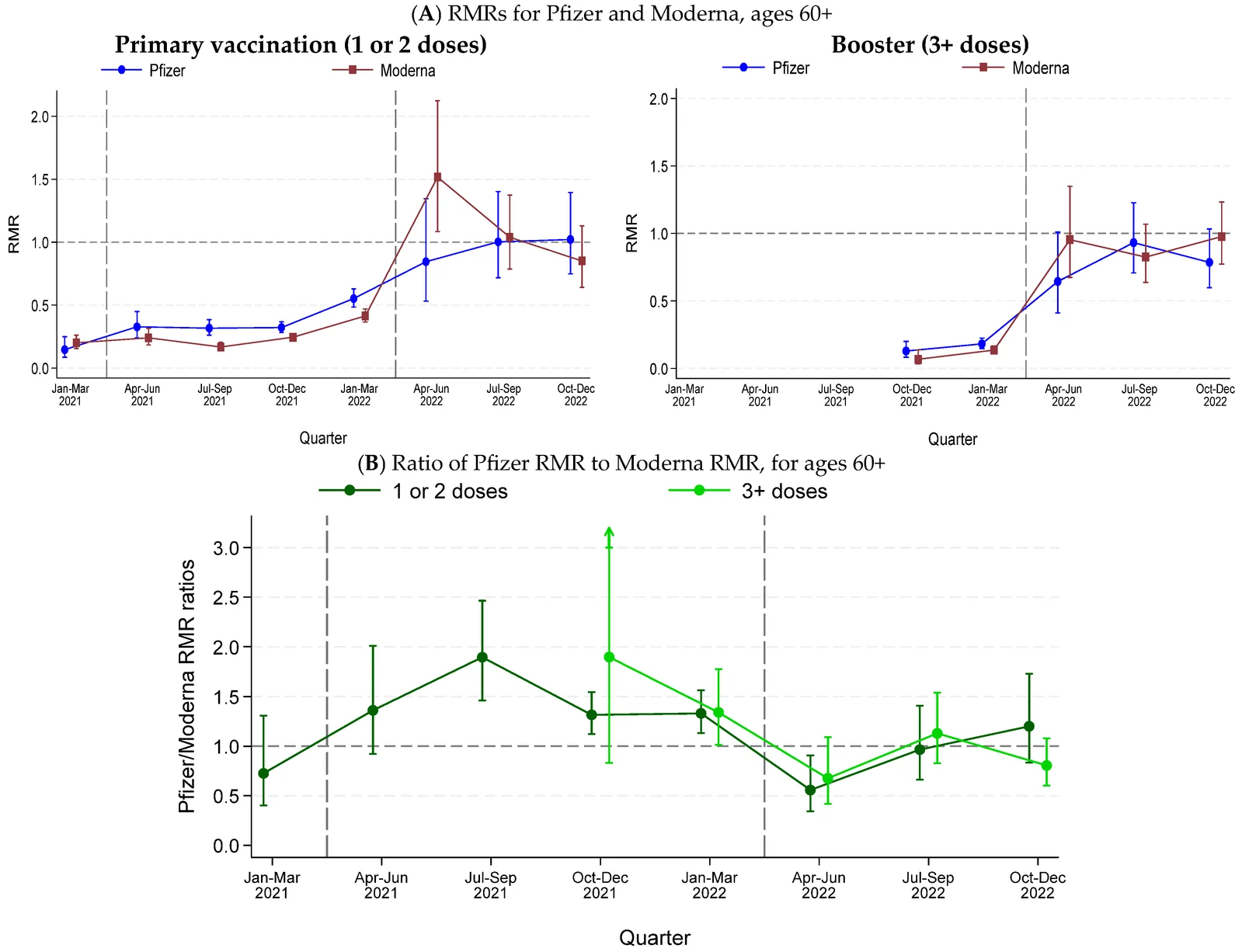
Remaining mortality risk for Pfizer and Moderna, ages 60+. Figure shows remaining mortality risk (RMR), based on CEMP, by quarter over 1Q2021–4Q2022 for Pfizer and Moderna vaccinees aged 60+ with primary vaccination (1–2 doses) (left-hand graph) or booster (3+ doses) (right-hand graph). (**A**) RMR, measured separately for Pfizer and Moderna vaccinees. (**B**) Pfizer/Moderna ratio of RMRs. (**A**,**B**) Estimates for 3Q-4Q2021 are adjusted for IHIE undercount of vaccinated decedents during August–October 2021. Sample excludes immunocompromised persons. Vertical lines show 95% confidence intervals. Vertical dashed lines show start and end of core vaccine-available, high-mortality period (2Q2021–1Q2022).

Figure 2A, presents quarterly RMR values of Pfizer and Moderna vaccinees aged 60+, separately for vaccinees who received only primary vaccination (left panel) or also received a booster dose (right panel). Figure 2B converts the Pfizer and Moderna RMR values to a Pfizer/Moderna ratio. For primary vaccination, this ratio is above 1.0 throughout the core period. Over the full core period, the average Pfizer/Moderna RMR ratio for persons receiving only primary vaccination is 1.49 [95% CI 1.35, 1.65]. For booster recipients, we have data only for 4Q2021 and 1Q2022. Over both quarters taken together, the Pfizer/Moderna ratio is 1.34 [CI 1.03, 1.75].

There was substantial evolution of virus variants over the core period, with the Alpha variant dominant in 2Q2021, Delta in 3Q-4Q2021, and Omicron beginning in 1Q2022. One factor affecting RMR for both vaccines over this period could be the change in dominant strain. A second factor was waning VE for the mRNA vaccines, beginning roughly 5–6 months after primary vaccination. Evidence of waning led to booster approval in late September 2021, initially for older and other high-risk persons.

An important factor that likely affected RMR time trends is rapid growth in the number of already infected people in 1Q2022 with the huge Omicron infection wave. By the end of 1Q2022, roughly half of vaccinated persons had been infected, and thus had hybrid immunity, and a large percentage of unvaccinated persons had prior-infection-based immunity [32]. Thus, RMR for vaccinated-versus-unvaccinated persons increasingly involved comparing a mix of hybrid and vaccine-only immunity for vaccinees to infection-based immunity for most unvaccinated persons.

Against that background, consider RMR for primary vaccination. Primary vaccination has substantial protective effect through 1Q2022 for both vaccines (left-hand graph). However, during the core period, RMR was consistently higher for Pfizer than for Moderna. A booster dose provided additional protection for both vaccines, but the Moderna-to-Pfizer gap remains.

RMRs increase somewhat for both vaccines in 1Q2022 and increase substantially after that, for both age groups. Point estimates for primary vaccination are close to 1.00 (no protective effect), and indeed above 1.0 in 2Q2022. The wider CIs reflect lower COVID-19 mortality rates. Supplementary Figures S10–S12 provide separate estimates for ages 60–79 and 80+, and for first versus second booster dose.

### 3.5. Pfizer-Versus-Moderna RMRs for Ages 15–59

**Figure 3:**
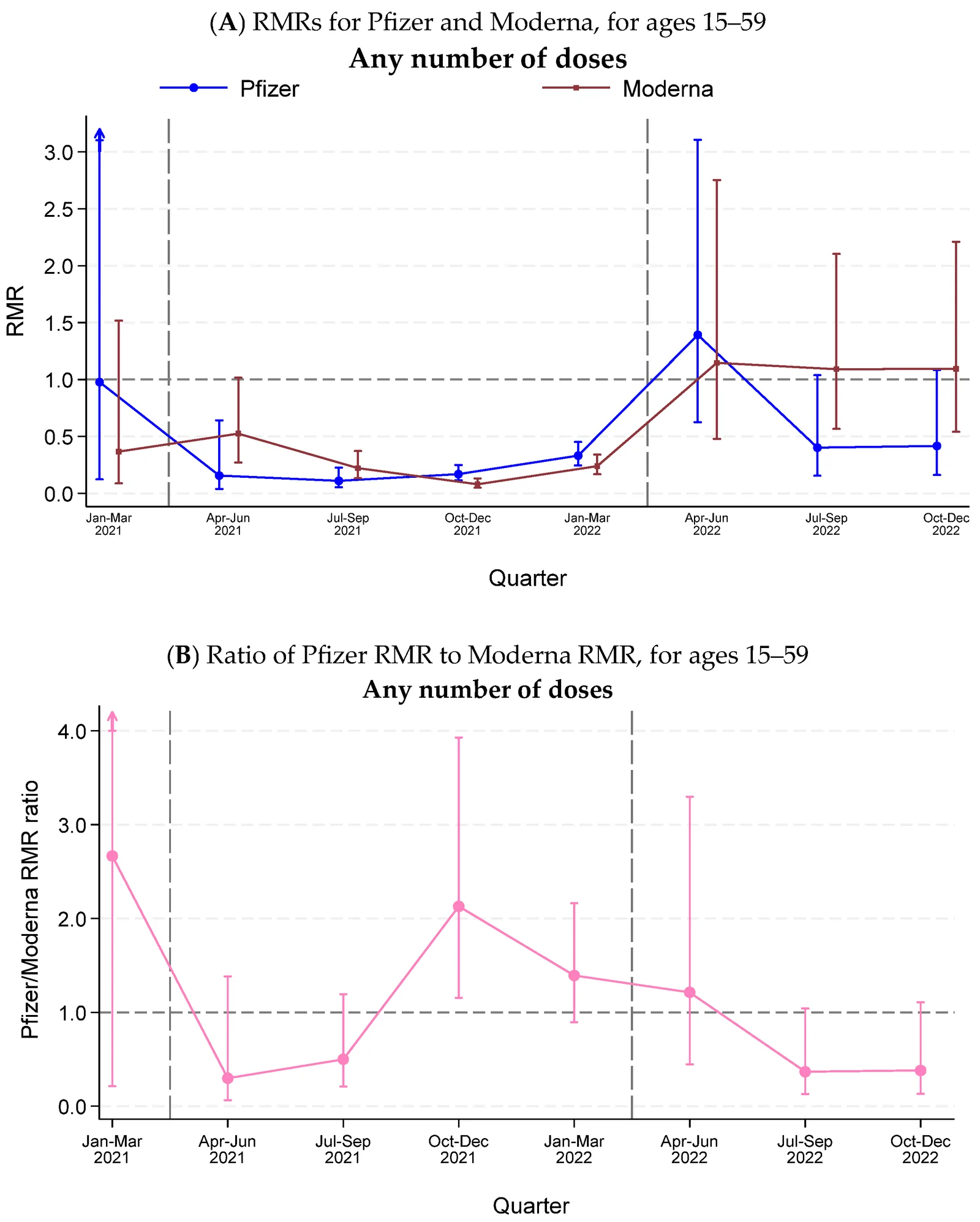
Remaining mortality risk for Pfizer and Moderna, ages 15–59. The figure is similar to Figure 2, except for age group, and shows remaining mortality risk (RMR), based on CEMP, by quarter over 1Q2021–4Q2022 for Pfizer and Moderna vaccinees aged 15–59 with any number of vaccine doses. (**A**) RMR separately for Pfizer and Moderna vaccinees. (**B**) Pfizer/Moderna ratio of RMRs. (**A**,**B**) Estimates for 3Q–4Q2021 are adjusted for IHIE undercount of vaccinated decedents during August–October 2021. The sample excludes immunocompromised persons. Vertical lines show 95% confidence intervals. Vertical dotted lines show the start and end of vaccine-available, high-mortality period (2Q2021–1Q2022).

We turn in Figure 3 to ages 15–59. Due to the small number of booster recipients prior to 1Q2022, we show results for persons receiving any number of doses. For this age group, there is suggestive but weaker evidence for a Moderna advantage. In the first two of the four quarters within the core period, the Pfizer–Moderna ratio is below 1.0, but these are relatively low mortality periods. In the next two periods, this ratio is above 1.0, and is statistically significant in 4Q 2021. Over the full core period, the Pfizer/Moderna ratio for primary vaccination is 1.25 [CI = 0.91, 1.72], and for any number of doses is 1.26 [CI = 0.93, 1.72]. Due to the wider CIs for younger persons, we also cannot say that the Pfizer/Moderna ratio is lower overall for younger versus older persons; instead, the CIs overlap for the two age groups.

### 3.6. Multivariate Estimates

Supplementary Table S8 reports separate estimates of $RMR_{xv,unvax}$ for Pfizer and Moderna from a multivariate logistic model for vaccinees receiving different numbers of doses, as well as the Pfizer/Moderna ratio. Adding additional predictors has almost no effect on this ratio (Supplementary Figure S16). We caution that this analysis is limited to covariates available from death certificates and does not rule out unobserved differences between Moderna and Pfizer vaccinees.

### 3.7. Effect of Differential Vaccine Waning

**Figure 4:**
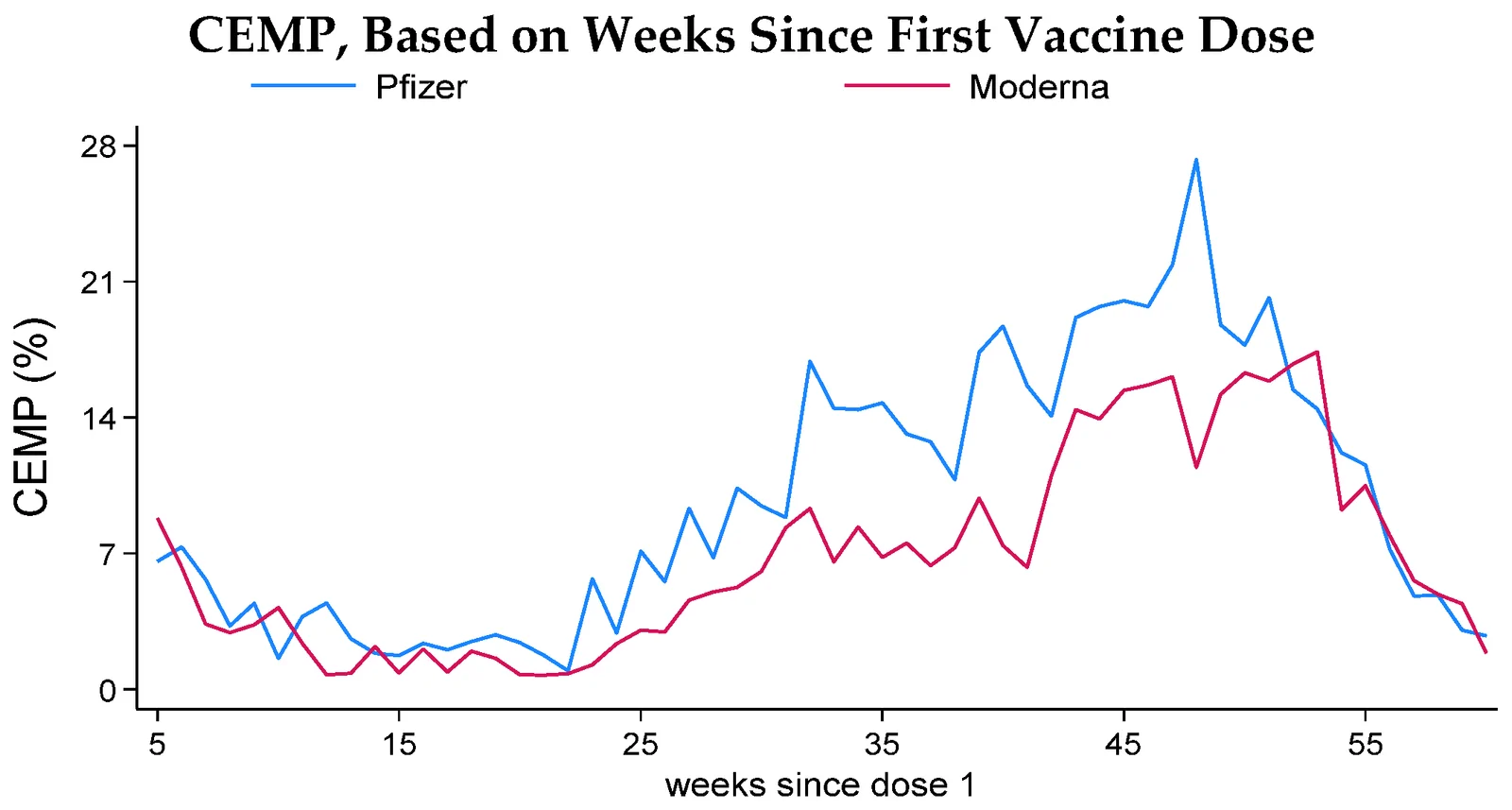
CEMP for Pfizer vs. Moderna and time since first dose, ages 60+. Figure shows CEMP, separately for Pfizer and Moderna, for vaccinees aged 60+ who received their first vaccine dose in 1Q2021, as function of weeks since receipt of first vaccine dose. Sample includes persons with any number of doses.

There is evidence that Pfizer vaccine effectiveness waned more rapidly than Moderna ([2], review). We explore in Figure 4 the extent to which faster waning can explain part of the overall Moderna-versus-Pfizer difference. We first limit the sample to vaccinees aged 60+ who received their first dose in 1Q2021, the time period when most older persons received their first and often their second dose (Supplementary Figure S15). Figure 4 shows CEMP for this cohort based on time since first dose, starting in week 5 (this allows a lag between vaccination and death, similar to the 30-day lag in our main analysis).

For roughly 20 weeks after first dose, Pfizer CEMP levels are generally above Moderna levels, but only modestly so. CEMP levels for the two vaccines diverge more strongly over weeks 20–55. This is, roughly speaking, the period from when significant waning began to the end of the 1Q2022 Omicron wave. Faster Pfizer waning thus plausibly explains a substantial part of the overall Pfizer-versus-Moderna difference in RMR. Supplementary Figure S17 shows a similar pattern for ages 15–59.

### 3.8. Additional Analyses

In Supplementary Figures S13 and S14, we reproduce Figure 1 and Figure 2, separately for men and women. Results for the Pfizer/Moderna ratio are similar for both sexes.

In Supplementary Figure S18, instead of excluding people receiving mixed vaccine types, we treat persons receiving mixed vaccine types as Pfizer primary vaccination and a Moderna booster as Pfizer vaccinees, and persons receiving Moderna primary vaccination and a Pfizer booster as Moderna vaccinees, instead of excluding these persons. Results for the Pfizer/Moderna ratio are similar to Figure 2.

## 4. Discussion

The general belief in the public health community is that the Pfizer and Moderna mRNA vaccines were both highly effective against severe COVID-19 disease, including death. No public health agency, to our knowledge, has stated that one is preferred over the other; the U.S. and U.K. agencies state that they provide similar protection [33,34]. However, most prior comparative studies fail to control effectively for HVB. We found, in our own prior study of Milwaukee [6], that once one controls for HVB, Moderna emerges as being substantially more effective against death for ages 60+. We sought in this study to confirm (or not) that apparent advantage, and if so, for which age groups, using a much larger, population-representative sample; our sample is also much larger than the VA-based study by Dickerman et al. [3]. We confirmed a clinically important Moderna advantage, although with magnitude well below that we had found in Milwaukee.

### 4.1. The Importance of HVB and a Credible Counterfactual

To estimate VE and RMR, one must start with a credible counterfactual: how many COVID-19 vaccinees would have died of COVID-19 if they were not vaccinated? We found large differences in background mortality risk between vaccinated and unvaccinated persons, and also between Pfizer and Moderna vaccinees. Pfizer vaccinees were healthier than Moderna vaccinees among both ages 60+ and ages 15–59, and for both primary and booster vaccination. In prior work, we found a similar Pfizer/Moderna difference in background health in Milwaukee County, Wisconsin [6].

We do not know why Pfizer vaccinees were generally healthier than Moderna vaccinees in both Indiana and Wisconsin. This difference may reflect specific, unobserved aspects of vaccine rollout in these two areas. This pattern is not universal. In contrast, a study of VA vaccinees by Dickerman et al. found no significant difference in non-COVID-19 mortality between Pfizer and Moderna vaccinees, but studied only veterans vaccinated at VA centers that used both Pfizer and Moderna [3].

HVB can produce large biases in estimating VE and RMR. CEMP has important advantages as a way to control for HVB. It relies only on death certificates, which are available for all decedents, and does not depend on population estimates. NCNMR performed well predicting COVID-19 mortality during the pre-vaccine period (Supplementary Figures S1 and S2). This and our other checks provide evidence that CEMP-based RMR provides a good estimate relative to counterfactual mortality for the same persons, if unvaccinated. An alternative approach, involving controlling for individual health, would require population-level health data, which is not available in U.S. datasets. Moreover, Chemaitelly et al. find that even with extensive health data, substantial HVB remains [28].

The HVB magnitudes we found are consistent with our Milwaukee study and with evidence from Czechia that the unvaccinated have all-cause mortality two to three times higher than for vaccinees [9]. Large differences between vaccinated and unvaccinated persons also exist for non-natural mortality. HVB can also explain differences in healthcare spending for vaccinated-versus-unvaccinated persons [35].

### 4.2. Prior Literature Comparing Pfizer to Moderna

The prior study most comparable to this one is our own study of Milwaukee [6]. We found similar RMR for ages 18–59, but also that for ages 60+, RMR for Pfizer primary vaccination was 248% of Moderna [95% CI = 175%, 353%]; in the Omicron period, Pfizer RMR was 57% versus 23% for Moderna. Some other comparative studies of VE against death find Pfizer–Moderna differences, but do not highlight them. Among U.S. studies, Robles–Fontan et al. [36] studied Puerto Rico through mid-October 2021. They report two-dose RMRs after 144 days (longest period considered) of 14% for Pfizer and 7% for Moderna. However, their abstract states only that “[Both] vaccines were highly effective across all age groups.” There are several studies of U.S. veterans, but none extend beyond September 2021. Ioannou et al. report that Ioannou et al. report that Moderna vaccination is associated with lower hospitalization risk (adjusted relative hazard ratio (aHR) 0.633 (CI 0.562–0.713) and likely against death although the mortality estimate is not statistically significant (aHR 0.808, 95% CI 0.592–1.103) [5]. Dickerman et al. report lower Moderna hospitalization rates but similar mortality rates [3]; Cohn et al. found similar mortality rates [4]. Mayr et al. report a Moderna advantage in reducing hospitalization risk, and an apparent advantage for a combined ICU-or-death outcome, but the small sample size “precluded statistically significant comparisons.” [24].

Outside the U.S., Lytras et al. study Greece through year-end 2021 and find a nearly 3:1 Moderna advantage against mortality, but only in a supplemental figure; the text (at 5048) reports “[o]nly marginal differences [between vaccines] in effectiveness [37].” A Czechia study through November 2021 reports two-dose RMR, 7–8 months after vaccination, of 17% for Pfizer vs. 12% for Moderna [38].

The review by Black and Thaw reports a Moderna advantage against mortality after waning (at least 120 days after vaccination) during the Delta-dominant period, with midpoint RMR estimates from multiple studies of 13.3% for Pfizer vs. 9.2% for Moderna [2]. Other studies find smaller differences.

### 4.3. Moderna-Versus-Pfizer Differences in RMR

We find the following for ages 60+: (i) similar to Milwaukee, Pfizer vaccinees were healthier than Moderna vaccinees; and (ii) over the core period of 2Q2021–1Q2022, Moderna was associated with lower risk of COVID-19 mortality than Pfizer. For younger people (ages 15–59), COVID-19 deaths were much lower, and CIs therefore larger. Our point estimates for younger persons suggest a Moderna advantage. The CI includes both 1.0 (no advantage) and the point estimate for ages 60+ (similar advantage).

After 1Q2022, we found much higher RMRs for both vaccines, no significant difference between them, and indeed no statistically significant vaccine protection relative to unvaccinated persons, as previously reported [8], although with wide CIs that do not rule out some protective effect. Higher RMRs likely reflect the changing nature of the comparison between vaccinated and unvaccinated. By 2Q2022, many vaccinated persons and an even larger percentage of unvaccinated persons had already been infected and survived [32]. Thus, VE during this period largely reflected the added protection from hybrid immunity (prior infection plus vaccination) relative to natural immunity alone. Immune imprinting from vaccination, in which prior vaccination reduces response to variants not included in the original vaccine, could also play a role, where imprinting could plausibly have a larger effect on response to a new vaccine variant for Moderna due to higher dose.

### 4.4. Benefit vs. Risk for Different Age Groups

One explanation for a Pfizer-versus-Moderna difference, especially for older people, is that older people may need a larger dose for full protection [39]. This would be similar to the flu vaccine, for which a higher dose is recommended for ages 65+ [40]. The higher Moderna dose could also help to explain slower waning [2].

At the same time, the lower Pfizer dose had a lower incidence of both short-term side effects, such as fever [41,42,43], and rare but potentially serious side effects, especially myocarditis and pericarditis, principally for younger men [44,45,46,47]. The difference in dosage provides a plausible explanation for these risk differences.

These differences in relative benefit and risk suggest the need for nuance in vaccine recommendations, which is lacking in public health messaging. For example, U.S. guidance states that “There is no preference for one vaccine over the other when [both are approved]” [33]. The UK Joint Committee on Vaccination and Immunization (JCVI) similarly reported “that the data suggests minimal differences in vaccine effectiveness.” [34].

### 4.5. The Role of Faster Pfizer Waning

One reason for lower Moderna-versus-Pfizer RMR appears to be the faster waning of primary Pfizer vaccination (Figure 4). At the same time, the Pfizer-to-Moderna RMR ratio was probably above 1.0 in 2Q2021, before significant waning occurred (point estimate = 1.36 [CI = 0.92, 2.01]). Thus, the overall Pfizer-versus-Moderna difference likely reflects both a “native” difference and a difference due to faster-waning. We judged that we lacked sufficient data to estimate the relative contributions of these two effects.

### 4.6. Possible Channels for the Overall Pfizer-Versus-Moderna Difference

A number of possible channels could have contributed to the overall Pfizer-versus- Moderna difference in mortality that we observe. Pfizer vaccinees could have faced higher infection risk, especially after waning occurred; infection transmission rates could have been higher within Pfizer than within Moderna-vaccinated households; and Pfizer vaccinees could have faced more severe infection if infected. Each of these channels could have been affected by differential waning. We lack infection data, so cannot distinguish between them.

## 5. Limitations

This study has a number of limitations. We study only mortality. Results could be different for other measures of severe disease, such as hospitalization or ICU admission, or for risk of long COVID. We also lack data on COVID-19 infection rates.

We have data only for Indiana, which may not be representative of other areas. However, we also found higher Pfizer-versus-Moderna RMRs in Milwaukee [6], and several other studies have found Pfizer-versus-Moderna differences (see Section 4.2).

We have data only through 2022. We found that both the vaccinated-versus-unvaccinated and the Moderna-versus-Pfizer differences were small and insignificant for 2Q–4Q2022 after the peak of the Omicron wave. Thus, whether Moderna booster doses retain an advantage today, and if so for whom, is necessarily speculative.

We have data on zip-SES but not individual SES.

We use NCNMR as a reasonable but imperfect proxy for COVID-19 mortality risk for a group of persons. We lack data on many individual risk factors, including comorbidities, health history, and nursing home residence. Moreover, CEMP is a proxy for group risk, but does not measure individual risk.

The CEMP measure assumes that vaccination does not meaningfully affect non-COVID mortality [22,48,49,50]. It is known that COVID-19 infection predicts higher near-term mortality from other causes [51,52]. However, we have no reason to expect that these effects will affect the Pfizer/Moderna ratio.

Some COVID-19 deaths may be coded as non-COVID-19 natural deaths (Supplementary Figure S4). This miscoding will reduce CEMP estimates, but we again have no reason to expect bias in the Pfizer/Moderna ratio.

We studied only the two mRNA vaccines, not Johnson & Johnson or Novavax.

Our assessment of underlying health does not control for behavioral differences between the vaccinated and unvaccinated, or between the on-average-healthier people receiving Pfizer and the on-average-less-healthy people receiving Moderna vaccination.

Finally, we report association, based on observational data, not causation. A true causal design would require either exogenous assignment of some people to receive Pfizer and others to receive Moderna, or much more complete information about individual health and other characteristics. Confounding may exist due to unobserved variables that are correlated both with COVID-19 mortality and vaccine type, but are not captured by our use of NCNMR to proxy for background health.

## 6. Conclusions

We provide two principal results. First, we provide evidence that when comparing different vaccines, it is crucial to address selection effects for which people receive which vaccine. Second, we report evidence that Moderna vaccination was associated with lower mortality risk than Pfizer vaccination, especially for ages 60+, during the core period we study. If confirmed for a more recent period, this would suggest that advice for older persons to obtain periodic boosters should include the discussion of the evidence for a Moderna-versus-Pfizer difference. More nuance is needed for persons aged 15–59. Our point estimate of 1.25 is only slightly below the 1.34 estimate for older adults, but has a much wider confidence interval and is not statistically significant [95% CI = 0.91, 1.72]. Our evidence suggests a need for more nuanced guidance on the choice between vaccines, including guidance that reflects differences based on recipient age, and perhaps sex (for myocarditis/pericarditis risk).

To assess the merits of Pfizer and Moderna boosters in the current environment, there is a need for similar studies that control for HVB, cover more recent periods, and focus on vulnerable populations, such as older and immune-compromised persons. At the same time, low COVID-19 mortality rates limit statistical power. Thus, finding statistically significant differences will likely require studying very large populations.

## Figures and Tables

**Table 1:** Summary statistics; ages 60+.

	1Q 2021	2Q 2021	3Q 2021	4Q 2021	1Q 2022	2Q 2022	3Q 2022	4Q 2022	Core Period	Total/Average
**Unvaccinated**	808,818	353,970	287,825	222,340	194,969	187,943	183,482	182,314	194,969	182,314
COVID-19 deaths	2348	380	1021	1819	1605	85	169	184	4825	7611
Non-COVID natural deaths	11,229	6101	5413	5247	4762	4008	4205	4549	21,523	45,514
COVID-MR	0.20%	0.07%	0.32%	0.71%	0.77%	0.04%	0.09%	0.04%	0.96%	0.88%
Non-COVID NMR	0.95%	1.05%	1.69%	2.06%	2.28%	2.09%	2.26%	0.92%	4.29%	5.27%
CEMP	20.91%	6.23%	18.86%	34.66%	33.70%	2.12%	4.02%	4.04%	22.42%	16.72%
**Vaccinees** (J&J and mixed)	1135	47,320	56,233	92,702	119,607	125,399	128,648	132,141	119,607	132,141
**Pfizer vaccinees**										
1 or 2 doses	346,101	590,474	627,152	346,342	238,626	229,110	224,490	218,121	238,626	218,121
3+ doses	0	0	0	305,633	416,218	425,792	430,667	437,840	416,218	437,840
Total Pfizer vaccinees	346,101	590,474	627,152	651,975	654,844	654,902	655,157	655,961	654,844	655,961
COVID-19 deaths	14	44	160	334	430	48	121	128	968	1279
Non-COVID natural deaths	459	2160	2670	3278	3406	3114	3147	3643	11,514	21,877
COVID-MR	0.01%	0.01%	0.03%	0.05%	0.07%	0.01%	0.02%	0.02%	0.19%	0.39%
Non-COVID NMR	0.27%	0.46%	0.44%	0.51%	0.52%	0.48%	0.48%	0.56%	2.30%	6.67%
CEMP_Pfizer_	3.05%	2.04%	5.99%	10.19%	12.62%	1.54%	3.84%	3.51%	8.41%	5.85%
$RMR_{P,unvax}^{CEMP}$	0.146	0.327	0.317	0.294	0.375	0.727	0.957	0.869	0.375	0.350
HVBCF_P,unvax_	0.278	0.440	0.260	0.249	0.228	0.227	0.212	0.223	0.537	1.266
**Moderna vaccinees**										
1 or 2 doses	389,026	553,316	573,870	351,405	230,046	225,560	224,708	214,967	230,046	214,967
3+ doses	0	0	0	226,658	345,614	351,276	353,085	359,697	345,614	359,697
Total Moderna vaccinees	389,026	553,316	573,870	578,063	575,660	576,836	577,793	574,664	575,660	574,664
COVID-19 deaths	62	62	143	398	464	112	166	190	1066	1597
Non-COVID natural deaths	1474	4142	4530	4938	4865	4454	4546	5066	18,475	34,015
COVID-MR	0.03%	0.01%	0.03%	0.07%	0.08%	0.02%	0.03%	0.03%	0.22%	0.56%
Non-COVID NMR	0.76%	0.88%	0.80%	0.86%	0.84%	0.77%	0.79%	0.88%	3.83%	11.84%
CEMP_Moderna_	4.21%	1.50%	3.16%	8.04%	9.54%	2.51%	3.65%	3.75%	5.78%	4.69%
$RMR_{M,unvax}^{CEMP}$	0.201	0.240	0.167	0.232	0.283	1.186	0.909	0.927	0.258	0.281
HVBCF_M,unvax_	0.794	0.838	0.477	0.417	0.370	0.369	0.348	0.354	0.893	2.247
$RMR_{P,M}^{1or2}$	0.725	1.361	1.897	1.316	1.330	0.557	0.965	1.200	1.494	1.363
[95% CI]	[0.402, 1.307]	[0.922, 2.010]	[1.46, 2.464]	[1.211, 1.544]	[1.132, 1.563]	[0.342, 0.906]	[0.661, 1.408]	[0.833, 1.731]	[1.353, 1.651]	[1.247, 1.490]
$RMR_{P,M}^{3+}$				1.899	1.341	0.675	1.130	0.805	1.343	1.044
[95% CI]				[0.831, 4.340]	[1.012, 1.776]	[0.418, 1.091]	[0.829, 1.538]	[0.601, 1.078]	[1.033, 1.747]	[0.895, 1.219]

## Data Availability

The linked mortality and vaccination data on which this study relies was obtained under a data use agreement with the Regenstrief Institute at Indiana University and cannot be publicly shared.

## Supplementary Materials

The following supporting information can be downloaded at: https://www.mdpi.com/article/10.3390/vaccines14080717/s1.
