# Supplementary material for: Pfizer Versus Moderna COVID-19 Vaccine Effectiveness Against Mortality: Evidence from Linked Mortality and Vaccination Records in Indiana
Source: Vaccines (Basel). 2026 Aug 20;14(8):717. doi: 10.3390/vaccines14080717 (PMC13517372; doi:10.3390/vaccines14080717)

Supplementary Material for

Pfizer versus Moderna COVID-19 Vaccine Effectiveness against Mortality: Evidence from Linked Mortality and Vaccination Records in Indiana

Sadia Farzana
Northwestern University

Francesco Maria Rossi
Northwestern University

Qian (Eric) Luo
George Washington University

Jeff Whittle
Medical College of Wisconsin

Kevin McGurk
Medical College of Wisconsin

Benjamin W. Weston
Medical College of Wisconsin

Andy Ye Yuan
University of Florida

Ali Moghtaderi
George Washington University

Vladimir Atanasov
William & Mary, Mason School of Business

Bernard Black
Northwestern University, Pritzker School of Law and Kellogg School of Management

Draft July 2026

Northwestern University, Pritzker School of Law
Law and Economics Research Paper No. 26-xx

Northwestern University, Institute for Policy Research, Working Paper 26-xx

This Supplementary Material can be downloaded from SSRN at:
http://ssrn.com/abstract=6xxxxxx

The paper can be downloaded from SSRN at:
http://ssrn.com/abstract=6xxxxxx

Supplementary Material for

Pfizer versus Moderna COVID-19 Vaccine Effectiveness against Mortality: Evidence from Linked Mortality and Vaccination Records in Indiana

Sadia Farzana, Francesco Rossi, Qian (Eric) Luo, Jeffrey Whittle, Kevin McGurk, Benjamin Weston, Andy (Ye) Yuan, Ali Moghtaderi, Vladimir Atanasov, and Bernard Black

Abstract: This Supplementary Material provides additional results and methods details for Farzana et al. (2026), *Pfizer versus Moderna COVID-19 Vaccine Effectiveness against Mortality: Evidence from Linked Mortality and Vaccination Records in Indiana*

Contents

Section S1. Indiana Population Estimates 1
Section S2. Details on Counting Vaccinated Persons and Number of Vaccine Doses 1
Section S3. Adjusting for Undercounting of Vaccinated Decedents 4
A. Adjustment for IHIE Undercount of Vaccinated Decedents 4
B. Estimating Confidence Intervals for Different Numbers of Vaccine Doses 6
Section S4. Identifying COVID-19 Decedents from Cause of Death Fields 7
Section S5. Additional Methods Details 8
A. Vaccine Approval Dates 8
B. Choice of "Core" Study Period 8
C. Definitions of Terms, and Relations Between Them 9
D. Univariate Logistic Regression and Confidence Intervals 10
E. Confidence Intervals for HVB_CF 12
Section S6. Validating the CEMP Measure 12
A. Correlation Between Natural non-COVID Deaths and COVID-19 Deaths 12
B. Multivariate Regressions 13
1. Overall Vaccine Effectiveness 13
2. Comparison of Pfizer and Moderna 13
Section S7. Mixed Vaccine Dose Patterns 14
Section S8. Vaccination Timing 14
Section S9. Relative Pfizer vs. Moderna CEMP By Time Since Vaccination 15
Section S10. Defining the Immune-Compromised 15
Table S1. Comparison of COVID-19 deaths per text analysis, to ICD-10 codes 17
Table S2. Summary Statistics on Vaccination Status 18
Panel A. Sample (Excludes Immune-compromised persons) 18
Panel B. Immune-compromised persons 19
Table S3. Summary Statistics, Ages 15-59 20
Table S4. Healthy Vaccinee Bias Correction Factor (HVBCF). 21
Panel A. Ages 60+ 21
Panel B. Ages 15-59 22
Table S5. Relative Mortality Risk (Based on CEMP) 24
Panel A. Ages 60+ 24
Panel B. Ages 15-59 25
Table S6. Summary Statistics, Separately for Ages 60-79 and 80+ 26
Panel A. ages 60-79 26
Panel B. ages 80+ 27
Table S7. Multivariate Logit Results for *RMR* 28
Table S8. Multivariate Logit Results for *RMR*, Pfizer vs. Moderna 29
Table S9. Vaccine Approval Dates 31
Figure S1. Correlation between Natural Mortality in 2019 and COVID-19 Mortality in 2020 32
Figure S2. Correlation between Natural Mortality Rates in 2019 and COVID-19 Mortality Rates in 2020. 33
Figure S3. Indiana Natural Deaths in 2019 vs. Non-COVID Natural Deaths in 2020 34
Figure S4. Actual versus Predicted NC-NMR During Pandemic Period 35
Panel A. Actual versus Predicted Non-COVID Natural Mortality 35

Panel B. Measured and Predicted Non-COVID Natural Deaths, with Confidence Intervals.........35
Figure S5. Raw vs. Interpolated Deaths for Vaccinated Persons 36
Panel A. Ages 15-59 36
Panel B. Ages 60+.........36
Figure S6. Vaccination Rates for Adults by Age Group in Indiana 37
Panel A. Full Vaccination Rates 37
Panel B. At least Three Dose Vaccination Rates 37
Figure S7. RMR and HVB for ages 60+ with any number of doses 38
Panel A. HVBCF 38
Panel B. RMR and Pfizer/Moderna RMR Ratio 38
Figure S8. Healthy Vaccinee Bias Correction Factor for Ages 15-59 39
Panel A. Separate Results for Primary and Booster Vaccination 39
Panel B. Any number of doses 39
Figure S9. Remaining Mortality Risk for Pfizer and Moderna, ages 15-59.........40
Panel A. Separate Pfizer and Moderna RMRs 40
Panel B. Pfizer/Moderna RMR Ratio, age 15-59 40
Figure S10. Healthy Vaccinee Bias Correction Factor (Ages 60-79 and 80+) 41
Panel A. Ages 60-79 41
Panel B. Ages 80+ 41
Figure S11. Remaining Mortality Risk for Pfizer and Moderna (ages 60-79).........42
Panel A. Separate Pfizer and Moderna RMRs 42
Panel B. Pfizer/Moderna RMR Ratios 42
Figure S12. Remaining Mortality Risk for Pfizer and Moderna (ages 80+).........43
Panel A. Separate Pfizer and Moderna RMRs 43
Panel B. Pfizer/Moderna RMR Ratios 43
Figure S13. Healthy Vaccinee Bias Correction Factor (Ages 60+), by sex 44
Panel A. Male 44
Panel B. Female.........44
Figure S14. Remaining Mortality Risk for Pfizer and Moderna, ages 60+ by sex.........45
Panel A. Separate Pfizer and Moderna RMRs 45
Panel B. Pfizer/Moderna RMR Ratios 46
Figure S15. Monthly Vaccination Counts by Manufacturer.........47
Panel A. First Dose.........47
Panel B. Second Dose.........47
Panel C. Third Dose.........48
Figure S16. RMR Pfizer/Moderna ratios, univariate vs. multivariate regressions 49
Panel A. Age 60+.........49
Panel B. Age 15-59.........49
Figure S17. Mortality Relative to Vaccination Date for 1Q2021 Vaccinees.........50
Panel A. Ages 60+, any number of doses.........50
Panel B. Ages 15-59, any number of doses.........50
Figure S18. Sensitivity: Booster-Dose RMR for Age 60+ with Mixed Booster Dose 51

Section S1. Indiana Population Estimates

We downloaded mid-year, state-level population estimates from the U.S. Census Bureau, disaggregated by sex, year of age, race, and ethnicity.[1] We use the 2020 decennial Census to obtain population estimates as of July 1, 2020. The Census reports population counts for each single year of age from 0 to 84, with an aggregate category for individuals aged 85 and older. We aggregate the data to construct population counts by sex (male or female) and age (0–84 by year of age, and 85+). Total estimated population aged 15+ is 5,460,000 persons.

The number of vaccinees is measured directly from vaccination records. The number of unvaccinated people in age group a is derived as:

$$Unvax_a = Population_a - Vax_a$$

For older ages, most people are vaccinated, so a small percentage error in measuring population implies a larger percentage error in estimating the unvaccinated population, thus in measuring the COVID-19 mortality rate and the non-COVID-19 natural mortality rate (NCNMR) for unvaccinated persons.

Using remaining mortality risk (RMR) as the outcome avoids this issue; any error in measuring population will affect counts of both COVID-19 and non-COVID natural deaths. It will not affect the COVID-19 Excess Mortality Percentage (CEMP) or CEMP-based RMR. The accuracy of population estimates will affect the accuracy of estimates of the healthy vaccinee bias correction factor (HVBCF) for Pfizer and Moderna but will not affect relative Pfizer-versus-Moderna estimates for either HVBCF or RMR.

Section S2. Details on Counting Vaccinated Persons and Number of Vaccine Doses

We obtained data on vaccinated individuals in Indiana, including date and vaccine type of each vaccine dose, from the Indiana Health Insurance Exchange (IHIE). The original IHIE dataset, before cleaning, includes 10,626,687 vaccine doses for 4,126,277 distinct individuals. We remove:

(i) 780 duplicates, identified based on same IHIE identification number, same vaccination dates, and same vaccine manufacturers;
(ii) Persons aged < 15 at the date of first vaccination
(iii) Persons with first dose after Dec. 31, 2022.

Primary vaccination with the mRNA vaccines (from Moderna, mRNA1273; and Pfizer-BioNTech, BNT162b2) used two initial doses; the Johnson & Johnson (J&J) vaccine used one initial dose; the Novavax vaccine (approved mid-2022, minimally used in our sample) also used two initial doses. Standard U.S. two-dose timing was 28 days between doses for Moderna and 21 days for Pfizer. Mixing of vaccine types was uncommon (see the small table below).

We observe many cases where one person has an implausible number of vaccinations, often close together in time. Many of these appear to involve cases where one SID reflects the

[1] Source: https://www2.census.gov/programs-surveys/popest/. Population estimates are available for six race categories, with an indicator for Hispanic origin. The race categories are: White Alone, Black or African American Alone, American Indian or Alaska Native Alone, Asian Alone, Native Hawaiian and Other Pacific Islander Alone, Two or more races.

vaccinations of two separate people. We also see cases where two IHIE records have identical or very similar vaccination records and are probably duplicate records.

We adopt the following cleaning rules. We applied these rules before further limiting the sample, for this project, to only Pfizer and Moderna vaccinees.

1. We drop one vaccination date before 14 December 2020, as recorded in error. This person (see details below) provides an example of common errors:

   The vax date of 18 Jan 2020 is obviously incorrect. The correct date appears to be 18 January 2021, which would be 3 weeks after the Pfizer dose on 28 December 2020. A vaccine dose is also recorded on 18 January 2021.

   This record could be for one person, who was vaccinated on 23 or 28 December 2020 and wrongly recorded as vaccinated twice 5 days apart, who received a second dose on 18 January 2021 (also recorded twice, once with a wrong date), and then a booster dose in November 2021.

IHIE ID	Vaccine date	Type	Dose number	Note
390815	18-Jan-20	Pfizer	1	Incorrect vax date
390815	23-Dec-20	Pfizer	2	
390815	28-Dec-20	Pfizer	3	
390815	18-Jan-21	Pfizer	4	
390815	5-Nov-21	Pfizer	5	

2. We drop 201 J&J vaccinations that occurred before the Food and Drug Administration's (FDA) authorization of J&J on 26 February 2021.

3. We drop 88 Novavax vaccinations that occurred before the FDA's authorization of Novavax on 13 July 2022.

4. There is a small gap between the first two doses for many people, relative to the Pfizer minimum of 21 days and the Moderna minimum of 28 days. The number of second doses starts to climb for Pfizer shortly before a 21-day gap, and for Moderna shortly before a 28-day gap. The table below shows the gap between first and second doses in days. Cells with the minimum gap are in **boldface**.

First and second doses separated by 35 days or less

**Days between doses**	**Pfizer**	**Moderna-**	**J&J**	**Novavax**	**Pfizer-Moderna or reverse**	**J&J-P/M**
0					78	9
1	35	23	5		6	1
2	185	100	17		34	5
3	146	106	12		37	3
4	1363	855	169		246	50
5	1340	882	180		274	45
6	1440	680	146		256	56
7	2185	1119	208		431	96
8	1302	667	133		226	8
9	1134	509	110		220	44
10	1817	812	138		261	43
11	1161	494	79		162	346

**Days between doses**	**Pfizer**	**Moderna-**	**J&J**	**Novavax**	**Pfizer-Moderna or reverse**	**J&J-P/M**
12	1260	388	92		150	364
13	1039	391	56		180	368
14	1808	567	59		195	593
15	1158	396	52		149	322
16	1416	348	59		157	304
17	3899	424	47		128	369
18	4681	524	41	1	97	248
19	10,575	474	56		118	222
20	28,394	707	70	4	154	242
21	**1,197,534**	1606	60	48	**863**	295
22	204,584	759	49	15	232	229
23	119,523	1013	53	4	168	195
24	80,080	2341	45	3	163	182
25	65,273	3339	46		106	161
26	53,952	6671	40	1	116	194
27	46,603	17,194	31	3	133	234
28	121,772	**681,906**	47	11	**837**	1201
29	36,310	114,121	41	1	233	346
30	25340	69,267	41		166	252
Pfizer 0-17 days	22,688					
Moderna 1-24 days		16,185				
Mixed 1-17 days					3,112	

After reviewing this pattern, we adopt the following rules for the first two doses

(a) Drop the second dose if the gap is 0-17 days for Pfizer, J&J and mixed vaccine patterns;

(b) Drop the second dose if the gap is 0-24 days for Moderna.

5. *Third dose*. To count a dose as a third dose, we require it to be at least 120 days after the second dose and after the first date when the FDA allowed a third dose for the immunocompromised (Aug. 12, 2021) (this date restriction affects 1,336 people). Doses received earlier than this are dropped. The minimum allowed timing for a Pfizer or Moderna booster dose was 6 months, except for the immunocompromised. The minimum allowed timing for a second dose after a first J&J dose was 2 months, but that rule should not be relevant for the third dose.

6. Third dose for immunocompromised. We keep the third dose for 43,358 people vaccinated between August 12, 2021 and FDA approval of the third dose for non-immunocompromised persons on Sept. 25, 2021, but treat receipt of a third dose during this period as evidence of immunocompromised status, and remove immunocompromised persons from the sample.

The tendency for IHIE to sometimes combine two vaccinees into one record creates a small risk of bias, of uncertain sign. If two actual vaccinees are combined, we might either under- or over-estimate vaccinee mortality rates for vaccinated persons. If we underestimate vaccinee mortality rates, the bias will be similar to the bias, discussed in the next section, from undercounting vaccinated decedents during August-October 2021**.** If we overestimate vaccinee mortality rates, the bias will have the opposite sign. The percentage of combined vaccination

records appears to be small, around 1-2%, so any bias should also be small. We also have no reason to expect any bias to affect Pfizer-versus-Moderna comparisons.

Immunocompromised persons. In counting the vaccinated (and thus the unvaccinated population) and measuring vaccine effectiveness, we excluded immunocompromised decedents. We define these people as: (i) decedents whose death records indicate a solid organ transplant; (ii) decedents whose death records state that they were immunocompromised; and (iii) persons who received a third vaccine dose on or before September 24, 2021, the first date when a third dose was authorized for the non-immunocompromised. The standard CDC recommendation for full vaccination included a third "primary" dose; for these persons, the fourth dose was the first dose considered to be a booster dose.

Counting vaccinated persons and which deaths are considered to be of vaccinated decedents. We conduct analysis for monthly periods, which we then roll up to calendar quarters. When assessing whether decedents were vaccinated, and with how many doses, we first shifted vaccination dates forward by 30 days. We then treated people vaccinated before the 15th of a month as vaccinated at the beginning of that month, whereas those vaccinated on or after the 15th are counted as vaccinated as of the beginning of the following month.

For example, a person vaccinated on February 12 would be treated as vaccinated as of March 14; we then shift this date back to March 1 in deciding whether a death occurred while vaccinated. Thus, a death for this person would be considered unvaccinated during February, and vaccinated in March or later.

This approach will lead to counting a small number of vaccinated decedents as if unvaccinated even though death occurred more than 30 days after vaccination, and a small number of unvaccinated decedents as if vaccinated even though death occured less than 30 days after vaccination. However, we have no reason to expect these misclassifications to cause bias when comparing Pfizer to Moderna vaccinees.

We use the monthly counts of vaccinated persons to calculate monthly mortality rates. We report quarterly rates based on the average of the population at the beginning and end of the quarter.

Section S3. Adjusting for Undercounting of Vaccinated Decedents

We define natural deaths as those with manner of death recorded as natural on the death certificate; the vast majority of decedents (91.39%) have natural manner of death. We exclude the remaining manner-of-death categories (accident, homicide, and suicide; together 8.04% of all deaths), and pending and undetermined (together, 0.57%).

We obtain IHIE data on vaccinees, number of vaccine doses, which vaccinees have died, and cause of death for the decedents. We clean the number of vaccine doses, as described above. We also have Indiana Vital Statistics (VS) records on all decedents.

A. Adjustment for IHIE Undercount of Vaccinated Decedents

We observe failure of IHIE to fully match VS death records to vaccination records in August-October 2021. Figure S5 shows vaccinated decedents by month (from all natural causes) in the IHIE linked vaccination-mortality records, separately for ages 15-59 and 60+. We expect the number of vaccinated decedents to rise rapidly during 2021, once vaccines are available, as more people become vaccinated, and thus can potentially die while vaccinated. This is observed in general, but with a visually obvious drop in August-October 2021 in the number of vaccinated

decedents. We asked IHIE personnel about the source of this dip. They recognized the issue but had no solution to offer. The undercount of vaccinated decedents implies a corresponding overcount of unvaccinated decedents during 3Q2021 and 4Q2021. This can produce downward bias in RMR^{CEMP} estimates.

We address the undercount, and thus the bias in RMR, as follows. First, we interpolate from July 2021 to November 2021, to obtain adjusted counts of vaccinated decedents. We then increase the monthly numbers of *adjusted* vaccinated deaths so that they follow the interpolation line and correspondingly decrease the numbers of *adjusted* unvaccinated deaths.

We assume that the missing deaths of vaccinated persons reflect random matching error, and are unrelated to whether the death was due to COVID or not or which vaccine someone received. With this assumption, which we view as reasonable, the adjustment to vaccinated deaths will not affect CEMP for all vaccinated persons ($CEMP_{vax}$). Moreover, we can conduct a single adjustment for both Pfizer and Moderna vaccinees.

To compute *adjusted* $CEMP_{unvax}$, we proceed as follows. We first used the measured $CEMP_{vax}$ to attribute the extra interpolated deaths among vaccinated persons to COVID versus other natural causes. We then remove the same numbers of COVID and natural, non-COVID deaths from unvaccinated persons and recompute $CEMP_{unvax}$.

To use a numerical example, assume that interpolation increases the number of vaccinated deaths, for a given age group in a given quarter, by 100, and that $CEMP_{vax}$ is 25%. Then, of the 100 additional vaccinated deaths, 20 should be COVID and 80 should be non-COVID (20/80 = 0.25). We therefore remove 20 COVID deaths and 80 non-COVID deaths from the unvaccinated population, and compute *adjusted* $CEMP_{unvax}$. This will be *higher* than unadjusted raw $CEMP_{unvax}$ because the removed deaths are disproportionately non-COVID deaths. This, in turn, will *reduce* RMR (calculated as $\frac{CEMP_{vax}}{CEMP_{unvax}}$), illustrating that the unadjusted RMR was biased upwards.

We compute adjusted $CEMP_{unvax,a,t}$ separately by age group, and quarter, for 3Q and 4Q 2021, for the age groups used in the text (15-59 and 60+).

The table below provides the steps in our calculation of adjusted counts of vaccinated decedents with 1 or 2 vaccine doses and unvaccinated decedents. We compute missing vaccinated decedents by month, and roll up to quarter. We did not adjust counts for 3-dose recipients; booster doses were approved only at the very end of the period in which we observed undercounts (taking into account the 30-day delay we impose between vaccination date and when we consider vaccination effective against death).

Table Interp-1: Original and Interpolated Counts of COVID and other Natural Deaths

Table shows original IHIE counts of vaccinated ("vax") decedents from natural causes, who received 1+ vaccine doses, and interpolated counts for 3Q and 4Q2021. Deaths are counted from 30 days after receipt of first vaccine dose. Interpolated amounts are rounded to 2 decimal places. CEMP is shown as a fraction rather than a percentage. Sample excludes immunocompromised persons.

**Age**	**Formulas**	**15-59**	**15-59**	**60+**	**60+**
Quarter		**3Q2021**	**4Q2021**	**3Q2021**	**4Q2021**
Observed vax deaths	(1)	562	949	6101	9079
Interpolated vax deaths	(2)	728.25	980.25	7991.25	9632.25
Observed vax COVID deaths	(3)	28	65	255	759
Observed vax non-COVID deaths	(4)	534	884	5846	8320
Observed $CEMP_{vax}$	$(5) = \frac{(3)}{(4)}$	0.052	0.074	0.044	0.091
Interpolated COVID deaths for vaccinated	$(6) = (2) * [\frac{(5)}{1+(5)}]$	36.28	67.14	334.01	805.25
Interpolated non-COVID deaths for vaccinated	(7) = (2) – (6)	691.97	913.11	7,657.24	8,827
Extra observed COVID deaths for unvaccinated	(8) = (6)-(3)	8.28	2.14	79.01	46.25
Extra observed non-COVID deaths for unvaccinated	(9) = (7) – (4)	157.97	29.11	1811.24	507.00
Observed unvax COVID deaths	(10)	446	676	1100	1865
Adjusted unvax COVID deaths	(11) = (10) – (8)	437.72	673.86	1020.99	1818.75
Observed unvax non-COVID deaths	(12)	1559	1343	7224	5754
Adjusted unvax non-COVID deaths	(13) = (12) – (9)	1401.03	1313.89	5412.76	5247.00
Observed $RMR_{vax,unvax}$		0.1833	0.1461	0.2865	0.2815
Adjusted $RMR_{vax,unvax}$		0.1678	0.1434	0.2312	0.2632

B. Estimating Confidence Intervals for Different Numbers of Vaccine Doses

To estimate confidence intervals for $RMR_{x,unvax}^{CEMP,adj}$, we apply weighted logistic regression within each broad age group. We calculate weights as the ratio of interpolated to observed counts for both COVID and non-COVID deaths. The weights depend on the numbers of doses x. The weight construction is shown below. We use the same weights for both Pfizer and Moderna vaccinees.

Table Interp-2: Original and Interpolated Counts: 1- and 2-Dose Vaccinees

Table shows original IHIE counts of decedents from natural causes, who received 1 (panel A) or 2 (Panel B) vaccine doses, and interpolated counts for 3Q and 4Q2021. Deaths are counted from 30 days after receipt of first vaccine dose. Interpolated amounts are rounded to 2 decimal places. CEMP is shown as a fraction rather than a percentage. Sample excludes immunocompromised persons.

Panel A. 1 Dose

**Age**	**Formulas**	**15-59**	**15-59**	**60+**	**60+**
Quarter		3Q2021	4Q2021	3Q2021	4Q2021
Observed deaths, 1 dose	(1)	124	228	532	865
Interpolated deaths, 1 dose	(2)	171.75	237.75	721.5	882.5
Observed COVID deaths, 1 dose	(3)	10	27	36	107
Observed non-COVID deaths, 1 dose	(4)	114	201	496	758
Observed $CEMP_1$	$(5) = \frac{(3)}{(4)}$	0.088	0.134	0.073	0.141
Interpolated COVID deaths for vaccinated	$(6) = (2) * [\frac{(5)}{1+(5)}]$	13.851	28.155	48.823	109.165
Interpolated non-COVID deaths for vaccinated	(7) = (2) – (6)	157.899	209.595	672.677	773.335

Panel B. 2 Doses

**Age**	**Formulas**	**15-59**	**15-59**	**60+**	**60+**
Quarter		**3Q2021**	**4Q2021**	**3Q2021**	**4Q2021**
Observed deaths, 2 doses	(1)	438	700	5,569	7,302
Interpolated deaths, 2 doses	(2)	553.50	718.50	7,135.50	7,709.50
Observed COVID deaths, 2 doses	(3)	18	38	219	621
Observed non-COVID deaths, 2 doses	(4)	420	662	5350	6681
Observed $CEMP_2$	$(5) = \frac{(3)}{(4)}$	0.429	0.057	0.041	0.093
Interpolated COVID deaths for vaccinated	$(6) = (2) * [\frac{(5)}{1+(5)}]$	22.747	39.004	280.602	655.656
Interpolated non-COVID deaths for vaccinated	(7) = (2) – (6)	530.753	679.496	6,854.898	7,053.844

The differences between raw and adjusted RMR are small but visible in graphs (not shown) for 2Q2021, and nearly invisible for 3Q2021. Our reported results use adjusted $RMR_{vax,unvax}$ for these two quarters.

Section S4. Identifying COVID-19 Decedents from Cause of Death Fields

The death certificates that we rely on include text fields for primary cause of death, other conditions in the causal chain, and other significant conditions. We conduct text analysis of these fields to determine which deaths are likely to be caused by COVID-19. Table S1 compares the COVID-19 counts we determine using text analysis to those from the ICD-10 codes included in death records, which are generated by personnel at the National Center for Health Statistics (NCHS) based on the text fields. Our text analysis algorithm was developed and substantially completed before we had access to the ICD-10 codes. Our goal was to develop a reasonable method for estimating COVID-19 deaths, based on the text fields in death certificates. The principal decision-makers for this approach were Drs. John Meurer and Jeff Whittle. The total counts of COVID-19 deaths are very similar using both approaches.

The detailed coding we used to identify deaths as due to or probably due to COVID-19 is available from the authors on request. In brief, we counted deaths as COVID-19 deaths when:

(i) COVID-19 or variants (such as SARS-2, SARS-COV-2, coronavirus) was listed as the principal cause of death; or

(ii) COVID-19 or variants were listed in the causal chain and the principal cause of death was likely to be caused by COVID-19 (for example, pneumonia, respiratory failure, hypoxia); or

(iii) COVID-19 or variants were listed in the causal chain and the principal cause of death was a known potential outcome of COVID-19 infection (for example, heart attack, stroke, sepsis) or involved underlying disease that was plausibly exacerbated by COVID-19 infection (for example, heart failure, COPD, dementia); or

(iv) COVID-19 was included in the "other significant conditions" field, but not in the causal chain fields, and (a) the principal cause of death was likely to be caused by COVID (group (ii) above); and (b) there was no entry in the causal chain fields indicating another likely cause of death.

We view reliability as high for categories (i) and (ii), and moderate for categories (iii) and (iv). The false positives (COVID-19 as principal cause of death based on ICD-10 codes, but not on our text-based coding) involve deaths for which the text fields do not fit in any of these four categories.

Section S5. Additional Methods Details

A. Vaccine Approval Dates

COVID-19 vaccines became available in the U.S. in early 2021, but initially with limited supply, generally the elderly, other persons at high risk for severe COVID-19, healthcare workers, and other persons in high-exposure occupations. Availability expanded greatly in April 2021 and vaccines became available to all who wanted them by May 2021.

Table S9 summarizes vaccine approval dates. Boosters were first authorized by the Centers for Disease Control and Prevention (CDC) on September 24, 2021, initially for ages 60+ and healthcare workers and others in high-risk occupations. The CDC authorized boosters for all adults on November 19, 2021, but recommended them only for ages 50+.[2] On November 29, 2021, the CDC recommended boosters for all adults.[3]

The first Moderna booster was approved four weeks after the Pfizer booster, on October 21, 2021. This led to some people who received Moderna for primary vaccination switching to Pfizer for the first booster dose. Switching was allowed for the booster, but not for primary vaccination.

B. Choice of "Core" Study Period

We study the period from 1Q2021-4Q2022, but focus in the text on a "core" period, from 2Q2021-1Q2022. We excluded 1Q2021 for several reasons. The primary reason was limited vaccine availability. In 1Q2021, the mRNA vaccines were in limited supply and were being rolled out with complex priority rules. Priority was given to health care workers, other first responders, older persons, and other persons at higher risk for severe COVID-19. Local governments set their

[2] CDC Press Release (Nov. 19, 2021); Mandavilli (2021).

[3] CDC Press Release (Nov. 29, 2021).

own local priority rules; for example, allowing vaccination above age 80 through date X, then above age 78 through date Y, etc.

The vaccination priority rules were generally dropped during April 2021. Thus, 2Q2021 is the first quarter with widespread first-dose vaccine availability.

A second reason for starting the core period with 2Q2021 involved the two-dose primary vaccination schedule, with doses a minimum of 21 days apart for Pfizer and 28 days apart for Moderna. Coupled with the 30-day delay period we imposed between vaccination date and when we considered a person to be vaccinated against death, two two-dose schedule meant that counts of effectively-vaccinated-against-death decedents were low in 1Q2021, rose rapidly during the quarter, and disproportionately involved persons who had received only one dose, and disproportionately involved people age 80+. Overall, of 76 deaths over 1Q2021 among Pfizer and Moderna vaccinees aged 60+ (text Table 1), 52 involved persons aged 80+, and only 11 involved death at least 30 days after receiving a second dose.

Based on a combination of these reasons, we viewed results for 1Q2021 as potentially unrepresentative of the overall comparison between Pfizer and Moderna. This was a judgment call. Results for the core period would not meaningfully change if we added 1Q2021 to this period.

We ended the core period with 1Q2022, based on much lower mortality rates in 2Q2022 and evidence that RMR versus the unvaccinated was substantial through 1Q2022, but small and statistically insignificant after this, as we reported previously (Farzana et al., 2025). After the large Omicron wave in 1Q2022, a large proportion of vaccinees were also already infected. Thus, the Pfizer-vs-Moderna comparison had become, for many people, a comparison of two types of hybrid immunity: Pfizer-plus-prior-infection vs. Moderna-plus-prior-infection. We viewed this as a different research question, for which the protection provided by prior infection would likely mute any Pfizer-vs-Moderna difference. We in fact found no evidence for a meaningful Moderna advantage from 2Q2021 on (text Figure 2).

C. Definitions of Terms, and Relations Between Them

The formulas in this section generalize those in the text to any comparison of people with x versus y vaccine doses (y = 0 for the unvaccinated), of vaccine type v. We add a CEMP superscript on RMR (RMR^{CEMP}) to distinguish CEMP-based RMR from raw RMR, based solely on COVID-19 mortality rates (RMR^{raw}). We define the COVID-19 Excess Mortality Percentage (CEMP), for persons with x vaccine doses, vaccine effectiveness for x doses versus a base number of y doses ($VE_{x,y}$), and relative mortality risk after vaccination with x versus y doses ($RMR_{x,y}$) in time period *t*, within age group *a*, for vaccine type *v* (meaningful only for a positive number of doses), as:

$$CEMP_{xv} = 100 * \frac{COVID\ deaths_{xv}}{non\text{-}COVID\ natural\ deaths_{xv}} \quad (1a)$$

$$RMR^{CEMP}_{xv,unvax} = \frac{CEMP_{xv}}{CEMP_{unvax}} \quad (1b)$$

$$VE^{CEMP}_{xv,unvax} = 1 - RMR^{CEMP}_{xv,unvax} \quad (1c)$$

By using non-COVID natural deaths in the CEMP denominator, we treat the non-COVID natural mortality rate as a proxy for the overall health of a given group, which can control for

selection effects, including who gets vaccinated, when, with which vaccine, and with how many doses. Because the sample is decedents, these estimates are, in effect, mortality-weighted. Thus, results for a broad age group give primary weight to older persons within that group.

We count a person as vaccinated with x doses beginning 30 days after the x'th dose.

$RMR^{CEMP}_{xv,unvax}$ can be obtained by comparing mortality rates for two groups, as in eqn. (1b), or as an odds ratio from logistic regression for a population containing both groups.

CEMP, VE, and RMR involve ratios, so will be undefined if the denominator is zero.

It is also useful to define a measure of healthy vaccinee bias (HVB), for persons with x vaccine doses relative to a base number of y doses, in time period *t* for age group *a and vaccine type v*. Let Non-COVID-NMR (or NCNMR) be the non-COVID natural mortality rate:

$$NCNMR_{x,v,a,t} = \frac{non - COVID\ natural\ deaths_{x,v,a,t}}{Pop_{x,v,a,t}}$$

We measure the denominator for quarter t as the average of the population at the beginning of quarter t and quarter t+1.

We can then define a healthy vaccinee bias correction factor:

$$HVB\_CF_{x,y,v,a,t} = \frac{NCNMR_{x,v,a,t}}{NCNMR_{y,v,a,t}}$$

Healthy vaccinee bias, in which vaccinated people are healthier than the unvaccinated, and people with more vaccine doses are healthier than people with fewer doses, and thus have lower NCNMR rates, implies HVB_CF values less than one. The central assumption for this measure of healthy vaccinee bias is that COVID-19 vaccination does not affect non-COVID natural mortality.

Now also define an RMR^{raw} measure, which is based on raw COVID-19 mortality rates (COVIDMR), rather than on CEMP, and thus does not use NCNMR rates to address healthy vaccinee bias.

$$RMR^{raw}_{x,y,v,a,t} = \frac{COVIDMR_{x,v,a,t}}{COVIDMR_{y,v,a,t}}$$

There is an interesting relationship between HVB_CF, RMR^{CEMP} and RMR^{raw}. Multiplying $RMR^{CEMP}_{x,y,v,a,t}$ by $HVBCF_{x,y}$ we get:

$$RMR_{x,y,v,a,t} * HVB\_CF_{x,y,a,t} = \left(\frac{\frac{COVID\ deaths_{x,v,a,t}}{non - COVID\ natural\ deaths_{x,v,a,t}}}{\frac{COVID\ deaths_{y,v,a,t}}{non - COVID\ natural\ deaths_{y,v,a,t}}} \right) \left(\frac{\frac{non - COVID\ natural\ deaths_{x,v,a,t}}{Pop_{x,v,a,t}}}{\frac{non - COVID\ natural\ deaths_{y,v,a,t}}{Pop_{y,v,a,t}}} \right)$$

Simplifying:

$$RMR^{CEMP}_{x,y,v,a,t} * HVB\_CF_{x,y,v,a,t} = \frac{\frac{COVID\ deaths_{x,v,a,t}}{Pop_{x,v,a,t}}}{\frac{COVID\ deaths_{y,v,a,t}}{Pop_{y,v,a,t}}} = RMR^{raw}_{x,y,v,a,t},$$

Rearranging:

$$RMR^{CEMP}_{x,y,v,a,t} = \frac{RMR^{raw}_{x,y,a,t}}{HVB\_CF_{x,y,a,t}}$$

Thus, for example, if $RMR^{raw} = 0.10$ (90% VE^{raw}) and HVB_CF is 0.5 (vaccinees have half the background mortality risk of the unvaccinated), then $RMR^{CEMP} = \frac{0.1}{0.5} = 0.20$.

D. Univariate Logistic Regression and Confidence Intervals

CEMP represents the odds, for a sample consisting of natural cause decedents, of dying from COVID-19 versus dying from other natural causes, converted to a percentage.

$$\text{CEMP} = 100 * \frac{\sum COV_i^{mort}=1}{\sum COV_i^{mort}=0}$$

Here COV_i^{mort} equals 1 for COVID-19 decedents, 0 for decedents from other natural causes.

This means that CEMP can be measured either directly, as in eqn. (1a), or through logistic regression. The logistic regression lets us obtain confidence intervals around CEMP point estimates. We run separate logistic regressions by calendar quarter, age group, and vaccine type using individual-level death records from Indiana Vital Statistics. The sample is limited to decedents from natural causes, excluding immune-compromised persons. The outcome variable is an indicator equal to one for COVID-19 death; zero if it was a non-COVID natural death. The main regressor of interest is vaccination status at time of death, represented by mutually exclusive indicator variables for each dose group (I_g). Unvaccinated persons are persons receiving three Pfizer doses the omitted reference category.

For each quarter *q,* age bin *a*, and vaccine type *v*, we estimate the following logistic regression:

$$\log\left(\frac{\Pr[COVID_i = 1 \mid dose_i = g]}{\Pr[COVID_i = 0 \mid dose_i = g]}\right) = \alpha + \beta_g * I_g + \varepsilon_i$$

for each vaccination group $g \neq 0$,, where $dose_i = g$ indicates individual *i*'s vaccination status, and group $g = 0$ (unvaccinated) is the reference category. Here:

- The left-hand side is the log-odds of a COVID death relative to a non-COVID natural death.
- β_g is the log of the odds ratio for group g relative to the unvaccinated group.
- Exponentiating this coefficient gives the odds ratio:

$$RMR^{CEMP}_{g,0} = \exp(\beta_g)$$

In our context, this odds ratio corresponds to RMR defined as the ratio of the COVID-to-non-COVID death ratio (i.e., CEMP) in group g relative to the same ratio in the unvaccinated group:

$$RMR_{g,0}^{CEMP} = \frac{CEMP_g}{CEMP_0} = \frac{\frac{COVID\ deaths_g}{nonCOVID\ deaths_g}}{\frac{COVID\ deaths_0}{nonCOVID\ deaths_0}}$$

This equivalence between the exponentiated regression coefficient and the algebraic expression for RMR arises because the regression is estimated only among decedents, and the model compares the odds of COVID versus non-COVID natural death within each vaccination group. We verify that the numerical values for CEMP and RMR^{CEMP}, obtained from the logistic regression above, exactly match values computed algebraically using death counts.

Confidence Intervals for RMR^{CEMP}. To construct 95% confidence intervals for RMR^{CEMP}, we exponentiate the lower and upper bounds of the confidence interval for the log-odds coefficient $\widehat{\beta}_g$, using robust standard errors.

$$CI_{RMR_g} = [\exp\left(\hat{\beta}_g - 1.96 * SE(\hat{\beta}_g)\right), \exp\left(\hat{\beta}_g + 1.96 * SE(\hat{\beta}_g)\right)]$$

The confidence interval is asymmetric around the RMR point estimate because the exponential function is nonlinear.

E. Confidence Intervals for HVB_CF

To obtain 95% confidence intervals for HVB_CF for persons with x versus y vaccine doses, we use the log-transformation method, and use the delta method for standard errors (Kirkwood and Sterne, 2003, p. 253):

$$SE(\log(HVB\_CF_{x,y,v,a,t})) = \sqrt{(\frac{1}{D_{xvat}} + \frac{1}{D_{yvat}})}$$

where $D_{x,v,a,t}$ and $D_{y,v,a,t}$ are the number of natural non-COVID deaths among persons with x and y vaccine doses, in age group a and time period t, with vaccine type v. To obtain 95% confidence intervals, we use the following formula:

$$CI_{HVB_{x,y,v,a,t}} = \left[\frac{HVB\_CF_{x,y,v,a,t}}{M}, HVB\_CF_{x,y,v,a,t} \times M\right], where\ M = \exp\left[1.96 * SE(log(HVB\_CF_{x,y,a,t}))\right]$$

Section S6. Validating the CEMP Measure

We seek to validate the CEMP measure, as reasonably but imperfectly controlling for HVB, in several ways. We summarize our validation steps in the text and provide discussion here.

A. Correlation Between Natural non-COVID Deaths and COVID-19 Deaths

CEMP uses NCNMR to proxy for overall health of a given group, and thus the likelihood of mortality if not vaccinated. Figure S1 compares natural deaths (counts) in Indiana over April–December 2019 (pre-COVID) to COVID-19 deaths over April–December 2020 for groups defined by age (15–39, 40–49, 50–59, 60–69, 70–79, 80–89, 90+), gender, race/ethnicity, and zip-SES quintile. Using 2019 natural mortality (rather than 2020 non-COVID natural mortality) to predict 2020 COVID mortality avoids the mechanical correlation which could arise if COVID deaths are undercounted or concurrent or prior COVID infection leads to higher non-COVID natural mortality. The Pearson correlation coefficient is 0.963.

Figure S2 is similar to Figure S1 but compares natural mortality *rates* (instead of counts) in April–December 2019 (pre-COVID) to COVID-19 mortality rates over April–December 2020. The Pearson correlation coefficient is only slightly lower, at 0.948.

In Figure S3, we report evidence for Indiana on the correlation between natural deaths in 2019 (pre-COVID period) and measured non-COVID natural deaths in 2020. The Pearson correlation coefficient is 0.999.

B. Multivariate Regressions

1. Overall Vaccine Effectiveness

The correlation evidence in Part A supports use of NCNMR non-COVID natural mortality as a reasonable proxy for baseline susceptibility of a group of persons to COVID-19 mortality. This evidence supports the central premise for our use of the CEMP measure: groups with higher underlying natural mortality also faced higher COVID-19 mortality risk before vaccination.

We sought to go beyond group-level correlations, in assessing the usefulness of the CEMP measure, as follows. We used the data available in death certificates to estimate a multivariate logistic regression model that controls for individual-level covariates. We estimate the following regressions separately for each calendar quarter and age group.

$$\log\left(\frac{\Pr[COVID_i=1 \mid dose_i=g]}{\Pr[COVID_i=0 \mid dose_i=g]}\right) = \alpha + \beta_g * I_g + f(age_i) + \boldsymbol{\gamma}'\boldsymbol{X_i} + \varepsilon_i \quad (1)$$

Here $f(age_i)$ is a quadratic in age, and $\boldsymbol{X_i}$ includes categorical indicators for sex, race, education, marital status, and quintiles of the Graham Social Deprivation Index, measured at zip-code level, as a measure of zip-SES. As in the univariate specification, the exponentiated coefficients on the dose indicators yield estimates of *RMR* by vaccination status, and we construct CIs by exponentiating the log-odds confidence bounds.

CEMP adjusts for background health by using non-COVID natural mortality in the CEMP denominator, while the logistic model adjusts directly for observed demographic and socioeconomic covariates. Table S7 compares CEMP-based RMR estimates with RMR estimates from multivariate logistic regressions in the broader Indiana vaccine-effectiveness analysis. The two approaches produce very similar estimates. Each of the CEMP-based point estimates are within the 95% CI from the multivariate regression approach, and vice versa.

2. Comparison of Pfizer and Moderna

We conduct a similar multivariate comparison of Pfizer to Moderna. We estimate equation (1), first comparing Pfizer vaccinees to the unvaccinated, and then comparing Moderna vaccinees to the unvaccinated, and obtain separate RMR estimates for each vaccine type. Table S8 reports these results. For ages 15-59, we report results for primary vaccination (1 or 2 doses) but not separate results for 1 dose and 2 doses; the combination of few 1-dose recipients and low mortality makes the 1-dose results not meaningful.

The final columns of this table compare the Pfizer/Moderna RMR ratio from the univariate CEMP approach with the corresponding ratio from multivariate logistic regression. To compute the multivariate ratio, we limit the sample to decedents who received a given number of doses of either Pfizer or Moderna. Equation (1) becomes:

$$\log\left(\frac{\Pr[COVID_i=1\,|dose_i=g;v=P]}{\Pr[COVID_i=0\,|dose_i=g;v=M]}\right) = \alpha + \beta_g * I_g + f(age_i) + \boldsymbol{\gamma}'\boldsymbol{X_i} + \varepsilon_i \quad (2)$$

For booster dose recipients over 2Q-4Q2022, this regression will not converge due to the small number of vaccinated decedents. These cells are shown as "n.a." in Table S8.

Figure S16 summarizes the Pfizer/Moderna ratios graphically, comparing the CEMP-based and multivariate estimates by quarter, age group, and dose group. The two approaches yield very similar results.

We note that the multivariate regression approach is limited to covariates available in death certificates. We do not observe individual health history, or other potentially important factors affecting COVID-19 mortality risk including prior infection, exposure to the SARS-COV-2 virus, income, healthcare access, or health behaviors. It does, however, provide evidence that CEMP does a sufficiently good job as a proxy for background health and thus COVID-19 mortality risk, so that the additional covariates available from death certificates do not materially affect our estimates.

Section S7. Mixed Vaccine Dose Patterns

In our main analysis, we excluded persons who received mixed vaccine types. In this section, we assess sensitivity to that exclusion. For primary vaccination, mixing vaccine types was not permitted; a small amount occurred in practice. A very small number of mixed-dose recipients appear in the data (5,046 persons out of 1,750 mRNA recipients; 0.29%). So mixing of vaccine types is not an important concern for primary vaccination. For booster dose, mixing was allowed but remained relatively uncommon; only 8.7% of mRNA vaccine recipients mixed vaccine types between the first two doses and a booster dose. See counts in Table S2.

We carried out a sensitivity analysis for booster recipients aged 60+, in which we included persons with M-M-P and P-P-M vaccine patterns in the sample, treated M-M-P as equivalent to receiving only Moderna (M-M-M) and treated P-P-M as equivalent to receiving only Pfizer (P-P-P). In Figure S18, we compare booster-dose RMR estimates allowing for mixed booster-versus-primary vaccine type to the estimates in the text, which exclude mixed-vaccine-type recipients. The left panel compares RMR for persons three Pfizer doses (same as text Figure 2, Panel A), to RMR for persons receiving primary Pfizer vaccination plus Moderna booster. The right panel compares RMR for persons receiving three Moderna doses (same as text Figure 2, Panel A), to

RMR for persons receiving primary Moderna vaccination plus Pfizer booster. Vertical bars show 95% confidence intervals. A horizontal dashed line at 1.0 indicates no reduction in mortality risk relative to unvaccinated persons. Vertical dashed lines indicate start and end of core vaccine-available, high-mortality period 2Q2021–1Q2022.

Including these mixed-vaccine persons in the sample has almost no effect on RMR versus the unvaccinated, for either vaccine (Figure S18, Panel A). Unsurprisingly, then, including these persons has almost no effect on the Pfizer/Moderna ratio (Panel B).

Section S8. Vaccination Timing

The large differences that we find between Pfizer and Moderna are surprising. In the U.S., especially during the period when most primary vaccination took place, there was no apparent difference in effectiveness between the two vaccines, no public discussion of difference, and thus no reason for anyone to prefer one vaccine over the other. Why, then, do we find large differences in HVB?

We investigated whether differences in vaccine rollout timing could help to explain differences in HVB. In Figure S15, we plot the monthly number of vaccine doses, by vaccine type, separately for ages 60+ and 15-59, and separately for first, second, and third doses. For the first and second doses, there are only small differences in relative timing of vaccine uptake. For the booster dose, there is more earlier uptake of Pfizer, but this reflects only that the Pfizer booster was approved about a month earlier than the Moderna booster (see Table S9 for exact dates).

Section S9. Relative Pfizer vs. Moderna CEMP By Time Since Vaccination

We sought to assess whether the relative protection from Pfizer versus Moderna varied with time since initial vaccination. A challenge for this analysis is that initial vaccination rates varied greatly over time (Figure S15), and COVID-19 mortality rates varied greatly over the pandemic period (Figure S4). We address this issue by studying only persons who received their first vaccine dose during 1Q2021, which was the peak vaccination period for ages 60+.

A second challenge is that the minimum time between first and second dose was 3 weeks for Pfizer and 4 weeks for Moderna, but actual time between first and second dose varied (see Part II). We also expect that much of the protective effect of vaccination starts with the first dose and the exact timing of the second dose was not very important. To put Pfizer and Moderna vaccinees on the same calendar scale, we measure mortality from the date of the first dose.

Figure S17 provides additional detail underlying Figure 4 in the main text. Panel A shows, for persons aged 60+ who received their first vaccine dose in 1Q2021, the COVID-19 mortality and non-COVID natural mortality rates used to construct the CEMP series shown in Figure 4. Moderna vaccinees generally have higher non-COVID natural mortality than Pfizer vaccinees, indicating differences in underlying mortality risk between the two groups. This reinforces the need for the CEMP approach: direct comparisons of COVID-19 mortality rates can mix vaccine-related differences with baseline mortality differences. Adjusting for these differences yields the CEMP pattern discussed in Section 3.7 and shown in Figure 4.

Panel B presents the corresponding COVID-19 mortality, non-COVID natural mortality, and CEMP series for persons aged 15–59. These series are substantially noisier because deaths are much less

frequent in this younger population. The CEMP series nonetheless shows some evidence of higher Pfizer CEMP during the later part of follow-up.

Section S10. Defining the Immune-Compromised

In the results in the text, we exclude persons known to be immune-compromised, defined as: (i) decedents whose death records indicate a solid organ transplant; (ii) decedents whose death records state that they were immune-compromised; and (iii) persons who received a third vaccine dose on or before September 24, 2021, the first date when a third dose was authorized for the non-immune-compromised. The standard CDC recommendation for full vaccination included a third "primary" dose; for these persons, the fourth dose was the first dose considered to be a booster dose. The booster studies by Arbel et al. (2021) and Bar-On et al. (2021) also exclude early third-dose recipients.

Table S2, Panel B, provides counts for the immune-compromised. These persons cannot be readily combined with the main sample of non-immune compromised persons because they received a third dose at different times. Many of them received a third dose before a booster was authorized for the general population.

Overall, immune-compromised persons are 4.8% of persons receiving three doses by year-end 2021, but only 1.26% of persons receiving third doses by year-end 2022; and 1.14% of all vaccinated persons (as of year-end 2022).

We have no reason to expect this exclusion to create bias for the Pfizer-versus-Moderna comparison.

Table S1. Comparison of COVID-19 deaths per text analysis, to ICD-10 codes

Table shows counts, for January 1, 2020, through December 31, 2022, of COVID-19 and other natural-cause deaths for persons aged 15+ determined from our text analysis to be COVID-19 deaths, determined using ICD-10 codes from NCHS (the code for COVID-19 death is U07.1). ICD-10 codes are generated by NCHS, based on the text fields in death certificates. Deaths from natural causes are all deaths except those due to accident, homicide, or suicide.

**Natural cause deaths**	**COVID-19 by Text Analysis**	**non-COVID-19 by Text Analysis**	**Total**
COVID-19 by ICD-10 Code	21,294	676	**21,970**
non-COVID-19 by ICD-10 Code	771	189,929	**190,700**
**Total**	**22,065**	**190,605**	**212,670**

Table S2. Summary Statistics on Vaccination Status

Table provides summary information on vaccine doses for persons aged 15+ vaccinated in Indiana as of December 31, 2021, and December 31, 2022. Estimated Indiana population aged 15+ was 5,459,479 people. Vaccine type for fully vaccinated persons is based on first two doses or, if different, first dose. We report results for 1 or 2 doses based on number of doses, thus treating one J&J dose as equivalent to one mRNA dose; but treat J&J followed by an mRNA dose as 3 doses, thus treating the mRNA dose as a booster dose, and similarly treat J&J followed by two mRNA doses as 4 doses (thus, as receiving two booster doses). More formally, we define persons as receiving a booster dose if they: (1) Received primary 2-dose mRNA vaccination, and then received a third mRNA dose after booster doses for the mRNA vaccines were approved by FDA and CDC (Sept. 24, 2021, for Pfizer; Oct. 21, 2021, for Moderna); or (2) Received an initial J&J dose, and then received an mRNA dose after booster doses for J&J recipients were approved by FDA and CDC (Oct. 21, 2021). **Panel A**. Sample for results in text, excludes immune-compromised persons. **Panel B**. Immune-compromised persons. Percent of population for each row is based on total vaccinees in that row (whether or not immune-compromised).

Panel A. Sample (Excludes Immune-compromised persons)

		Dec. 31, 2021		Dec. 31, 2022	
**No. of Doses**	**Vaccine Type**	**People**	**% of pop.**	**People**	**% of pop.**
1 dose	J&J	224,784	4.12%	174,838	3.20%
	Moderna	98,701	1.81%	119,375	2.19%
	Pfizer	149,768	2.74%	179,625	3.29%
	Novavax	0	0.00%	52	0.00%
	All 1 dose	**473,253**	**8.67%**	**473,890**	**8.68%**
2 doses (excl. J&J plus mRNA booster)	Moderna Only	822,932	15.07%	526,885	9.65%
	Pfizer Only	1,358,450	24.88%	956,275	17.52%
	J&J Only	11,732	0.21%	22,610	0.41%
	Novavax only	0	0.00%	81	0.00%
	Mixed mRNA	4,729	0.09%	8,874	0.16%
	mRNA, then J&J	802	0.01%	850	0.02%
	Other patterns	0	0.00%	14	0.00%
	All primary vax	**2,198,645**	**40.27%**	**1,515,589**	**27.76%**
Booster dose	Moderna Only	302,352	5.54%	385,412	7.06%
	Pfizer Only	474,417	8.69%	654,160	11.98%
	Mixed mRNA	55,347	1.01%	128,123	2.35%
	J&J, then mRNA	28,781	0.53%	79,474	1.46%
	Mixed J&J and mRNA	802	0.01%	2,484	0.05%
	Other patterns	0	0.00%	14	0.00%
	All first booster	**861,699**	**15.78%**	**1,249,667**	**22.89%**
Second booster dose or more	Moderna Only		5.54%	173,993	7.06%
	Pfizer Only		8.69%	253,717	11.98%
	Mixed mRNA		1.01%	108,050	2.35%
	J&J and then 2 mRNA	0	0.00%	14,608	0.27%
	Other patterns	0	0.00%	798	0.01%
	All second booster		**0.00%**	**551,166**	**10.10%**
Other		6,021	0.11%	6,021	0.11%
**All vaccinated**		3,539,618	64.83%	3,796,333	69.54%
**Unvaccinated**		1,919,861	35.17%	1,663,146	30.46%
**Total population**		5,459,479	100%	5,459,479	100%

Panel B. Immune-compromised persons

		Dec. 31, 2021		Dec. 31, 2022	
**No. of Doses**	**Vaccine Type**	**People**	**% of vax pop.**	**People**	**% of vax pop.**
1 dose	J&J	38	0.0169%	32	0.0183%
	Moderna	54	0.0547%	56	0.0469%
	Pfizer	53	0.0354%	51	0.0284%
	Novavax	0		0	0%
	All 1 dose	145	0.0306%	139	0.0293%
2 doses (excl. J&J plus mRNA booster)	Moderna Only	337	0.0409%	295	0.0560%
	Pfizer Only	350	0.0258%	313	0.0327%
	J&J Only	4	0.0341%	5	0.0221%
	Novavax only	0		0	0%
	Mixed mRNA	1	0.0211%	3	0.0338%
	mRNA, then J&J	1	0.1245%	1	0.1175%
	Other patterns	0		0	0%
	All primary vax	693	0.0315%	617	0.0407%
Third dose	Moderna Only	12,019	3.8232%	3,939	1.0117%
	Pfizer Only	29,656	5.8833%	11,218	1.6860%
	Mixed mRNA	1,696	2.9732%	781	0.6059%
	J&J, then mRNA	5	0.0174%	10	0.0126%
	Mixed J&J and mRNA	80	9.0703%	38	1.5067%
	Other patterns	0		0	0%
	All three dose	43,456	4.8009%	15,986	1.2631%
Fourth dose or more	Moderna Only			6,980	3.8569%
	Pfizer Only			16,437	6.0843%
	Mixed mRNA			4,099	3.6550%
	J&J and then 2 mRNA			6	0.0411%
	Other patterns			42	5.0000%
	All 4+ doses			27,564	4.7628%
Other		9	0.1493%	7	0.1161%
**All persons**		44,303	1.2362%	44,313	1.1538%

Table S3. Summary Statistics, Ages 15-59

Table provides selected summary statistics for Indiana vaccinees and unvaccinated persons, age 15-59, as of indicated dates or periods. Total population = 3,914,399 persons. Counts of vaccinated persons are as of the end of the indicated period; deaths and ratios are averages for the period. For 4Q-2021-4Q2022, total population slightly exceeds number unvaccinated or with 1-2 or 3+ doses due to small number of persons with unusual vaccination patterns. CEMP, RMR, and HVBCF are computed for all vaccinated persons together, with any number of doses.

	1Q 2021	2Q 2021	3Q 2021	4Q 2021	1Q 2022	2Q 2022	3Q 2022	4Q 2022	Core Period	Total/Average
**Unvaccinated**	3,643,695	2,309,053	1,924,467	1,695,878	1,542,963	1,520,927	1,505,594	1,495,998	1,542,963	1,495,998
COVID-19 deaths	233	120	438	674	435	19	32	29	1667	1,980
Non-COVID natural deaths	2,049	1,506	1,401	1,314	1,117	1,058	994	1,144	5,338	10,583
COVID-MR	0.006%	0.004%	0.021%	0.037%	0.027%	0.001%	0.002%	0.002%	0.06%	0.07%
Non-COVID NMR	0.054%	0.051%	0.066%	0.073%	0.069%	0.069%	0.066%	0.076%	0.21%	0.39%
CEMP	11.37%	7.97%	31.26%	51.29%	38.94%	1.80%	3.22%	2.53%	31.23%	18.71%
**Vaccinated** (J&J & mixed)	752	148,410	189,837	241,939	313,168	320,487	325,118	331,892	313,168	331,892
**Pfizer vaccinees**										
1 or 2 doses	162,788	931,450	1,190,394	1,161,876	944,322	932,049	926,111	917,779	944,322	917,779
3+ doses	0	0	0	168,784	448,803	471,036	484,229	496,512	448,803	496,512
Total Pfizer vaccinees	162,788	931,450	1,190,394	1,330,660	1,393,125	1,403,085	1,410,340	1,414,291	1,393,125	1,414,291
COVID-19 deaths	1	2	10	31	54	9	5	5	97	117
Non-COVID natural deaths	9	160	303	355	417	360	387	473	1,235	2,464
COVID-MR	0.001%	0.000%	0.001%	0.002%	0.004%	0.001%	0.0004%	0.0004%	0.01%	0.02%
Non-COVID NMR	0.011%	0.029%	0.029%	0.028%	0.031%	0.026%	0.028%	0.033%	0.16%	0.35%
$CEMP_P$	11.11%	1.25%	3.30%	8.73%	12.95%	2.50%	1.29%	1.06%	7.85%	4.75%
$RMR^{CEMP}_{P,unvax}$	0.977	0.157	0.111	0.170	0.333	1.392	0.401	0.417	0.252	0.254
$HVBCF_{P,unvax}$	0.204	0.578	0.432	0.388	0.444	0.373	0.419	0.439	0.771	0.891
**Moderna vaccinees**										
1 or 2 doses	107,164	525,486	609,701	570,228	439,365	434,790	435,797	431,293	439,365	431,293
3+ doses	0	0	0	75,694	225,778	235,110	237,550	240,925	225,778	240,925
Total Moderna vaccinees	107,164	525,486	609,701	645,922	665,143	669,900	673,347	672,218	665,143	672,218
COVID-19 deaths	2	10	21	17	37	7	13	11	85	118
Non-COVID natural deaths	48	239	298	428	398	340	370	397	1,363	2,518
COVID-MR	0.004%	0.003%	0.004%	0.003%	0.006%	0.001%	0.002%	0.002%	0.02%	0.04%
Non-COVID NMR	0.090%	0.076%	0.053%	0.068%	0.061%	0.051%	0.055%	0.059%	0.35%	0.75%
$CEMP_M$	4.17%	4.18%	7.05%	3.97%	9.30%	2.06%	3.51%	2.77%	6.24%	4.69%
$RMR^{CEMP}_{M,unvax}$	0.366	0.525	0.221	0.080	0.239	1.146	1.091	1.093	0.200	0.251
$HVBCF_{M,unvax}$	1.652	1.493	0.793	0.939	0.880	0.738	0.839	0.774	1.715	1.915
$RMR^{1or2}_{P,M}$	2.667	0.299	0.500	2.191	1.305	1.120	0.498	0.263	1.249	1.038
[95% CI]	[0.214, 33.307]	[0.064, 1.384]	[0.210, 1.194]	[1.187, 4.042]	[0.820, 2.078]	[0.371, 3.385]	[0.168, 1.480]	[0.070, 0.985]	[0.909, 1.717]	[0.785, 1.373]
$RMR^{3+}_{P,M}$					1.894	1.817	0	0.933	1.716	93.360
[95% CI]					[0.438, 8.184]	[0.161, 20.460]	---	[1.293, 6.724]	[0.398, 7.395]	[0.367, 2.373]

Table S4. Healthy Vaccinee Bias Correction Factor (HVBCF).

Table shows non-COVID natural deaths, non-COVID Natural Mortality Rate (NCNMR) and healthy vaccinee bias correction factor (NCNMR for vaccinated (with indicated number of doses) divided by NCNMR for unvaccinated), by calendar quarter over 1Q2021-4Q2022, separately for Pfizer and Moderna vaccinees. **Panel A**. Ages 60+. **Panel B**. Ages 15-59. **Panel C**. Age groups; 60-79; 80+. **All panels**. NCNMR is number of Non-COVID-19 natural deaths among persons within indicated age group and vaccination status over indicated period, divided by estimated population in the same age group, vaccination status, and time period. Population with given number of vaccine doses is measured monthly and averaged over each quarter. Counts for 3Q-4Q 2021 are adjusted for IHIE undercount of vaccinated decedents during August-October 2021. For each calendar quarter and age group, we assign the sample population to a vaccine dose category based on the number of people in each category (measured at the beginning of each month) averaged over the three months in the quarter. For each category in the table (by quarter, age group, number of vaccine doses and vaccine manufacturer) we measure the number of non-COVID-19 natural deaths. We then measure HVBCF for each vaccinated group relative to the unvaccinated.

Panel A. Ages 60+

		Pfizer					Moderna				
Period	Vaccine doses	Population at start of period	Population at end of period	Non-COVID nat. deaths	NCNMR	HVBCF vs. unvaxed	Population at start of period	Population at end of period	Non-COVID nat. deaths	NCNMR	HVBCF vs. unvaxed
**1Q 2021**	0	1,545,080	808,818	11,229	0.95%		1,545,080	808,818	11,229	0.95%	
	1	0	144,143	303	0.42%	0.441	0	215,756	1,071	0.99%	1.041
	2	0	201,958	156	0.15%	0.162	0	173,270	403	0.47%	0.488
	1 or 2	0	346,101	459	0.27%	0.278	0	389,026	1,474	0.76%	0.794
**2Q2021**	0	808,818	353,970	6,101	1.05%		808,818	353,970	6,101	1.05%	
	1	144,143	23,573	313	0.37%	0.356	215,756	29,493	590	0.48%	0.459
	2	201,958	566,901	1,847	0.48%	0.458	173,270	523,823	3,552	1.02%	0.971
	1 or 2	346,101	590,474	2,160	0.46%	0.440	389,026	553,316	4,142	0.88%	0.838
**3Q2021**	0	353,970	287,825	5,413	1.69%		353,970	287,825	5,413	1.69%	
	1	23,573	22,487	111	0.48%	0.286	29,493	24,944	248	0.91%	0.540
	2	566,901	604,665	2,559	0.44%	0.259	523,823	548,926	4,282	0.80%	0.473
	1 or 2	590,474	627,152	2,670	0.44%	0.260	553,316	573,870	4,530	0.80%	0.477
**4Q2021**	0	287,825	222,340	5,247	2.06%		287,825	222,340	5,247	2.06%	
	1	22,487	32,278	141	0.51%	0.250	24,944	36,725	244	0.79%	0.385
	2	604,665	314,064	2,667	0.58%	0.282	548,926	314,680	4,354	1.01%	0.490
	1 or 2	627,152	346,342	2,808	0.58%	0.281	573,870	351,405	4,598	0.99%	0.483
	3	0	305,633	470	0.31%	0.150	0	226,658	340	0.30%	0.146
**1Q2022**	0	222,340	194,969	4,762	2.28%		222,340	194,969	4,762	2.28%	
	1	32,278	38,350	176	0.50%	0.218	36,725	43,322	212	0.53%	0.232
	2	314,064	200,276	1,599	0.62%	0.272	314,680	186,724	2,357	0.94%	0.412
	1 or 2	346,342	238,626	1,775	0.61%	0.266	351,405	230,046	2,569	0.88%	0.387
	3 or 4	305,633	416,218	1,631	0.45%	0.198	226,658	345,614	2,296	0.80%	0.352
**2Q2022**	0	194,969	187,943	4,008	2.09%		194,969	187,943	4,008	2.09%	
	1	38,350	36,850	132	0.35%	0.168	43,322	40,905	203	0.48%	0.230
	2	200,276	192,260	1,152	0.59%	0.280	186,724	184,655	1,631	0.88%	0.420
	1 or 2	238,626	229,110	1,284	0.55%	0.262	230,046	225,560	1,834	0.81%	0.385

		Pfizer					Moderna				
Period	Vaccine doses	Population at start of period	Population at end of period	Non-COVID nat. deaths	NCNMR	HVBCF vs. unvaxed	Population at start of period	Population at end of period	Non-COVID nat. deaths	NCNMR	HVBCF vs. unvaxed
	3 or 4	416,218	425,792	1,830	0.43%	0.208	345,614	351,276	2,620	0.75%	0.359
**3Q2022**	0	187,943	183,482	4,205	2.26%		187,943	183,482	4,205	2.26%	
	1	36,850	36,412	112	0.31%	0.135	40,905	40,415	214	0.53%	0.232
	2	192,260	188,078	1,005	0.53%	0.233	184,655	184,293	1,558	0.84%	0.373
	1 or 2	229,110	224,490	1,117	0.49%	0.218	225,560	224,708	1,772	0.79%	0.348
	3 or 4	425,792	430,667	2,030	0.47%	0.209	351,276	353,085	2,774	0.79%	0.348
**4Q2022**	0	183,482	182,314	4,549	2.49%		183,482	182,314	4,549	2.49%	
	1	36,412	35,830	160	0.44%	0.178	40,415	38,838	251	0.63%	0.255
	2	188,078	182,291	1,123	0.61%	0.244	184,293	176,129	1,725	0.96%	0.385
	1 or 2	224,490	218,121	1,283	0.58%	0.233	224,708	214,967	1,976	0.90%	0.361
	3 or 4	430,667	437,840	2,360	0.54%	0.219	353,085	359,697	3,090	0.87%	0.349

Panel B. Ages 15-59

		Pfizer					Moderna				
Period	Vaccine doses	Population at start of period	Population at end of period	Non-COVID nat. deaths	NCNMR	HVBCF vs. unvaxed	Population at start of period	Population at end of period	Non-COVID nat. deaths	NCNMR	HVBCF vs. unvaxed
**1Q 2021**	0	3,914,399	3,643,695	2,049	0.05%		3,914,399	3,643,695	2,049	0.05%	
	1	0	28,556	4	0.03%	0.517	0	38,571	39	0.20%	3.730
	2	0	134,232	5	0.01%	0.137	0	68,593	9	0.03%	0.484
	1 or 2	0	162,788	9	0.01%	0.204	0	107,164	48	0.09%	1.652
**2Q2021**	0	3,643,695	2,309,053	1,506	0.05%		3,643,695	2,309,053	1,506	0.05%	
	1	28,556	145,253	67	0.08%	1.524	38,571	78,557	85	0.15%	2.869
	2	134,232	786,197	93	0.02%	0.399	68,593	446,929	154	0.06%	1.181
	1 or 2	162,788	931,450	160	0.03%	0.578	107,164	525,486	239	0.08%	1.493
**3Q2021**	0	2,309,053	1,924,467	1,401	0.07%		2,309,053	1,924,467	1,401	0.07%	
	1	145,253	130,086	36	0.03%	0.395	78,557	65,722	36	0.05%	0.754
	2	786,197	1,060,308	267	0.03%	0.437	446,929	543,979	262	0.05%	0.799
	1 or 2	931,450	1,190,394	303	0.03%	0.432	525,486	609,701	298	0.05%	0.793
**4Q2021**	0	1,924,467	1,695,878	1,314	0.07%		1,924,467	1,695,878	1,314	0.07%	
	1	130,086	117,490	41	0.03%	0.456	65,722	61,976	50	0.08%	1.079
	2	1,060,308	1,044,386	302	0.03%	0.395	543,979	508,252	374	0.07%	0.979
	1 or 2	1,190,394	1,161,876	342	0.03%	0.401	609,701	570,228	424	0.07%	0.990
	3	0	168,784	13	0.02%	0.212	0	75,694	4	0.01%	0.146
**1Q2022**	0	1,695,878	1,542,963	1,117	0.07%		1,695,878	1,542,963	1,117	0.07%	
	1	117,490	144,532	53	0.04%	0.587	61,976	79,755	35	0.05%	0.716
	2	1,044,386	799,790	276	0.03%	0.434	508,252	359,610	263	0.06%	0.879
	1 or 2	1,161,876	944,322	329	0.03%	0.453	570,228	439,365	298	0.06%	0.856

		Pfizer					Moderna				
Period	Vaccine doses	Population at start of period	Population at end of period	Non-COVID nat. deaths	NCNMR	HVBCF vs. unvaxed	Population at start of period	Population at end of period	Non-COVID nat. deaths	NCNMR	HVBCF vs. unvaxed
	3 or 4	168,784	448,803	88	0.03%	0.413	75,694	225,778	100	0.07%	0.962
**2Q2022**	0	1,542,963	1,520,927	1,058	0.07%		1,542,963	1,520,927	1,058	0.07%	
	1	144,532	142,403	42	0.03%	0.424	79,755	79,275	38	0.05%	0.692
	2	799,790	789,646	209	0.03%	0.381	359,610	355,515	203	0.06%	0.822
	1 or 2	944,322	932,049	251	0.03%	0.387	439,365	434,790	241	0.06%	0.798
	3 or 4	448,803	471,036	109	0.02%	0.343	225,778	235,110	99	0.04%	0.622
**3Q2022**	0	1,520,927	1,505,594	994	0.07%		1,520,927	1,505,594	994	0.07%	
	1	142,403	143,401	40	0.03%	0.426	79,275	80,671	32	0.04%	0.609
	2	789,646	782,710	208	0.03%	0.403	355,515	355,126	215	0.06%	0.921
	1 or 2	932,049	926,111	248	0.03%	0.406	434,790	435,797	247	0.06%	0.864
	3 or 4	471,036	484,229	139	0.03%	0.443	235,110	237,550	123	0.05%	0.792
**4Q2022**	0	1,505,594	1,495,998	1,144	0.08%		1,505,594	1,495,998	1,144	0.08%	
	1	143,401	143,795	54	0.04%	0.493	80,671	80,537	40	0.05%	0.651
	2	782,710	773,984	256	0.03%	0.432	355,126	350,756	205	0.06%	0.762
	1 or 2	926,111	917,779	310	0.03%	0.441	435,797	431,293	245	0.06%	0.741
	3 or 4	484,229	496,512	163	0.03%	0.436	237,550	240,925	152	0.06%	0.834

Table S5. Relative Mortality Risk (Based on CEMP)

Table shows COVID-19 deaths, non-COVID natural deaths, CEMP (COVID Excess Mortality Percentage), and RMR (Relative Mortality Risk) for vaccinated (with indicated number of doses) versus unvaccinated, by calendar quarter over 1Q2021-4Q2022. **Panel A**. Ages 60+. **Panel B**. Ages 15-59. **All panels**. Population with given number of vaccine doses is measured monthly and averaged over each quarter. Counts for 3Q-4Q 2021 are adjusted for IHIE undercount of vaccinated decedents during August-October 2021.

Panel A. Ages 60+

		Pfizer					Moderna				
**Period**	**Vaccine doses**	**COVID deaths**	**Non-COVID nat. deaths**	**CEMP**	**RMR vs. unvaxed**	**95% CI (%)**	**COVID deaths**	**Non-COVID nat. deaths**	**CEMP**	**RMR vs. unvaxed**	**95% CI**
**1Q 2021**	0	2,348	11,229	20.91%			2,348	11,229	20.91%		
	1	11	303	3.63%	0.174	[0.095, 0.317]	54	1,071	5.04%	0.241	[0.183, 0.318]
	2	3	156	1.92%	0.092	[0.029, 0.289]	8	403	1.99%	0.095	[0.047, 0.191]
	1 or 2	14	459	3.05%	0.146	[0.086, 0.249]	62	1,474	4.21%	0.201	[0.155, 0.260]
**2Q2021**	0	380	6,101	6.23%			380	6,101	6.23%		
	1	18	313	5.75%	0.923	[0.568, 1.502]	29	590	4.92%	0.789	[0.536, 1.162]
	2	26	1,847	1.41%	0.226	[0.151, 0.337]	33	3,552	0.93%	0.149	[0.104, 0.213]
	1 or 2	44	2,160	2.04%	0.327	[0.238, 0.449]	62	4,142	1.50%	0.240	[0.183, 0.315]
**3Q2021**	0	1,021	5,413	18.86%			1,021	5,413	18.86%		
	1	12	111	10.98%	0.582	[0.292, 1.161]	9	248	3.83%	0.203	[0.095, 0.433]
	2	147	2,559	5.76%	0.305	[0.250, 0.372]	133	4,282	3.11%	0.165	[0.134, 0.203]
	1 or 2	159	2,670	5.98%	0.317	[0.261, 0.384]	142	4,530	3.15%	0.167	[0.137, 0.204.]
**4Q2021**	0	1,819	5,247	34.66%			1,819	5,247	34.66%		
	1	17	141	12.32%	0.355	[0.214, 0.590]	34	244	13.81%	0.398	[0.276, 0.575]
	2	296	2,667	11.08%	0.320	[0.280, 0.366]	356	4,354	8.17%	0.236	[0.209, 0.267]
	1 or 2	313	2,808	11.15%	0.322	[0.282, 0.367[	390	4,598	8.47%	0.244	[0.217, 0.275]
	3	21	470	4.47%	0.129	[0.083, 0.200]	8	340	2.35%	0.068	[0.034, 0.137]
**1Q2022**	0	1,605	4,762	33.70%			1,605	4,762	33.70%		
	1	35	176	19.89%	0.590	[0.409, 0.852]	42	212	19.81%	0.588	[0.420, 0.822]
	2	295	1,599	18.45%	0.547	[0.478, 0.627]	317	2,357	13.45%	0.399	[0.350, 0.455]
	1 or 2	330	1,775	18.59%	0.552	[0.484, 0.628]	359	2,569	13.97%	0.415	[0.366, 0.469]
	3 or 4	100	1,631	6.13%	0.182	[0.147, 0.224]	105	2,296	4.57%	0.136	[0.111, 0.166]
**2Q2022**	0	85	4,008	2.12%			85	4,008	2.12%		
	1	2	132	1.52%	0.714	[0.174, 2.935]	9	203	4.43%	2.091	[1.037, 4.216]
	2	21	1,152	1.82%	0.860	[0.531, 1.392]	50	1,631	3.07%	1.446	[1.015, 2.060]
	1 or 2	23	1,284	1.79%	0.845	[0.531,1.345]	59	1,834	3.22%	1.517	[1.083, 2.124]
	3 or 4	25	1,830	1.37%	0.644	[0.411, 1.010]	53	2,620	2.02%	0.954	[0.674, 1.349]
**3Q2022**	0	169	4,205	4.02%			169	4,205	4.02%		
	1	7	112	6.25%	1.555	[0.714, 3.389]	5	214	2.34%	0.581	[0.236, 1.430]
	2	38	1,005	3.78%	0.941	[0.657, 1.347]	69	1,558	4.43%	1.102	[0.828, 1.467]
	1 or 2	45	1,117	4.03%	1.002	[0.717, 1.402]	74	1,772	4.18%	1.039	[0.786, 1.373]
	3 or 4	76	2,030	3.74%	0.932	[0.707, 1.227]	92	2,774	3.32%	0.825	[0.637, 1.069]
**4Q2022**	0	184	4,549	4.04%			184	4,549	4.04%		
	1	5	160	3.13%	0.773	[0.313, 1.905]	7	251	2.79%	0.690	[0.321, 1.482]
	2	48	1,123	4.27%	1.057	[0.764, 1.462]	61	1,725	3.54%	0.874	[0.651, 1.174]
	1 or 2	53	1,283	4.13%	1.021	[0.748, 1.395]	68	1,976	3.44%	0.851	[0.641, 1.129]
	3 or 4	75	2,360	3.18%	0.786	[0.598, 1.032]	122	3,090	3.95%	0.976	[0.773, 1.233]

Panel B. Ages 15-59

		Pfizer					Moderna				
**Period**	**Vaccine doses**	**COVID deaths**	**Non-COVID nat. deaths**	**CEMP**	**RMR vs. unvaxed**	**95% CI (%)**	**COVID deaths**	**Non-COVID nat. deaths**	**CEMP**	**RMR vs. unvaxed**	**95% CI**
**1Q 2021**	0	233	2,049	11.37%			233	2,049	11.37%		
	1	1	4	25.00%	2.199	[0.245, 19.761]	2	39	5.13%	0.451	[0.108, 1.880]
	2	0	5	0.00%	0.000	----	0	9	0.00%	0.000	----
	1 or 2	1	9	11.11%	0.977	[0.123, 7.750]	2	48	4.17%	0.366	[0.088, 1.518]
**2Q2021**	0	120	1,506	7.97%			120	1,506	7.97%		
	1	1	67	1.49%	0.187	[0.026, 1.362]	4	85	4.71%	0.591	[0.213, 1.638]
	2	1	93	1.08%	0.135	[0.019, 0.977]	6	154	3.90%	0.489	[0.212, 1.129]
	1 or 2	2	160	1.25%	0.157	[0.038, 0.641]	10	239	4.18%	0.525	[0.272, 1.016]
**3Q2021**	0	438	1,401	31.24%			438	1,401	31.24%		
	1	4	36	11.54%	0.369	[0.111, 1.226]	4	36	11.54%	0.369	[0.111, 1.226]
	2	6	267	2.37%	0.076	[0.031, 0.185]	16	262	6.28%	0.201	[0.114, 0.356]
	1 or 2	10	303	3.46%	0.111	[0.054, 0.226]	21	298	6.92%	0.221	[0.132, 0.372]
**4Q2021**	0	674	1,314	51.29%			674	1,314	51.29%		
	1	8	41	20.51%	0.400	[0.186, 0.861]	1	50	2.08%	0.041	[0.006, 0.295]
	2	23	302	7.48%	0.146	[0.094, 0.227]	16	374	4.40%	0.086	[0.052, 0.143]
	1 or 2	31	342	9.03%	0.176	[0.120, 0.259]	17	424	4.12%	0.080	[0.049, 0.132]
	3	0	13	0.00%	0.000	----	0	4	0.00%	0.000	
**1Q2022**	0	435	1,117	38.94%			435	1,117	38.94%		
	1	10	53	18.87%	0.485	[0.244, 0.961]	5	35	14.29%	0.367	[0.143, 0.943]
	2	39	276	14.13%	0.363	[0.255, 0.517]	29	263	11.03%	0.283	[0.190, 0.422]
	1 or 2	49	329	14.89%	0.382	[0.278, 0.527]	34	298	11.41%	0.293	[0.202, 0.425]
	3	5	88	5.68%	0.146	[0.059, 0.362]	3	100	3.00%	0.077	[0.024, 0.244]
**2Q2022**	0	19	1,058	1.80%			19	1,058	1.80%		
	1	0	42	0%	0	---	0	38	0%	0	----
	2	7	209	3.35%	1.865	[0.774, 4.494]	6	203	2.96%	1.646	[0.649, 4.173]
	1 or 2	7	251	2.79%	1.553	[0.646, 3.736]	6	241	2.49%	1.386	[0.548, 3.509]
	3 or 4	2	109	1.83%	1.022	[0.235, 4.448]	1	99	1.01%	0.563	[0.074, 4.250]
**3Q2022**	0	32	994	3.22%			32	994	3.22%		
	1	1	40	2.50%	0.777	[0.103, 5.832]	3	32	9.38%	2.912	[0.847, 10.016]
	2	4	208	1.92%	0.597	[0.209, 1.708]	7	215	3.26%	1.011	[0.440, 2.322]
	1 or 2	5	248	2.02%	0.626	[0.241, 1.624]	10	247	4.05%	1.258	[0.610, 2.594]
	3 or 4	0	139	0%	0		3	123	2.44%	0.758	[0.228, 2.512]
**4Q2022**	0	29	1,144	2.53%			29	1,144	2.53%		
	1	0	54	0%	0	----	0	40	0%	0	-----
	2	3	256	1.17%	0.462	[0.140, 1.530]	9	205	4.39%	1.732	[0.808, 3.713]
	1 or 2	3	310	0.97%	0.382	[0.115, 1.262]	9	245	3.67%	1.449	[0.677, 3.101]
	3 or 4	2	163	1.23%	0.484	[0.114, 2.049]	2	152	1.32%	0.519	[0.123, 2.198]

Table S6. Summary Statistics, Separately for Ages 60-79 and 80+

Table provides selected summary statistics for Indiana vaccinees and unvaccinated persons, age 60-79 and 80+, as of indicated dates or periods. Total population: 1,285,407 persons, aged 60-79, and 259,673 persons, aged 80+. Vaccinee counts are at end of indicated period; deaths and ratios are averages for the period. Total population slightly exceeds number unvaccinated or with 1-2 or 3+ doses due to small number of persons with unusual vaccination patterns. CEMP, RMR, and HVBCF are for all vaccinated persons, with any number of doses.

Panel A. ages 60-79

	1Q 2021	2Q 2021	3Q 2021	4Q 2021	1Q 2022	2Q 2022	3Q 2022	4Q 2022	Core Period	Total/Average
**Unvaccinated**	734,684	314,318	255,005	200,431	175,510	171,076	167,722	164,414	175,510	164,414
COVID-19 deaths	1,190	268	722	1,262	1,053	44	72	91	3305	4,702
Non-COVID natural deaths	6,066	3,592	3,149	3,028	2,726	2,351	2,458	2,646	12,495	26,016
COVID-MR	0.118%	0.051%	0.254%	0.554%	0.560%	0.025%	0.043%	0.055%	0.73%	0.65%
Non-COVID NMR	0.601%	0.685%	1.106%	1.330%	1.450%	1.357%	1.451%	1.593%	2.75%	3.59%
CEMP	19.62%	7.46%	22.94%	41.66%	38.63%	1.87%	2.93%	3.44%	26.45%	18.07%
**Vaccines** (J&J and mixed)	749	42,469	50,411	79,813	103,683	107,204	109,657	114,834	103,683	114,834
**Pfizer vaccinees**										
1 or 2 doses	265,326	492,907	525,946	298,099	203,570	195,546	191,732	186,331	203,570	186,331
3+ doses				249,489	346,896	354,970	359,007	364,943	346,896	364,943
Total Pfizer vaccinees	265,326	492,907	525,946	547,588	550,466	550,516	550,739	551,274	550,466	551,274
COVID-19 deaths	2	18	80	185	231	19	59	48	514	642
Non-COVID natural deaths	161	1,105	1,334	1,670	1,790	1,664	1,638	1,878	5,899	11,240
COVID-MR	0.002%	0.005%	0.016%	0.034%	0.042%	0.003%	0.011%	0.009%	0.13%	0.23%
Non-COVID NMR	0.121%	0.291%	0.262%	0.311%	0.326%	0.302%	0.297%	0.341%	1.45%	4.08%
$CEMP_{Pfizer}$	1.24%	1.63%	6.00%	11.08%	12.91%	1.14%	3.60%	2.56%	8.71%	5.71%
$RMR^{CEMP}_{P,unvax}$	0.063	0.218	0.261	0.266	0.334	0.610	1.230	0.743	0.329	0.316
$HVBCF_{P,unvax}$	0.202	0.426	0.237	0.234	0.225	0.223	0.205	0.214	0.527	1.136
**Moderna vaccinees**										
1 or 2 doses	284,648	435,713	454,045	283,158	181,429	177,513	176,786	169,356	181,429	169,356
3+ doses	0	0	0	174,417	274,319	279,098	280,503	285,529	274,319	285,529
Total Moderna vaccinees	284,648	435,713	454,045	457,575	455,748	456,611	457,289	454,885	455,748	454,885
COVID-19 deaths	22	39	54	145	215	47	56	66	453	644
Non-COVID natural deaths	505	1,787	1,951	2,107	2,103	1,976	1,978	2,212	7,948	14,619
COVID-MR	0.015%	0.011%	0.012%	0.032%	0.047%	0.010%	0.012%	0.014%	0.12%	0.28%
Non-COVID NMR	0.355%	0.496%	0.439%	0.462%	0.461%	0.433%	0.433%	0.485%	2.15%	6.43%
$CEMP_{Moderna}$	4.36%	2.18%	2.77%	6.88%	10.22%	2.38%	2.83%	2.98%	5.70%	4.41%
$RMR^{CEMP}_{M,unvax}$	0.222	0.293	0.121	0.165	0.265	1.272	0.966	0.867	0.216	0.244
$HVBCF_{M,unvax}$	0.591	0.725	0.396	0.348	0.318	0.319	0.298	0.304	0.782	1.791
$RMR^{1or2}_{P,M}$	0.285	0.746	2.163	1.695	1.272	0.385	1.449	0.999	1.559	1.409
[95% CI]	[0.066, 1.227]	[0.425, 1.311]	[1.464, 3.196]	[1.338, 2.147]	[1.017, 1.590]	[0.181, 0.818]	[0.779, 2.696]	[0.563, 1.774]	[1.352,1.796]	[1.238, 1.603]
$RMR^{3+}_{P,M}$				1.447	1.592	0.638	1.158	0.764	1.547	1.134
[95% CI]				[0.450, 4.660]	[1.029, 2.463]	[0.293, 1.389]	[0.728, 1.842]	[0.463, 1.259]	[1.030, 2.324]	[0.900, 1.444]

Panel B. ages 80+

	1Q 2021	2Q 2021	3Q 2021	4Q 2021	1Q 2022	2Q 2022	3Q 2022	4Q 2022	Core Period	Total/Average
**Unvaccinated**	74,134	39,652	32,820	21,909	19,459	16,867	15,760	17,900	19,459	17,900
COVID-19 deaths	1,158	112	299	557	552	41	97	93	1520	2,909
Non-COVID natural deaths	5,163	2,509	2,263	2,219	2,036	1,657	1,747	1,903	9,027	19,497
COVID-MR	0.694%	0.197%	0.825%	2.035%	2.669%	0.226%	0.595%	0.553%	3.25%	2.10%
Non-COVID NMR	3.093%	4.410%	6.245%	8.109%	9.843%	9.123%	10.709%	11.307%	19.29%	14.05%
CEMP	22.43%	4.46%	13.21%	25.10%	27.11%	2.47%	5.55%	4.89%	16.84%	14.92%
**Vaccines** (J&J and mixed)	386	4,851	5,822	12,889	15,924	18,195	18,991	17,307	15,924	17,307
**Pfizer vaccinees**										
1 or 2 doses	80,775	97,567	101,206	48,243	35,056	33,564	32,758	31,790	35,056	31,790
3+ doses	0	0	0	56,144	69,322	70,822	71,660	72,897	69,322	72,897
Total Pfizer vaccinees	80,775	97,567	101,206	104,387	104,378	104,386	104,418	104,687	104,378	104,687
COVID-19 deaths	12	26	79	148	199	29	62	80	452	635
Non-COVID natural deaths	298	1,055	1,324	1,603	1,616	1,450	1,509	1,765	5,598	10,620
COVID-MR	0.030%	0.029%	0.079%	0.144%	0.191%	0.028%	0.059%	0.077%	0.49%	1.21%
Non-COVID NMR	0.738%	1.183%	1.332%	1.559%	1.548%	1.389%	1.445%	1.688%	6.05%	20.29%
$CEMP_{Pfizer}$	4.03%	2.46%	4.49%	8.71%	12.31%	2.00%	4.11%	4.53%	8.07%	5.98%
$RMR_{P,unvax}^{CEMP}$	0.180	0.552	0.449	0.367	0.454	0.808	0.740	0.928	0.480	0.401
$HVBCF_{P,unvax}$	0.239	0.268	0.213	0.192	0.157	0.152	0.135	0.149	0.314	1.444
**Moderna vaccinees**										
1 or 2 doses	104,378	117,603	119,825	68,247	48,617	48,047	47,922	45,611	48,617	45,611
3+ doses	0	0	0	52,241	71,295	72,178	72,582	74,168	71,295	74,168
Total Moderna vaccinees	104,378	117,603	119,825	120,488	119,912	120,225	120,504	119,779	119,912	119,779
COVID-19 deaths	40	23	90	254	249	65	110	124	616	955
Non-COVID natural deaths	969	2,355	2,590	2,836	2,762	2,478	2,568	2,854	10,543	19,412
COVID-MR	0.077%	0.021%	0.076%	0.211%	0.207%	0.054%	0.091%	0.103%	0.55%	1.59%
Non-COVID NMR	1.857%	2.122%	2.182%	2.360%	2.298%	2.064%	2.134%	2.376%	9.40%	32.41%
$CEMP_{Moderna}$	4.13%	0.98%	3.47%	8.96%	9.02%	2.62%	4.28%	4.34%	5.84%	4.92%
$RMR_{M,unvax}^{CEMP}$	0.184	0.219	0.262	0.357	0.333	1.060	0.772	0.889	0.347	0.330
$HVBCF_{M,unvax}$	0.600	0.481	0.349	0.291	0.233	0.226	0.199	0.210	0.487	2.307
**Pfizer vs. Moderna**										
$RMR_{P,M}^{1or2}$	0.976	2.523	1.713	1.069	1.399	0.779	0.828	1.467	1.430	1.514
[95% CI]	[0.505, 1.884]	[1.433, 4.443]	[1.197, 2.452]	[0.853, 1.339]	[1.108, 1.767]	[0.410, 1.483]	[0.506, 1.357]	[0.911, 2.364]	[1.242, 1.645]	[1.181, 1.514]
$RMR_{P,M}^{3+}$				2.203	1.169	0.743	1.104	0.871	1.182	1.011
[95% CI]				[0.681, 7.128]	[0.798, 1.713]	[0.403, 1.368]	[0.726, 1.679]	[0.606, 1.250]	[0.829, 1.687]	[0.825, 1.238]

Table S7. Multivariate Logit Results for *RMR*

Table compares *RMR* (based on *CEMP*) to *RMR* estimates from multivariate regression, for vaccinated (with indicated number of doses) versus unvaccinated persons, by calendar quarter over 1Q2021-4Q2022, separately for ages 15-59 and 60+. 95% CI is for multivariate estimate. The multivariate regression includes the following covariates: quadratic in age, categorical indicators for sex, race, education, marital status, and quintiles of the Graham Social Deprivation Index.

				Ages 15-59				Ages 60+	
**Period**	**Vaccine doses**	***CEMP***	***RMR* vs. unvaxed**	***RMR* (multivariate)**	**95% CI (%)**	***CEMP***	***RMR* vs. unvaxed**	***RMR* (multivariate)**	**95% CI**
**1Q 2021**	0	0.1137				0.2091			
	1	0.0698	0.6135	0.7600	[0.2308, 2.5027]	0.0473	0.2262***	0.2195***	[0.1702, 0.2831]
	2	0.0000				0.0197	0.0941***	0.0919***	[0.0504, 0.1675]
	1 or 2	0.0526	0.4628	0.5488	[0.1675, 1.7982]	0.0393	0.1880***	0.1828***	[0.1445, 0.2314]
**2Q2021**	0	0.0797				0.0623			
	1	0.0330	0.4137**	0.3900*	[0.1691, 0.8991]	0.0467	0.7492*	0.7413	[0.5454, 1.0074]
	2	0.0282	0.3542***	0.3168**	[0.1414, 0.7096]	0.0109	0.1751***	0.1955***	[0.1479, 0.2586]
	1 or 2	0.0302	0.3794***	0.3471***	[0.1901, 0.6338]	0.0167	0.2685***	0.2969***	[0.2382, 0.3702]
**3Q2021**	0	0.2861				0.1523			
	1	0.0877	0.2808***	0.2544***	[0.1300, 0.4976]	0.0726	0.3848***	0.3842***	[0.2725, 0.5418]
	2	0.0429	0.1372***	0.1272***	[0.0775, 0.2088]	0.0409	0.2170***	0.2239***	[0.1927, 0.2601]
	1 or 2	0.0524	0.1678***	0.1547***	[0.1030, 0.2323]	0.0436	0.2312***	0.2382***	[0.2068, 0.2744]
	3								
**4Q2021**	0	0.5034				0.3241			
	1	0.1343	0.2619***	0.2524***	[0.1653, 0.3854]	0.1412	0.4072***	0.4036***	[0.3269, 0.4982]
	2	0.0574	0.1119***	0.1091***	[0.0772, 0.1541]	0.0930	0.2682***	0.2822***	[0.2559, 0.3112]
	1 or 2	0.0753	0.1469***	0.1427***	[0.1084, 0.1877]	0.0979	0.2823***	0.2958***	[0.2696, 0.3245]
	3	0.0000				0.0350	0.1125***	0.1085***	[0.0750, 0.1571]
**1Q2022**	0	0.3894				0.3370			
	1	0.1563	0.4012***	0.3994***	[0.2567, 0.6216]	0.1931	0.5730***	0.5758***	[0.4723, 0.7020]
	2	0.1255	0.3221***	0.3027***	[0.2275, 0.4028]	0.1552	0.4605***	0.4658***	[0.4205, 0.5161]
	1 or 2	0.1324	0.3400***	0.3256***	[0.2531, 0.4188]	0.1606	0.4765***	0.4827***	[0.4383, 0.5315]
	3	0.0485	0.1244***	0.1119***	[0.0603, 0.2077]	0.0513	0.1522***	0.1557***	[0.1343, 0.1804]
**2Q2022**	0	0.0180				0.0212			
	1	0.0000		1.0000		0.0286	1.3496	1.3572	[0.7834, 2.3515]
	2	0.0309	1.7195	2.0699	[0.9461, 4.5287]	0.0250	1.1772	1.1310	[0.8133, 1.5726]
	1 or 2	0.0236	1.3138	1.6062	[0.7237, 3.5647]	0.0256	1.2055	1.1401	[0.8314, 1.5635]
	3	0.0122	0.6791	0.6528	[0.1821, 2.3403]	0.0185	0.8701	0.7974	[0.5800, 1.0963]
	4	0.0000				0.0102	0.4787	0.4144	[0.1003, 1.7119]
**3Q2022**	0	0.0322				0.0402			
	1	0.0420	1.3051	1.4007	[0.5215, 3.7624]	0.0335	0.8327	0.7844	[0.4702, 1.3084]
	2	0.0249	0.7748	0.8066	[0.4011, 1.6223]	0.0407	1.0119	0.9509	[0.7394, 1.2229]
	1 or 2	0.0286	0.8875	0.9041	[0.4885, 1.6734]	0.0395	0.9829	0.9139	[0.7165, 1.1656]
	3	0.0128	0.3970*	0.4109	[0.1328, 1.2710]	0.0350	0.8717	0.7860*	[0.6235, 0.9909]
	4	0.0357	1.1094	1.2246	[0.1369, 10.9526]	0.0282	0.7029*	0.5964**	[0.4098, 0.8679]
**4Q2022**	0	0.0253				0.0404			
	1	0.0124	0.4900	0.5096	[0.1172, 2.2150]	0.0266	0.6582	0.6537	[0.3899, 1.0958]
	2	0.0251	0.9883	0.9223	[0.4598, 1.8503]	0.0376	0.9301	0.9135	[0.7173, 1.1633]
	1 or 2	0.0219	0.8629	0.8185	[0.4183, 1.6017]	0.0357	0.8837	0.8660	[0.6865, 1.0924]
	3	0.0096	0.3769	0.4109	[0.1183, 1.4275]	0.0353	0.8719	0.8166	[0.6519, 1.0228]
	4	0.0149	0.5888	0.5985	[0.0756, 4.7350]	0.0372	0.9200	0.8074	[0.6164, 1.0577]

Table S8. Multivariate Logit Results for *RMR*, Pfizer vs. Moderna

Table compares *RMR* (based on *CEMP*) to *RMR* estimates from multivariate regression, for vaccinated (with indicated number of doses) versus unvaccinated persons, by calendar quarter over 1Q2021-4Q2022, separately for ages 15-59 and 60+. 95% CI is for multivariate estimate. The multivariate regression includes the following covariates: quadratic in age, categorical indicators for sex, race, education, marital status, and quintiles of the Graham Social Deprivation Index. n.a. indicates that regression did not converge due to small number of vaccinated decedents.

Panel A. Ages 60+

		Pfizer, Ages 60+				Moderna, Ages 60+				Pfizer/Moderna ratio		
**Period**	**Vaccine doses**	***CEMP***	***RMR* vs. unvaxed**	***RMR* (multivariate)**	**95% CI (%)**	***CEMP***	***RMR* vs. unvaxed**	***RMR* (multivariate)**	**95% CI**	**Univariate ratio**	**Multivariate ratio**	**95% CI (multivariate)**
**1Q 2021**	0	20.91%				20.91%						
	1	3.63%	0.174	0.161	[0.088, 0.294]	5.04%	0.241	0.237	[0.179, 0.313]	0.720	0.749	[0.382, 1.467]
	2	1.92%	0.092	0.087	[0.028, 0.271]	1.99%	0.095	0.094	[0.046, 0.190]	0.969	1.016	[0.159, 6.468]
	1 or 2	3.05%	0.146	0.136	[0.080, 0.232]	4.21%	0.201	0.198	[0.153, 0.257]	0.725	0.720	[0.391, 1.328]
**2Q2021**	0	6.23%				6.23%						
	1	5.75%	0.923	0.859	[0.530, 1.394]	4.92%	0.789	0.812	[0.549, 1.203]	1.170	0.833	[0.448, 1.549]
	2	1.41%	0.226	0.236	[0.157, 0.354]	0.93%	0.149	0.176	[0.123, 0.253]	1.515	1.589	[0.939, 2.687]
	1 or 2	2.04%	0.327	0.333	[0.242, 0.459]	1.50%	0.240	0.280	[0.212, 0.369]	1.361	1.308	[0.879, 1.945]
**3Q2021**	0	18.86%				18.86%						
	1	10.98%	0.582	0.549	[0.276, 1.091]	3.83%	0.203	0.211	[0.098, 0.455]	2.869	2.744	[0.794, 9.479]
	2	5.76%	0.305	0.300	[0.245, 0.368]	3.11%	0.165	0.176	[0.144, 0.217]	1.851	1.884	[1.429, 2.483]
	1 or 2	5.98%	0.317	0.312	[0.256, 0.379]	3.15%	0.167	0.178	[0.146, 0.217]	1.897	1.941	[1.488, 2.533]
**4Q2021**	0	34.66%				34.66%						
	1	12.32%	0.355	0.326	[0.196, 0.541]	13.81%	0.398	0.420	[0.288, 0.613]	0.892	0.898	[0.453, 1.780]
	2	11.08%	0.320	0.326	[0.284, 0.374]	8.17%	0.236	0.257	[0.227, 0.290]	1.356	1.394	[1.176, 1.651]
	1 or 2	11.15%	0.322	0.326	[0.285, 0.373]	8.47%	0.244	0.265	[0.235, 0.298]	1.316	1.354	[1.151, 1.594]
	3	4.47%	0.129	0.141	[0.090, 0.220]	2.35%	0.068	0.081	[0.040, 0.163]	1.899	1.699	[0.706, 4.084]
**1Q2022**	0	33.70%				33.70%						
	1	19.89%	0.590	0.591	[0.405, 0.864]	19.81%	0.588	0.611	[0.434, 0.860]	1.004	0.992	[0.595, 1.655]
	2	18.45%	0.547	0.542	[0.472, 0.623]	13.45%	0.399	0.412	[0.361, 0.470]	1.372	1.368	[1.150, 1.628]
	1 or 2	18.59%	0.552	0.547	[0.479, 0.625]	13.97%	0.415	0.429	[0.378, 0.486]	1.330	1.328	[1.127, 1.564]
	3 or 4	6.13%	0.182	0.180	[0.145, 0.222]	4.57%	0.136	0.146	[0.119, 0.180]	1.341	1.310	[0.988, 1.736]
**2Q2022**	0	2.12%				2.12%						
	1	1.52%	0.714	0.699	[0.166, 2.948]	4.43%	2.091	1.767	[0.875, 3.567]	0.342	0.166	[0.037, 0.752]
	2	1.82%	0.860	0.797	[0.486, 1.307]	3.07%	1.446	1.303	[0.903, 1.882]	0.595	0.630	[0.375, 1.059]
	1 or 2	1.79%	0.845	0.795	[0.492, 1.287]	3.22%	1.517	1.357	[0.958, 1.922]	0.557	0.585	[0.361, 0.948]
	3 or 4	1.37%	0.644	0.537	[0.334, 0.863]	2.02%	0.954	0.838	[0.578, 1.215]	0.675	0.680	[0.410, 1.127]
**3Q2022**	0	4.02%				4.02%						
	1	6.25%	1.555	1.453	[0.650, 3.250]	2.34%	0.581	0.507	[0.201, 1.281]	2.675	3.540	[0.856, 14.641]
	2	3.78%	0.941	0.890	[0.616, 1.285]	4.43%	1.102	0.971	[0.727, 1.296]	0.854	0.908	[0.608, 1.355]
	1 or 2	4.03%	1.002	0.954	[0.675, 1.348]	4.18%	1.039	0.916	[0.690, 1.216]	0.965	1.038	[0.709, 1.517]
	3 or 4	3.74%	0.932	0.832	[0.627, 1.105]	3.32%	0.825	0.705	[0.537, 0.925]	1.129	1.205	[0.881, 1.648]
**4Q2022**	0	4.04%				4.04%						
	1	3.13%	0.773	0.773	[0.316, 1.892]	2.79%	0.690	0.671	[0.314, 1.438]	1.121	1.013	[0.214, 4.787]
	2	4.27%	1.057	1.039	[0.752, 1.437]	3.54%	0.874	0.836	[0.622, 1.123]	1.209	1.282	[0.869, 1.891]

	Pfizer, Ages 60+					Moderna, Ages 60+				Pfizer/Moderna ratio		
**Period**	**Vaccine doses**	***CEMP***	***RMR* vs. unvaxed**	***RMR* (multivariate)**	**95% CI (%)**	***CEMP***	***RMR* vs. unvaxed**	***RMR* (multivariate)**	**95% CI**	**Univariate ratio**	**Multivariate ratio**	**95% CI (multivariate)**
	1 or 2	4.13%	1.021	1.015	[0.742, 1.390]	3.44%	0.851	0.816	[0.614, 1.083]	1.200	1.302	[0.896, 1.892]
	3 or 4	3.18%	0.786	0.724	[0.548, 0.956]	3.95%	0.976	0.895	[0.706, 1.136]	0.805	0.825	[0.614, 1.108]

Panel B. Ages 15-59

	Pfizer, Ages 15-59					Moderna, Ages 15-59				Pfizer/Moderna ratio		
**Period**	**Vaccine doses**	***CEMP***	**RMR (univariate)**	**RMR (univariate)**	**95% CI**	***CEMP***	***RMR* vs. unvaxed**	***RMR* (multivariate)**	**95% CI**	**Univariate ratio**	**Multivariate ratio**	**95% CI (multivariate)**
**1Q 2021**	0	11.37%				11.37%						
	1 or 2	11.11%	0.977	0.748	[0.088, 6.323]	4.17%	0.366	0.489	[0.116, 2.065]	2.667		
**2Q2021**	0	7.97%				7.97%						
	1 or 2	1.25%	0.157	0.141	[0.033, 0.603]	4.18%	0.525	0.489	[0.249, 0.960]	0.299	0.198	[0.034, 1.156]
**3Q2021**	0	31.24%				31.24%						
	1 or 2	3.46%	0.111	0.096	[0.046, 0.203]	6.92%	0.221	0.223	[0.132, 0.377]	0.500	0.527	[0.212, 1.309]
**4Q2021**	0	51.29%				51.29%						
	1 or 2	9.03%	0.176	0.164	[0.110, 0.244]	4.12%	0.080	0.082	[0.050, 0.135]	2.191	2.181	[1.138, 4.181]
	3 or 4	0%	0	0	----	0%	0	0	----	n.a.	n.a.	
**1Q2022**	0	38.94%				38.94%						
	1 or 2	14.89%	0.382	0.358	[0.255, 0.503]	11.41%	0.293	0.280	[0.190, 0.412]	1.305	1.353	[0.825, 2.218]
	3 or 4	5.68%	0.146	0.114	[0.043, 0.303]	3.00%	0.077	0.059	[0.015, 0.230]	1.894	5.932	[0.859, 40.949]
**2Q2022**	0	1.80%				1.80%						
	1 or 2	2.79%	1.553	1.811	[0.669, 4.908]	2.49%	1.386	1.797	[0.651, 4.961]	1.120	1.511	[0.432, 5.287]
	3 or 4	1.83%	1.022	0.943	[0.173, 5.133]	1.01%	0.563	0.591	[0.075, 4.645]	1.817	n.a.	
**3Q2022**	0	3.22%				3.22%						
	1 or 2	2.02%	0.626	0.680	[0.267, 1.728]	4.05%	1.258	1.286	[0.623, 2.657]	0.498	0.433	[0.125, 1.496]
	3 or 4	0%	0	0	----	2.44%	0.758	0.833	[0.232, 2.988]		n.a.	
**4Q2022**	0	2.53%				2.53%						
	1 or 2	0.97%	0.382	0.311	[0.091, 1.066]	3.67%	1.449	1.401	[0.643, 3.055]	0.263	0.323	[0.085, 1.236]
	3 or 4	1.23%	0.484	0.524	[0.105, 2.628]	1.32%	0.519	0.567	[0.126, 2.545]	0.933	n.a.	

Table S9. Vaccine Approval Dates

Table shows CDC approval dates for indicated vaccine types and number of doses. CDC approval is soon after FDA approval. Some approvals were for limited age groups or populations (e.g., high-risk workers).

Vaccine	Primary vaccination	1st booster dose	2nd booster dose
Pfizer	2020.12.14	2021.09.24	2022.04.30
Moderna	2020.12.18	2021.10.21	2022.04.30
J&J	2021.02.26		
Novavax	2022.07.13		

Figure S1. Correlation between Natural Mortality in 2019 and COVID-19 Mortality in 2020

Figure shows scatterplot of natural mortality in Indiana over April–December 2019 against COVID-19 mortality over April–December 2020, for groups defined by age (15–39, 40–49, 50–59, 60–69, 70–79, 80–89, 90+), gender, race/ethnicity, and zip-SES quintile. Figure 1 also shows a best-fit regression line and a Pearson correlation coefficient.

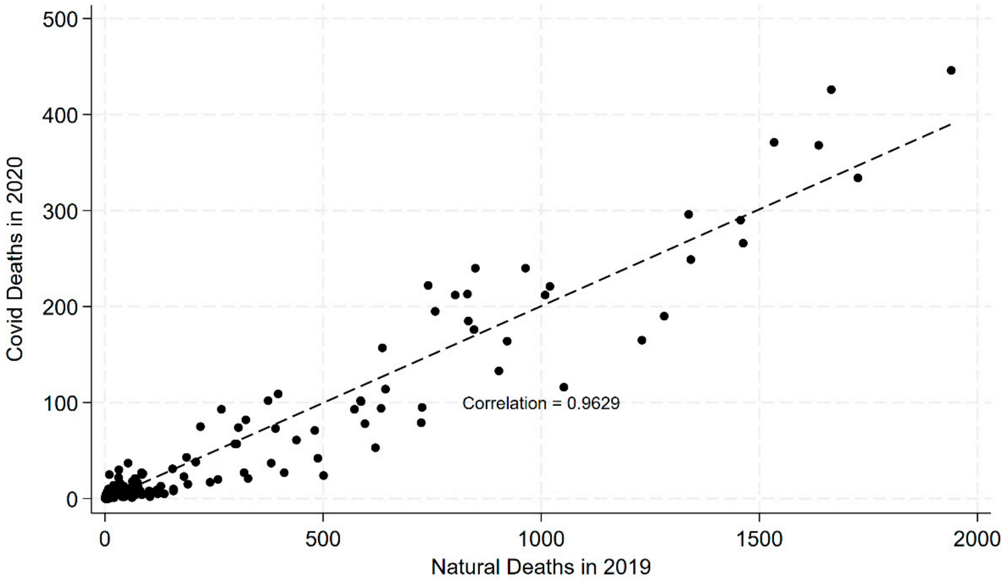

Figure S2. Correlation between Natural Mortality Rates in 2019 and COVID-19 Mortality Rates in 2020.

Figure is similar to Figure S1 but shows scatterplot of natural mortality *rates* in Indiana over April-December 2019 against COVID-19 mortality over April-December 2020, for groups defined by age (15-39, 40-49, 50-59, 60-69, 70-79, 80-89, 90+)*gender*race/ethnicity, best-fit regression line, and Pearson correlation coefficient. In contrast to Figure S1, which provides a scatterplot for mortality *counts*, the population groups are not also divided into SES quintiles due to limited Indiana population size.

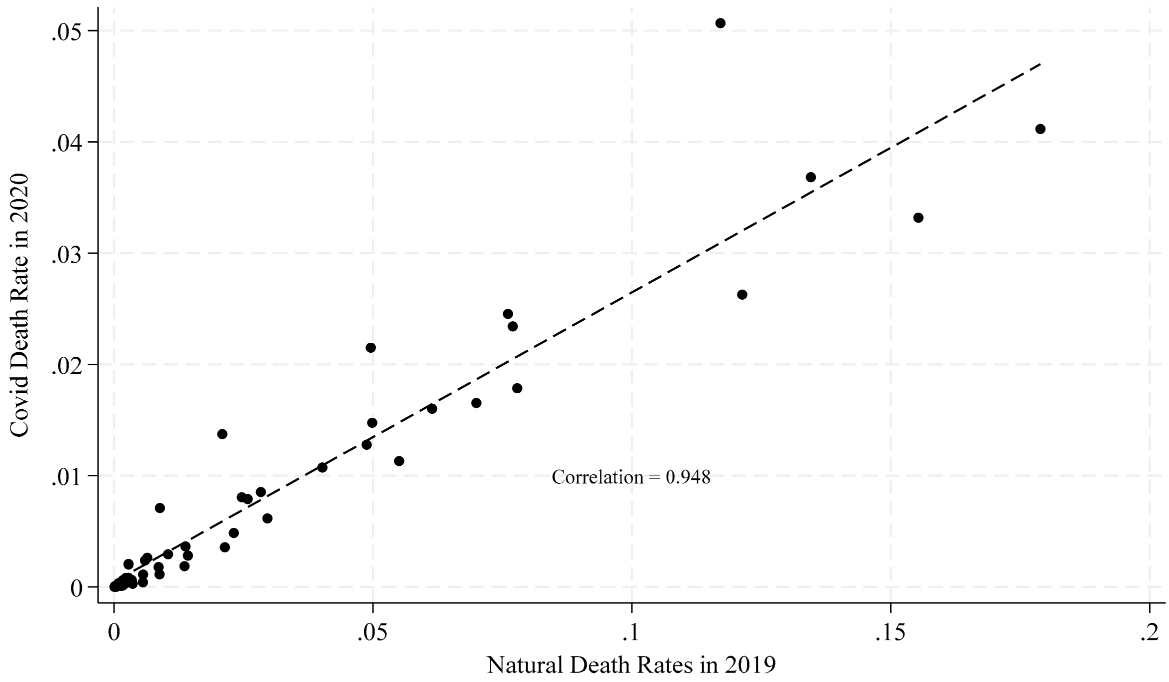

Figure S3. Indiana Natural Deaths in 2019 vs. Non-COVID Natural Deaths in 2020

Figure shows scatterplot of natural mortality in Indiana over April-December 2019 against Non-Covid natural mortality over April-December 2020, for groups defined by age (18-39, 40-49, 50-59, 60-69, 70-79, 80-89, 90+)*gender*race/ethnicity*area-SES quintile, best-fit regression line, and Pearson correlation coefficient.

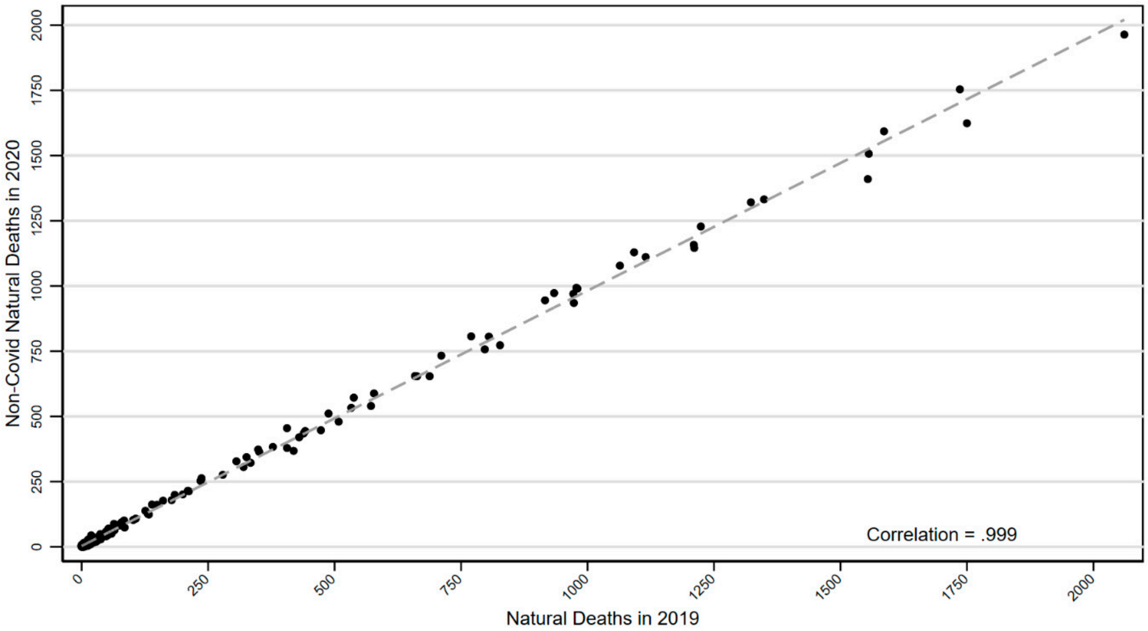

Figure S4. Actual versus Predicted NC-NMR During Pandemic Period

Panel A. Figure shows monthly data for all natural deaths (including COVID-19 deaths) and natural non-COVID deaths, for Indiana for January 2017–December 2022. For the pandemic period starting March 2020, we show both actual and predicted natural non-COVID deaths. Predicted deaths during the pandemic period are based on mean deaths for the same calendar month during 2017–2019 to the same calendar month during the pandemic period. Natural deaths (including COVID-19 deaths) are shown as solid red line; this shows two large COVID-related peaks in late 2020 and late 2021–early 2022. Natural non-COVID deaths (all natural deaths minus COVID-19 deaths) are shown as solid blue line. Predicted natural non-COVID deaths are shown as dashed green line. **Panel B.** Figure is similar to Panel A, but: (i) is limited to pandemic period; (ii) drops line for all natural deaths; and (iii) adds dotted lines showing upper and lower 95% CIs around predicted non-COVID natural deaths.

Panel A. Actual versus Predicted Non-COVID Natural Mortality

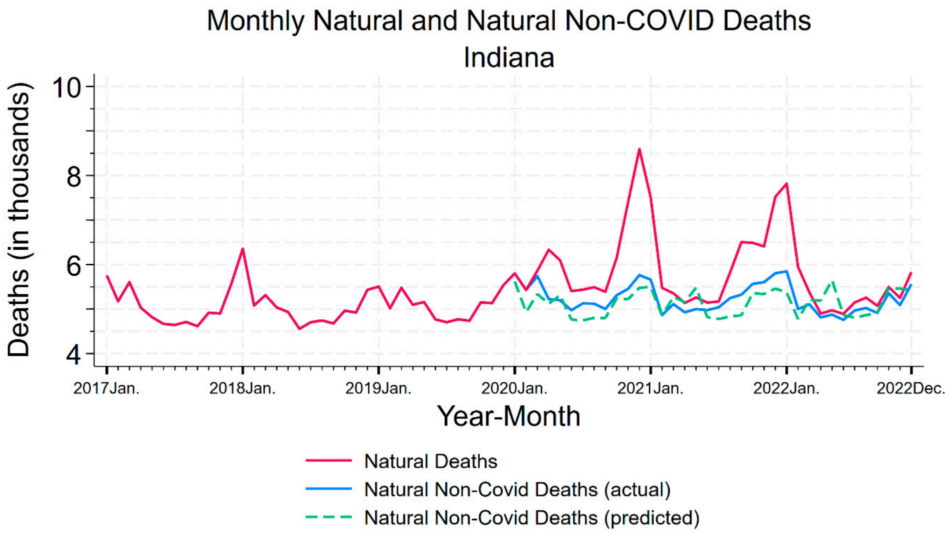

Panel B. Measured and Predicted Non-COVID Natural Deaths, with Confidence Intervals

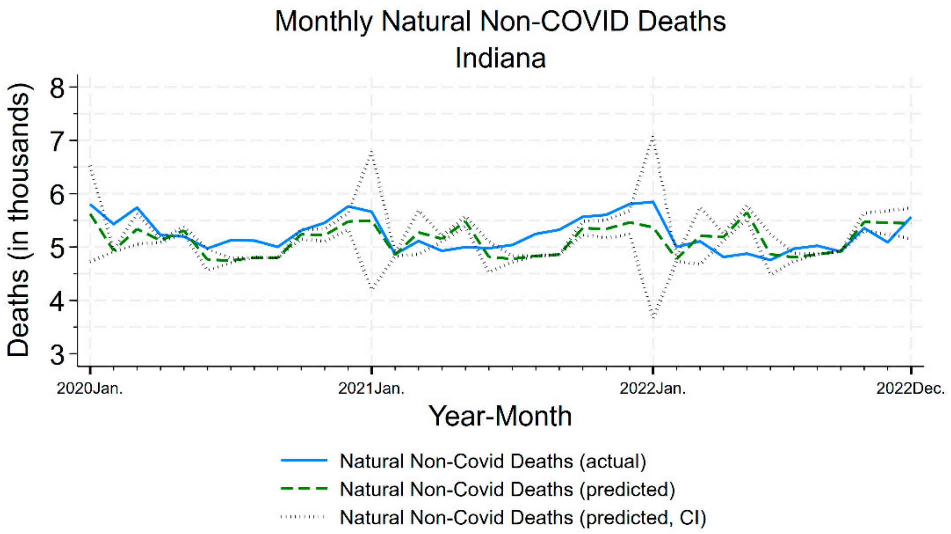

Figure S5. Raw vs. Interpolated Deaths for Vaccinated Persons

Figure shows raw and interpolated counts of natural deaths of vaccinated decedents (1+ doses) in IHIE linked vaccination and mortality records, for ages 15-59 (**Panel A**), and ages 60+ (**Panel B**), from all natural causes (COVID and non-COVID), excluding immunocompromised persons. **Both panels**. Dashed line shows interpolated number of deaths for August-October 2021. Counts of vaccinated decedents are lagged by 30 days relative to the date of the first vaccine dose.

Panel A. Ages 15-59

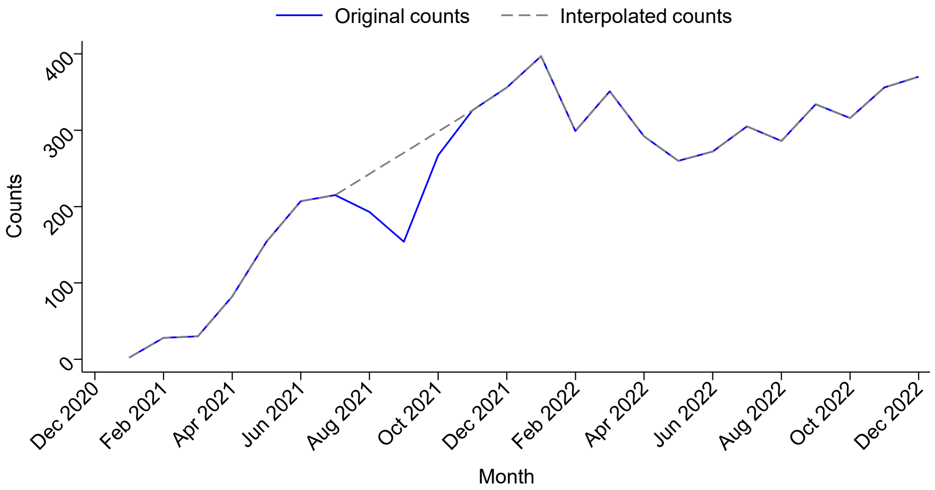

Panel B. Ages 60+

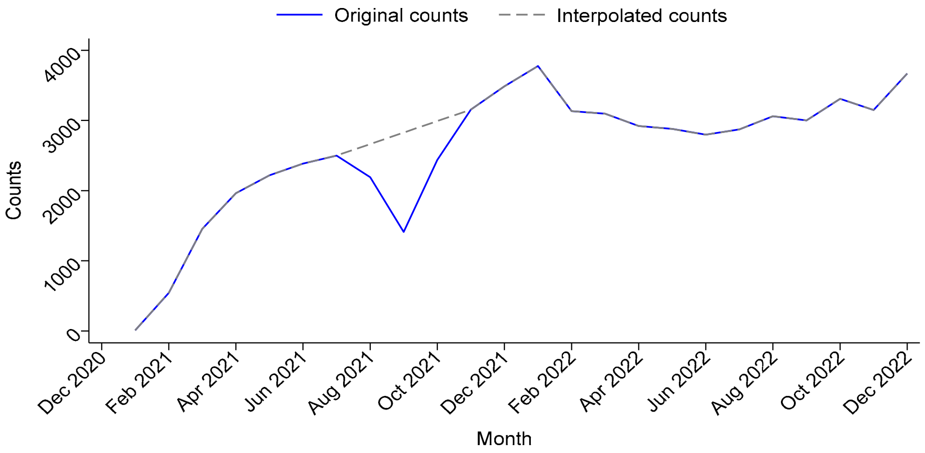

Figure S6. Vaccination Rates for Adults by Age Group in Indiana

Vaccination rates over time for Indiana residents by age range, as a fraction of 2020 population. Age is measured at time of first dose

Panel A. Full Vaccination Rates

Full vaccination rates. defined as at least 1 J&J dose, or 2 mRNA doses (Pfizer, Moderna, or mixed), as percentage of population.

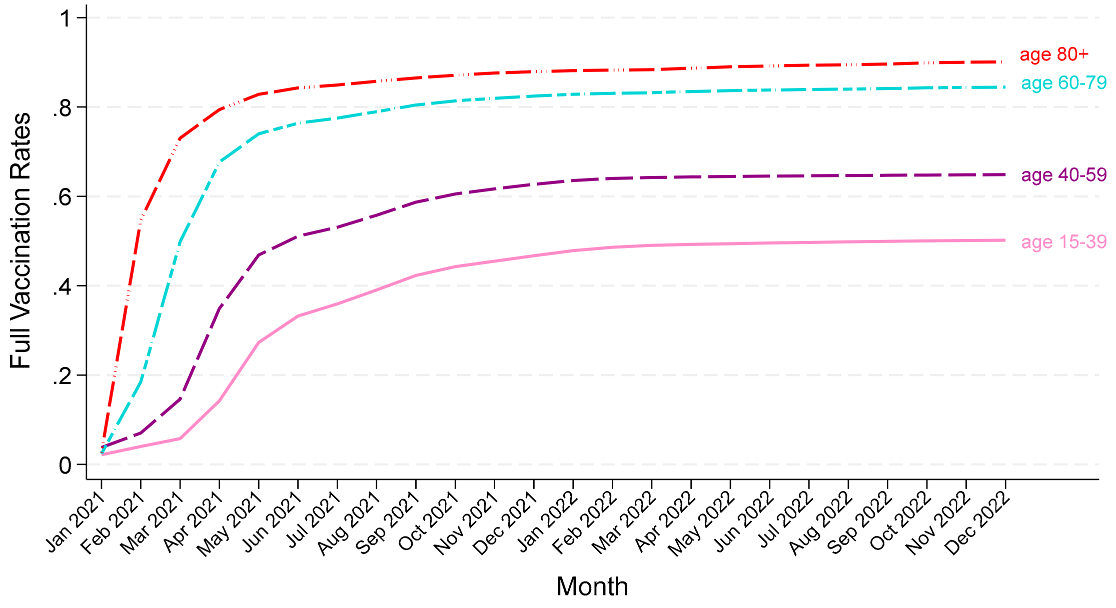

Panel B. At least Three Dose Vaccination Rates

Booster rates as percentage of people receiving full vaccination.

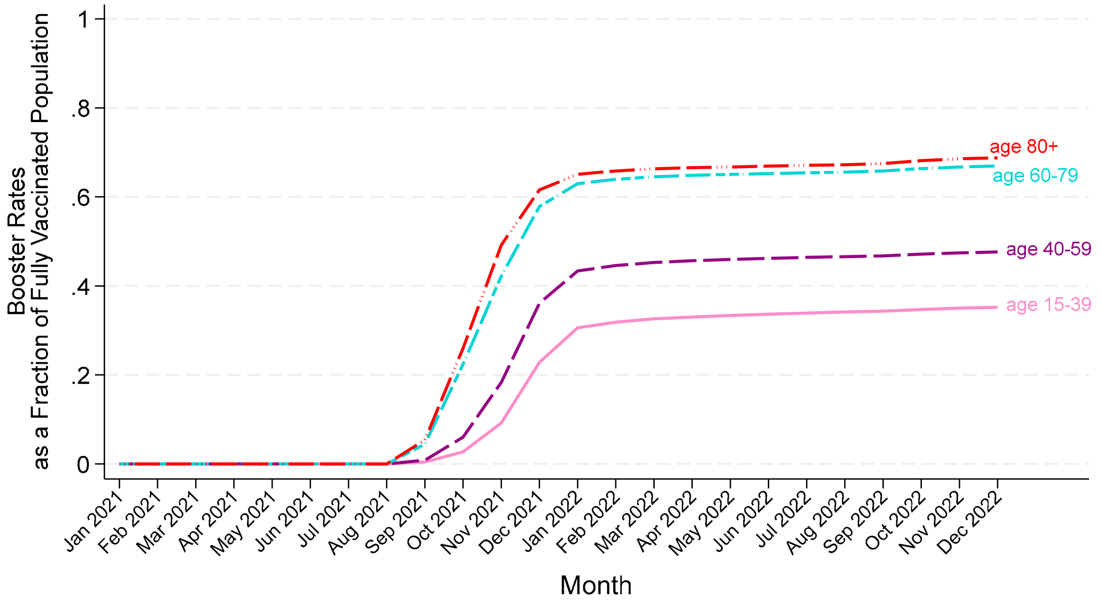

Figure S7. RMR and HVB for ages 60+ with any number of doses

Figure provides information similar to text Figure 1 (for HVBCF) and text Figure 2 (for RMR) for vaccinees with any number of vaccine doses. In contrast, text shows separate results for primary vaccination (1-2 doses) and booster vaccination (3+ doses), by calendar quarter over 1Q2021-4Q2022, for persons aged 60+. **Panel A**. HVBCF. **Panel B**. RMR for Pfizer and Moderna (left-hand graph) and Pfizer/Moderna RMR ratio (right-hand graph). **Both panels**. Estimates for 3Q-4Q2021 are adjusted for IHIE undercount of vaccinated decedents during August-October 2021. Sample excludes immunocompromised persons. Vertical lines show 95% confidence intervals. Vertical dashed lines show start and end of vaccine-available, high-mortality period (2Q2021-1Q2022).

Panel A. HVBCF

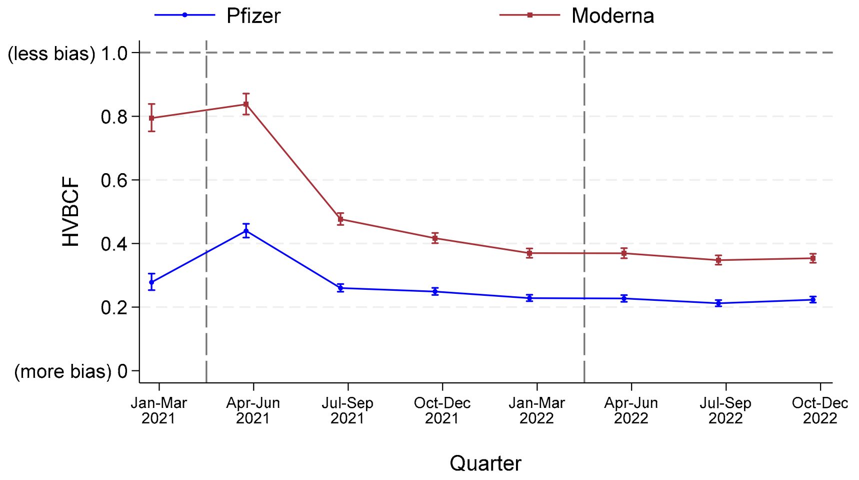

Panel B. RMR and Pfizer/Moderna RMR Ratio

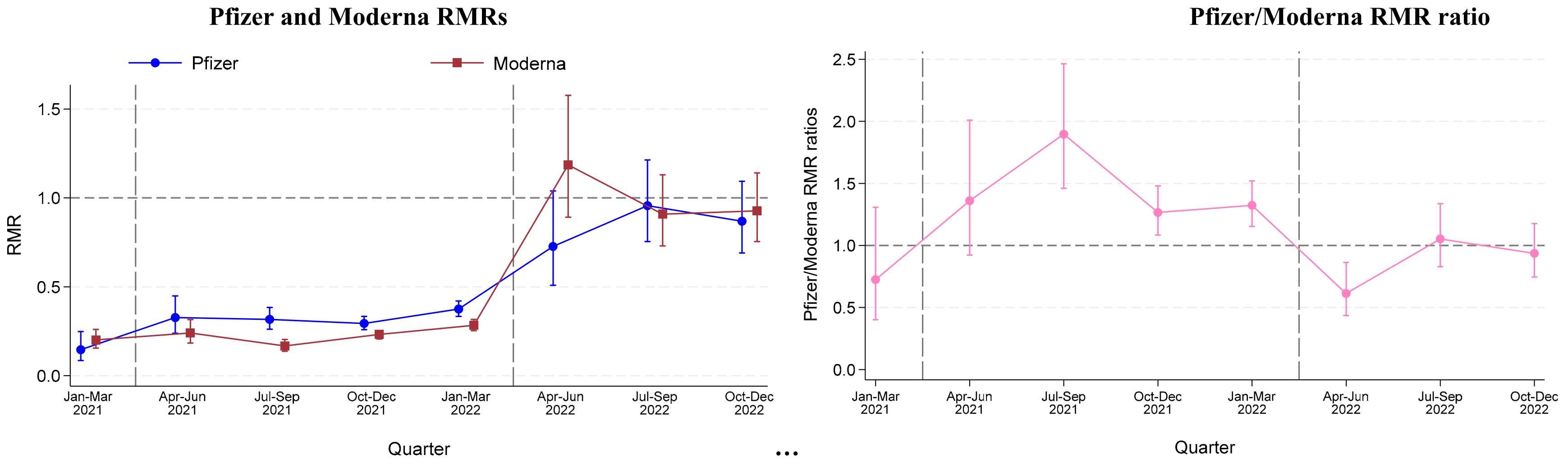

Figure S8. Healthy Vaccinee Bias Correction Factor for Ages 15-59

Figure shows the healthy vaccinee bias correction factor (HVBCF) by calendar quarter over 1Q2021-4Q2022, for Pfizer and Moderna vaccines aged 15-59, with any number of vaccine doses. **Panel A**. Separate estimated for primary vaccination (left-hand graph) and booster vaccination (right-hand graph). **Panel B**. Combined results for any number of vaccine doses. **Both panels.** Estimates for 3Q-4Q2021 are adjusted for IHIE undercount of vaccinated decedents during August-October 2021. See Supplementary Table S4 for underlying data. Sample excludes immunocompromised persons. Short vertical lines show 95% confidence intervals. Vertical dashed lines show start and end of vaccine-available, high-mortality period (2Q2021-1Q2022).

Panel A. Separate Results for Primary and Booster Vaccination

1 or 2 doses

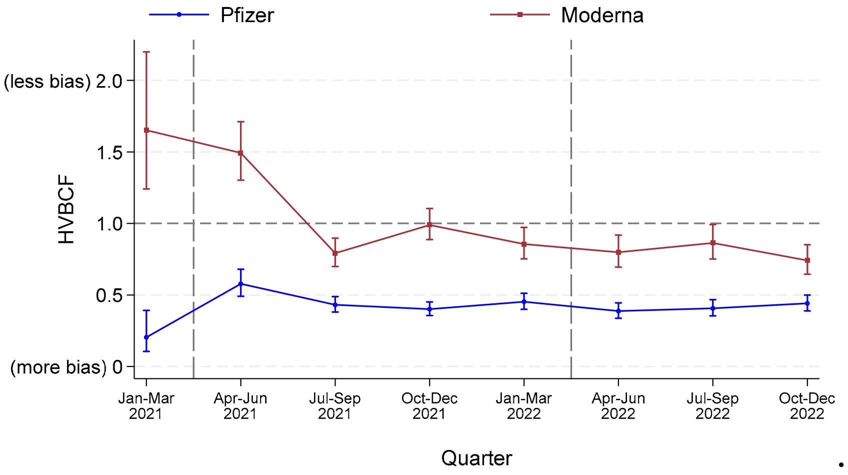

≥3 doses

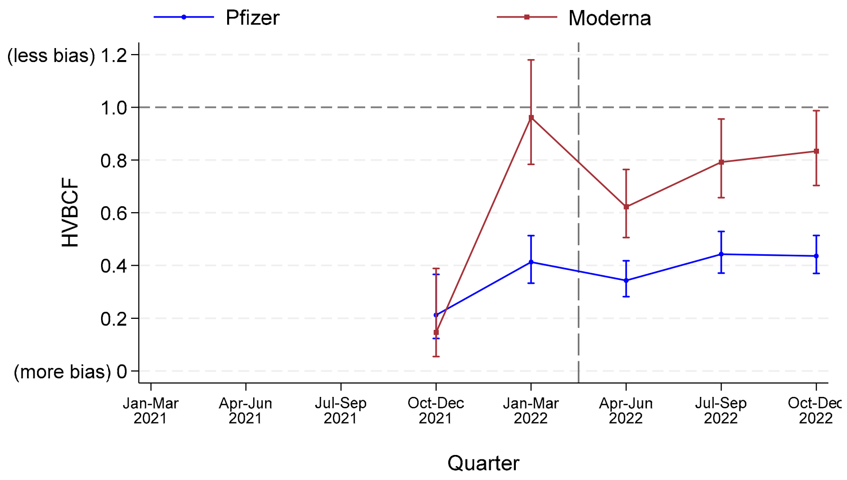

…

Panel B. Any number of doses

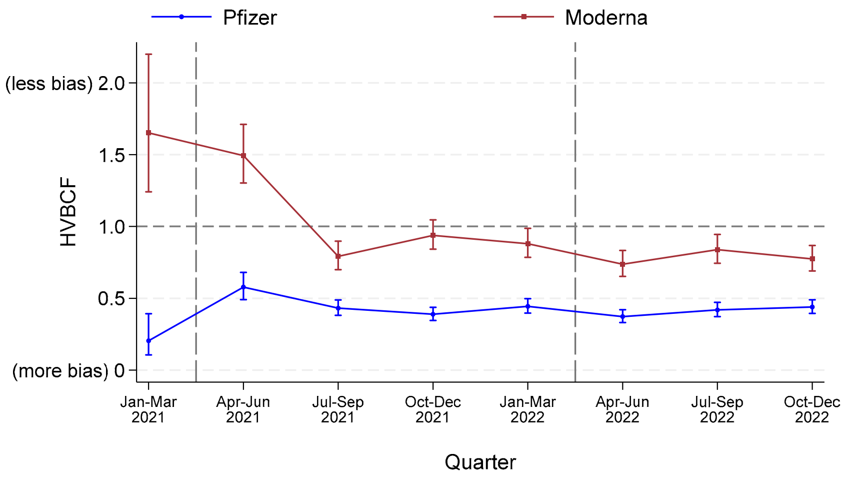

Figure S9. Remaining Mortality Risk for Pfizer and Moderna, ages 15-59

Figure shows remaining mortality risk (RMR), based on CEMP, by calendar quarter over 1Q2021-4Q2022 for Pfizer and Moderna vaccinees aged 15-59. **Panel A**. Shows RMR separately for Pfizer and Moderna vaccinees with primary vaccination (1-2 doses) (left-hand graph) or booster (3+ doses) (right-hand graph). **Panel B**. Shows Pfizer/Moderna ratio of RMRs. **Both panels**. Estimates for 3Q-4Q2021 are adjusted for IHIE undercount of vaccinated decedents during August-October 2021. Sample excludes immunocompromised persons. Vertical lines show 95% confidence intervals. Vertical dashed lines show start and end of vaccine-available, high-mortality period (2Q2021-1Q2022).

Panel A. Separate Pfizer and Moderna RMRs

1 or 2 doses

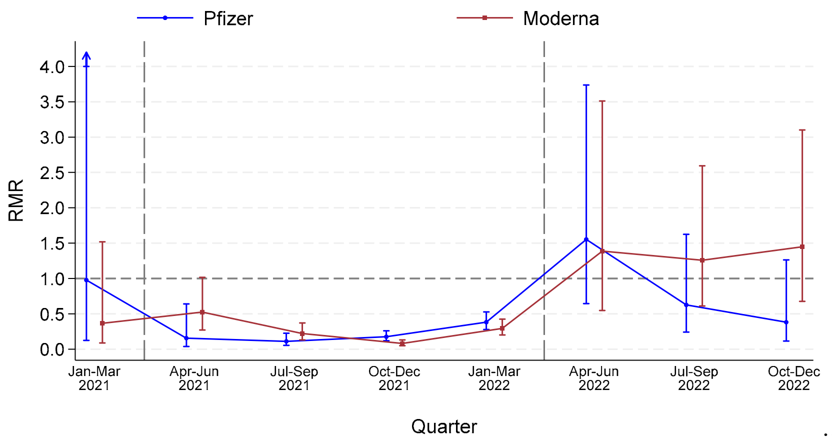

≥3 doses

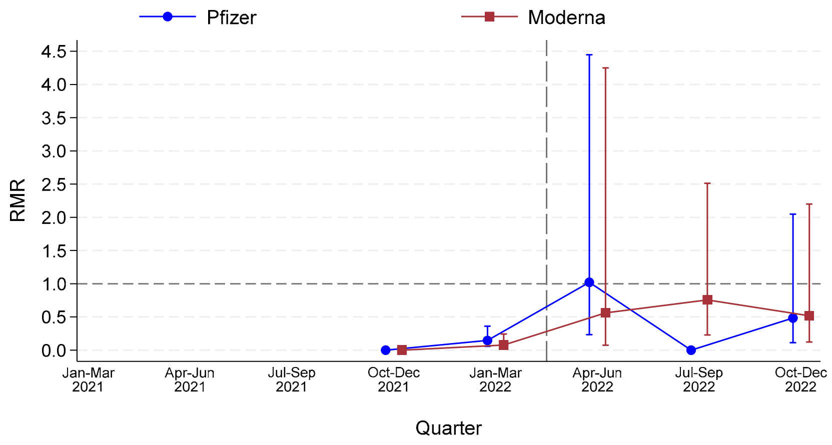

…

Panel B. Pfizer/Moderna RMR Ratio, age 15-59

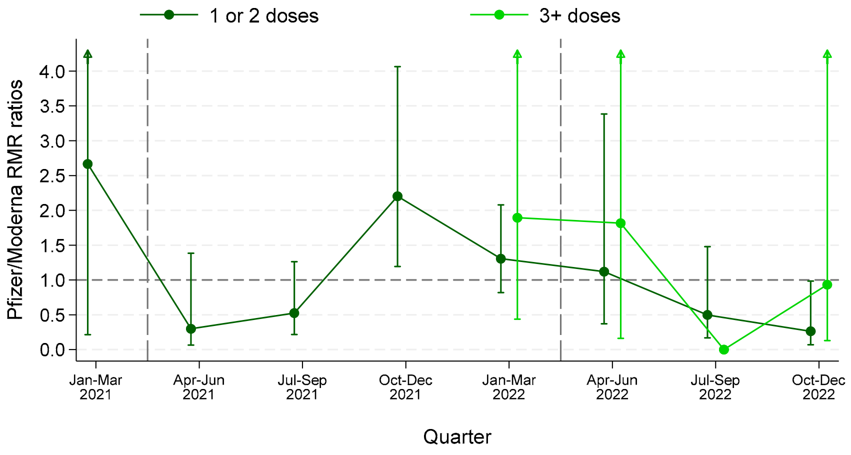

Figure S10. Healthy Vaccinee Bias Correction Factor (Ages 60-79 and 80+)

Figure is similar to text Figure 1 but is for a narrower age range (60-79 and 80+, versus 60+ in the text figure). **Panel A.** Ages 60-79**. Panel B.** 80+. **All Panels.** Figure shows healthy vaccinee bias correction factor (HVBCF) by calendar quarter over 1Q2021-4Q2022, for Pfizer and Moderna vaccinees aged 60-79, with primary vaccination (1-2 doses) (left-hand graph) or booster (3+ doses) (right-hand graph). Estimates for 3Q-4Q2021 are adjusted for IHIE undercount of vaccinated decedents during August-October 2021. See Supplementary Table S4 for underlying data. Sample excludes immunocompromised persons. Short vertical lines show 95% confidence intervals. Vertical dashed lines show start and end of vaccine-available, high-mortality period (2Q2021-1Q2022).

Panel A. Ages 60-79

Primary vaccination (1 or 2 doses)

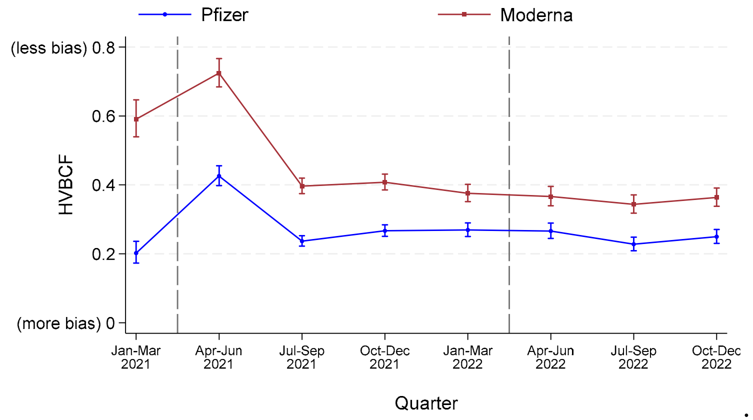

Booster (3+ doses)

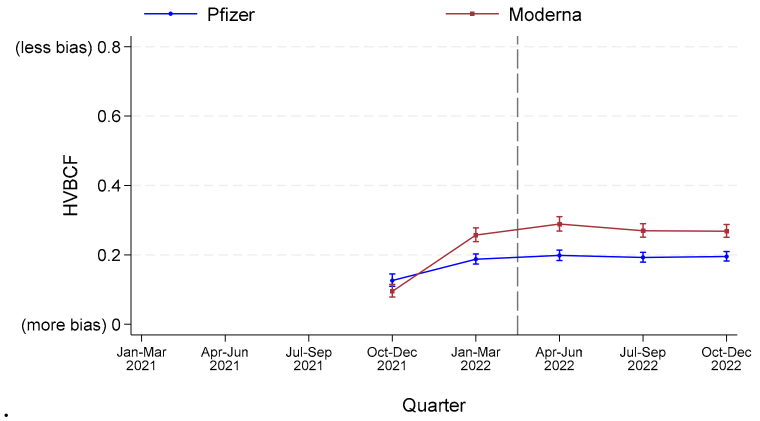

...

Panel B. Ages 80+

Primary vaccination (1 or 2 doses)

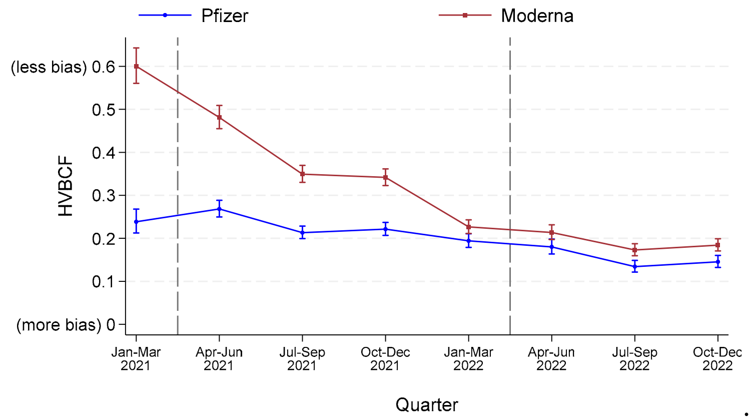

Booster (3+ doses)

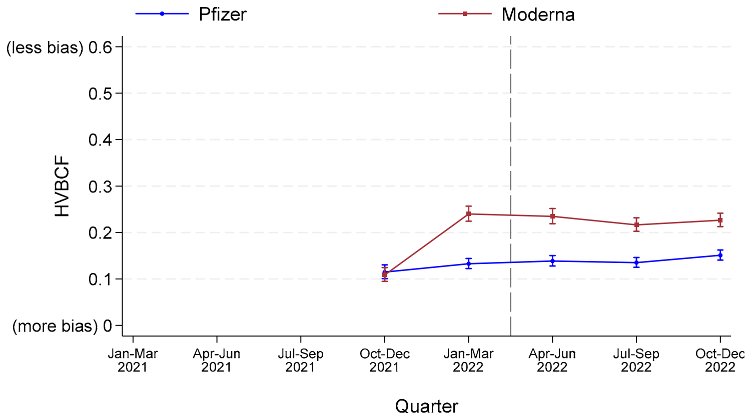

...

Figure S11. Remaining Mortality Risk for Pfizer and Moderna (ages 60-79)

Figure is similar to text Figure 2 but is for a narrower age range (60-79, versus 60+ in the text figure). Figure shows remaining mortality risk (RMR), based on CEMP, by calendar quarter over 1Q2021-4Q2022 for Pfizer and Moderna vaccinees aged 60-79. **Panel A**. Shows RMR separately for Pfizer and Moderna vaccinees with primary vaccination (1-2 doses) (left-hand graph) or booster (3+ doses) (right-hand graph). **Panel B**. Shows Pfizer/Moderna ratio of RMRs. **Both panels**. Estimates for 3Q-4Q2021 are adjusted for IHIE undercount of vaccinated decedents during August-October 2021. Sample excludes immunocompromised persons. Vertical lines show 95% confidence intervals. Vertical dashed lines show start and end of vaccine-available, high-mortality period (2Q2021-1Q2022).

Panel A. Separate Pfizer and Moderna RMRs

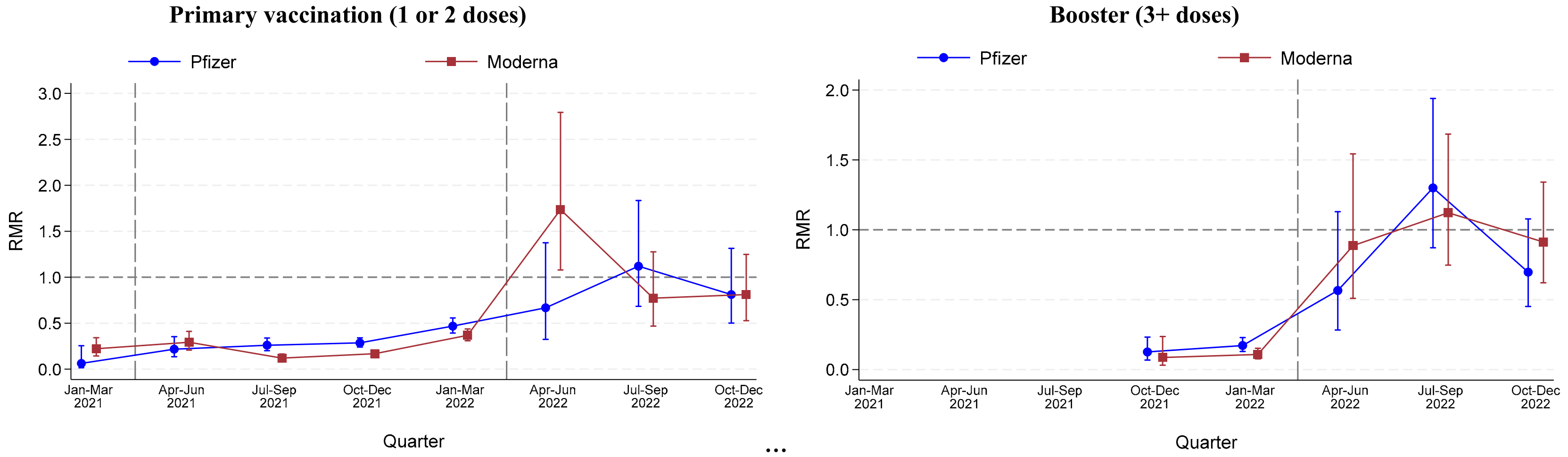

Panel B. Pfizer/Moderna RMR Ratios

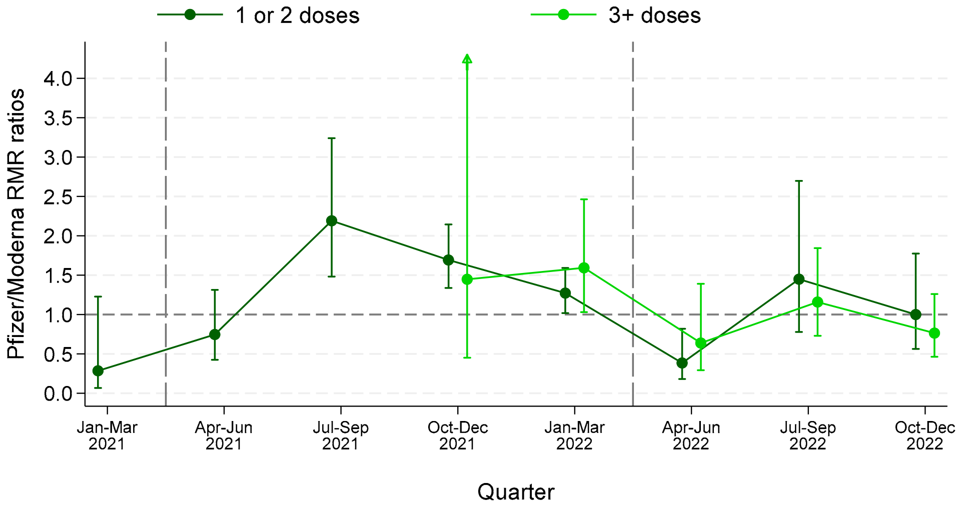

Figure S12. Remaining Mortality Risk for Pfizer and Moderna (ages 80+)

Figure is similar to text Figure 2 but is for a narrower age range (80+, versus 60+ in the text figure). Figure shows remaining mortality risk (RMR), based on CEMP, by calendar quarter over 1Q2021-4Q2022 for Pfizer and Moderna vaccinees aged 80+. **Panel A**. Shows RMR separately for Pfizer and Moderna vaccinees with primary vaccination (1-2 doses) (left-hand graph) or booster (3+ doses) (right-hand graph). **Panel B**. Shows Pfizer/Moderna ratio of RMRs. **Both panels**. Estimates for 3Q-4Q2021 are adjusted for IHIE undercount of vaccinated decedents during August-October 2021. Sample excludes immunocompromised persons. Vertical lines show 95% confidence intervals. Vertical dashed lines show start and end of vaccine-available, high-mortality period (2Q2021-1Q2022).

Panel A. Separate Pfizer and Moderna RMRs

Primary vaccination (1 or 2 doses) **Booster (3+ doses)**

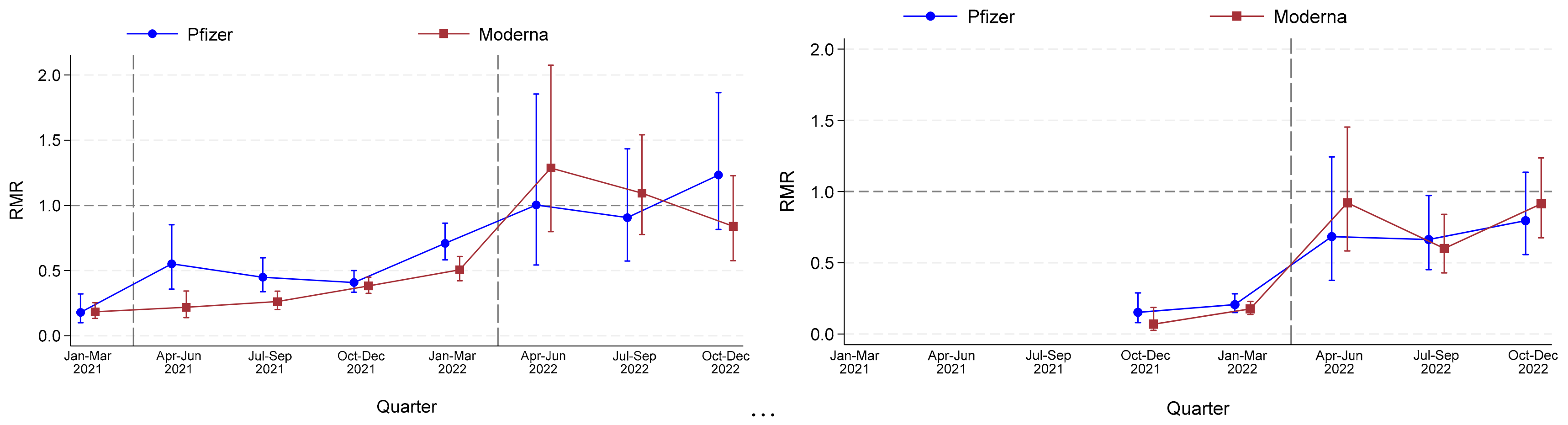

...

Panel B. Pfizer/Moderna RMR Ratios

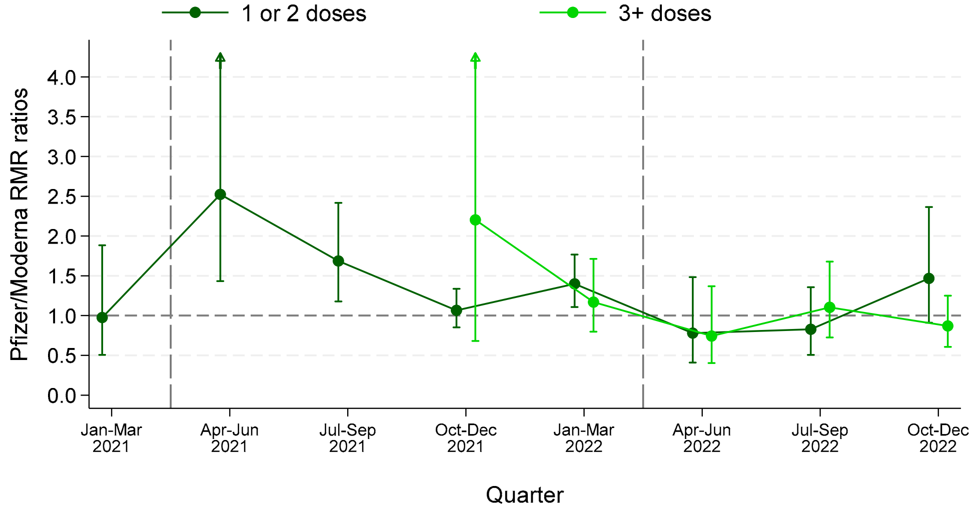

Figure S13. Healthy Vaccinee Bias Correction Factor (Ages 60+), by sex

Figure shows the healthy vaccinee bias correction factor (HVB_CF) by calendar quarter over 1Q2021-4Q2022, for Pfizer and Moderna vaccinees aged 60+, with primary vaccination (1-2 doses) (left-hand graph) or booster (3+ doses) (right-hand graph). HVB_CF is ratio of non-COVID NMR for vaccinated (with indicated number of doses)/non-COVID NMR for unvaccinated. Estimates for 3Q-4Q2021 are adjusted for IHIE undercount of vaccinated decedents during August-October 2021. See Supplementary Table S4 for underlying data. Sample excludes immunocompromised persons. Vertical lines show 95% confidence intervals. Vertical dashed lines show start and end of vaccine-available, high-mortality period (2Q2021-1Q2022).

Panel A. Male

Primary vaccination (1 or 2 doses)

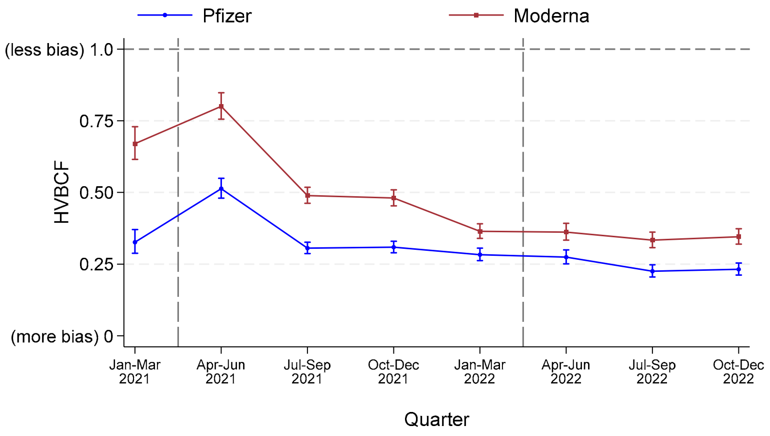

...

Booster (3+ doses)

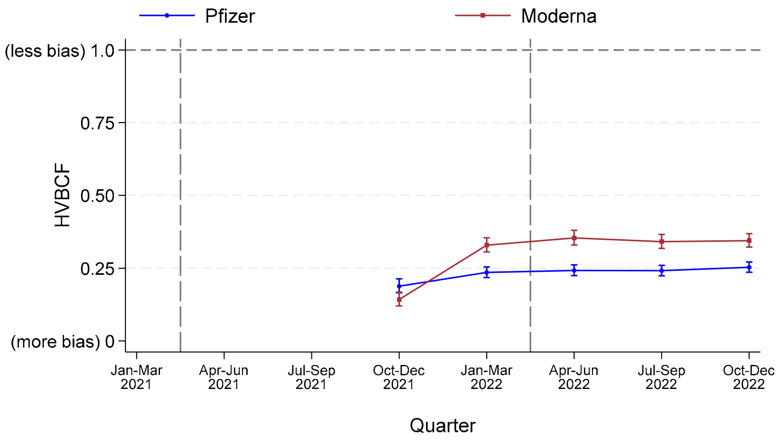

Panel B. Female

Primary vaccination (1 or 2 doses)

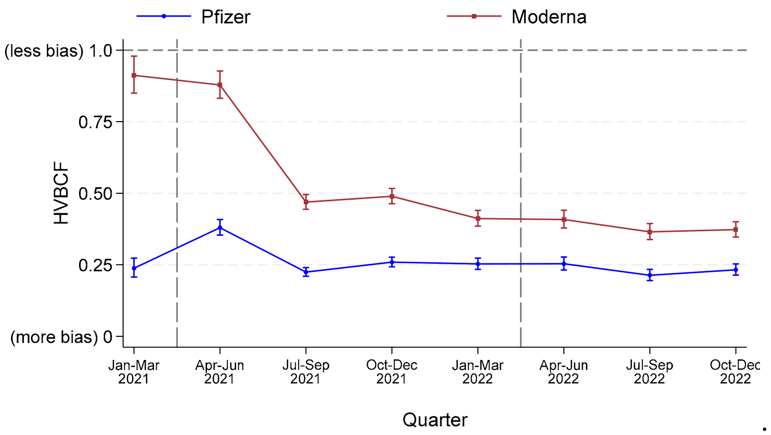

...

Booster (3+ doses)

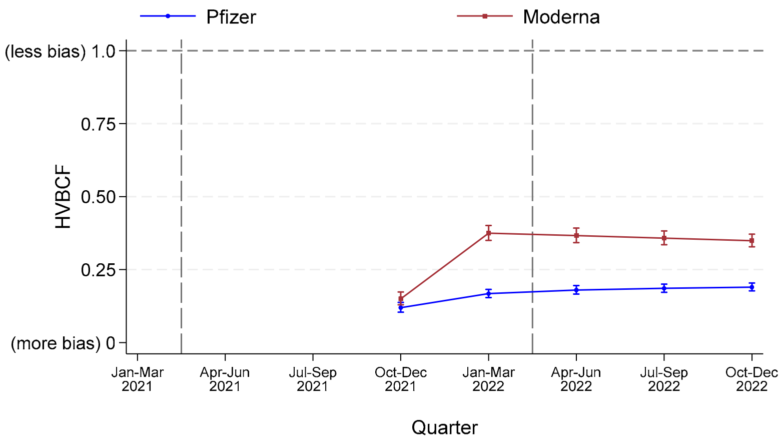

Figure S14. Remaining Mortality Risk for Pfizer and Moderna, ages 60+ by sex

Figure shows remaining mortality risk (RMR), based on CEMP, by calendar quarter over 1Q2021-4Q2022for Pfizer and Moderna vaccinees aged 60+ with primary vaccination (1-2 doses) (left-hand graph) or booster (3+ doses) (right-hand graph). **Panel A**. Shows RMR separately for Pfizer and Moderna vaccinees. **Panel B**. Shows Pfizer/Moderna ratio of RMRs. **Both panels**. Estimates for 3Q-4Q2021 are adjusted for IHIE undercount of vaccinated decedents during August-October 2021. Sample excludes immunocompromised persons. Vertical lines show 95% confidence intervals. Vertical dashed lines show start and end of vaccine-available, high-mortality period (2Q2021-1Q2022).

Panel A. Separate Pfizer and Moderna RMRs

Male

Primary vaccination (1 or 2 doses)

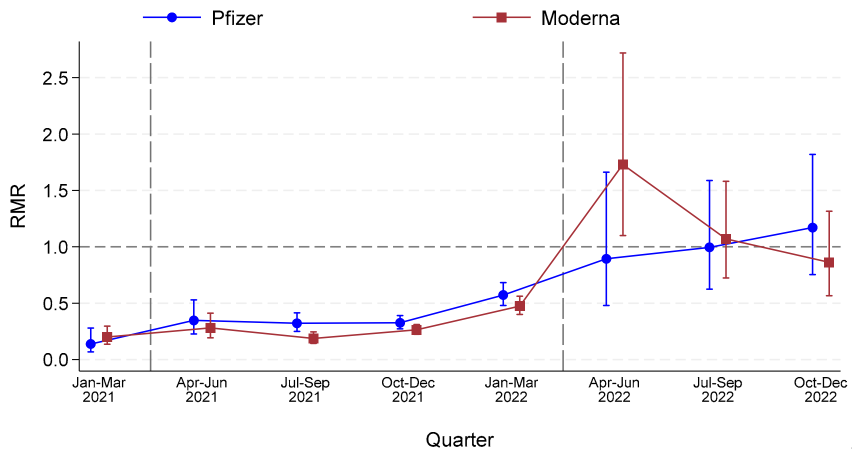

Booster (3+ doses)

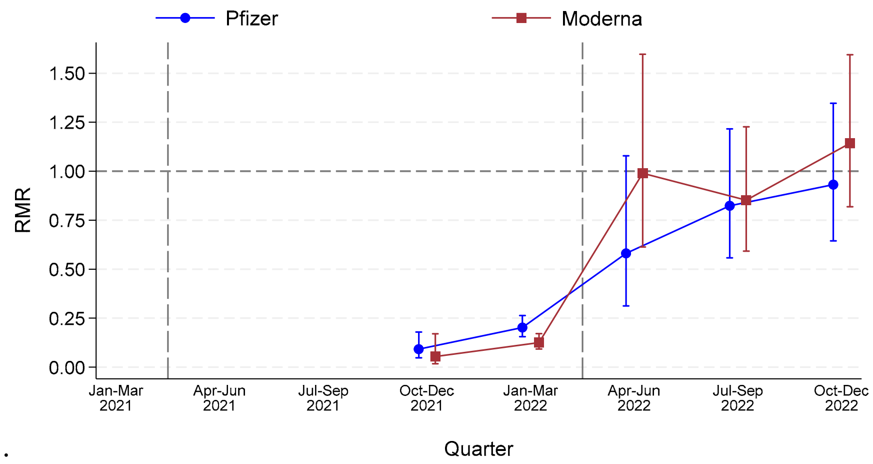

…

Female

Primary vaccination (1 or 2 doses)

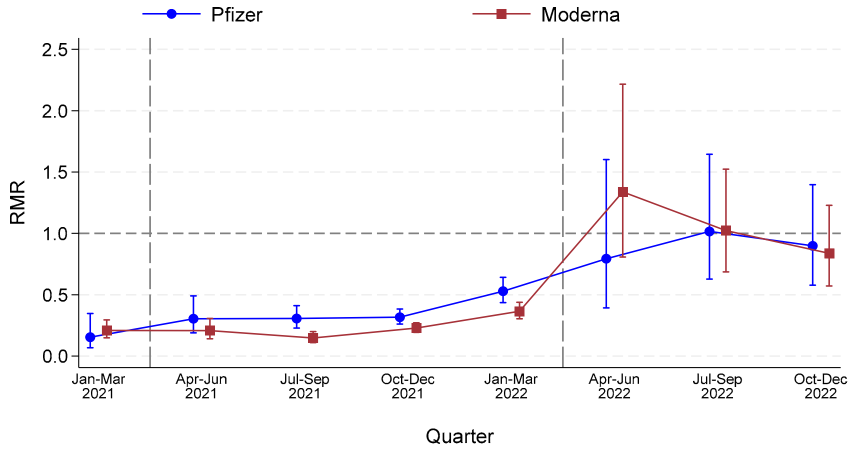

Booster (3+ doses)

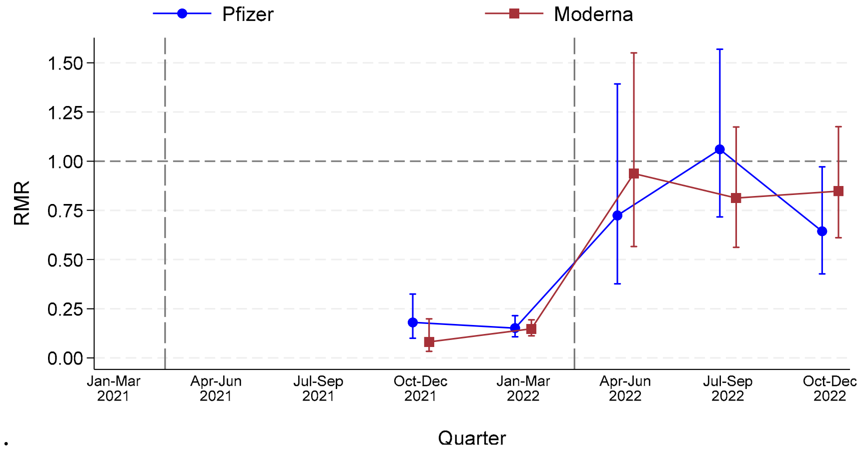

…

Panel B. Pfizer/Moderna RMR Ratios

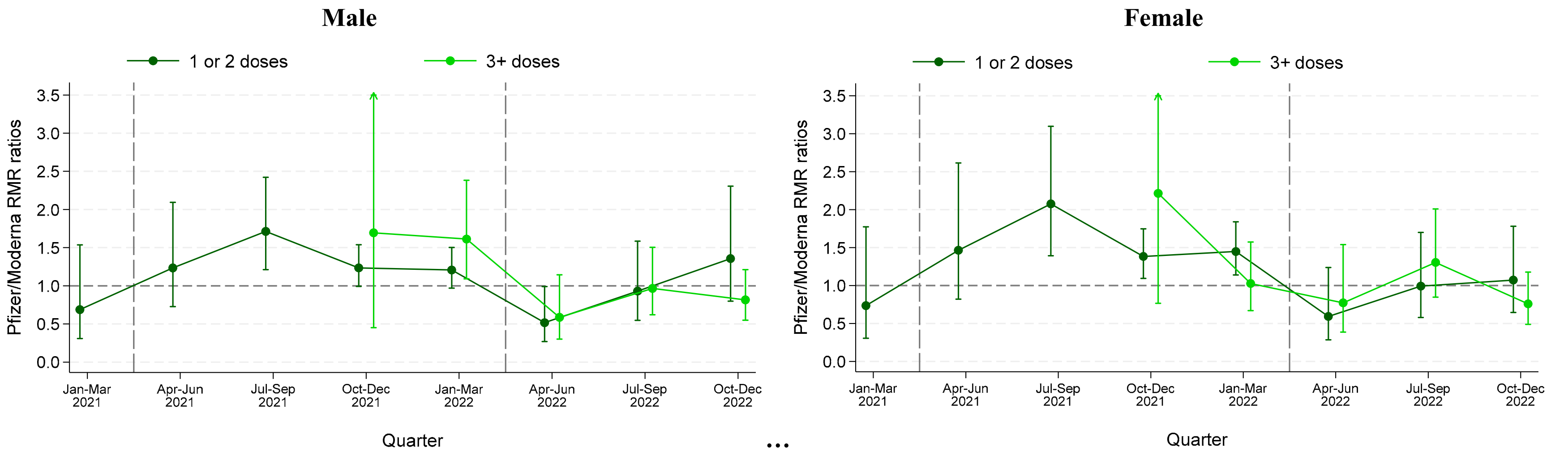

Figure S15. Monthly Vaccination Counts by Manufacturer

Figure shows monthly vaccination counts separately for Pfizer and Moderna, using original vaccination dates (not shifted forward 30 days), separately for ages 60+ (left panels) and 15-59 (right panels). **Panel A**. First dose. **Panel B**. Second dose. **Panel C**. Third dose.

Panel A. First Dose

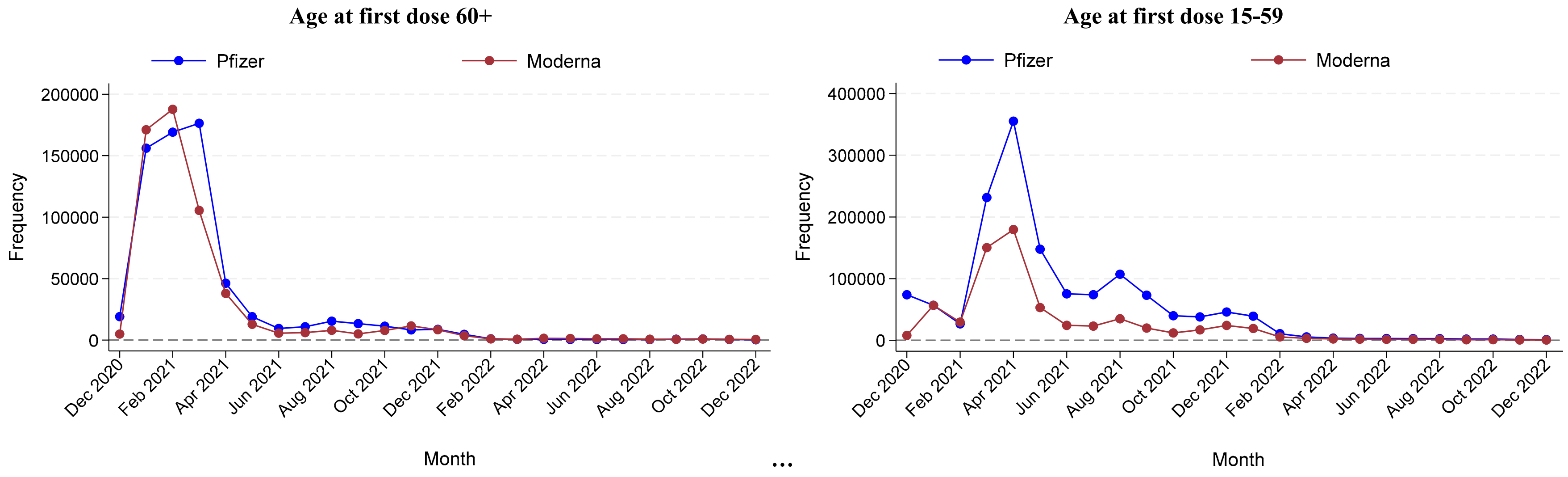

...

Panel B. Second Dose

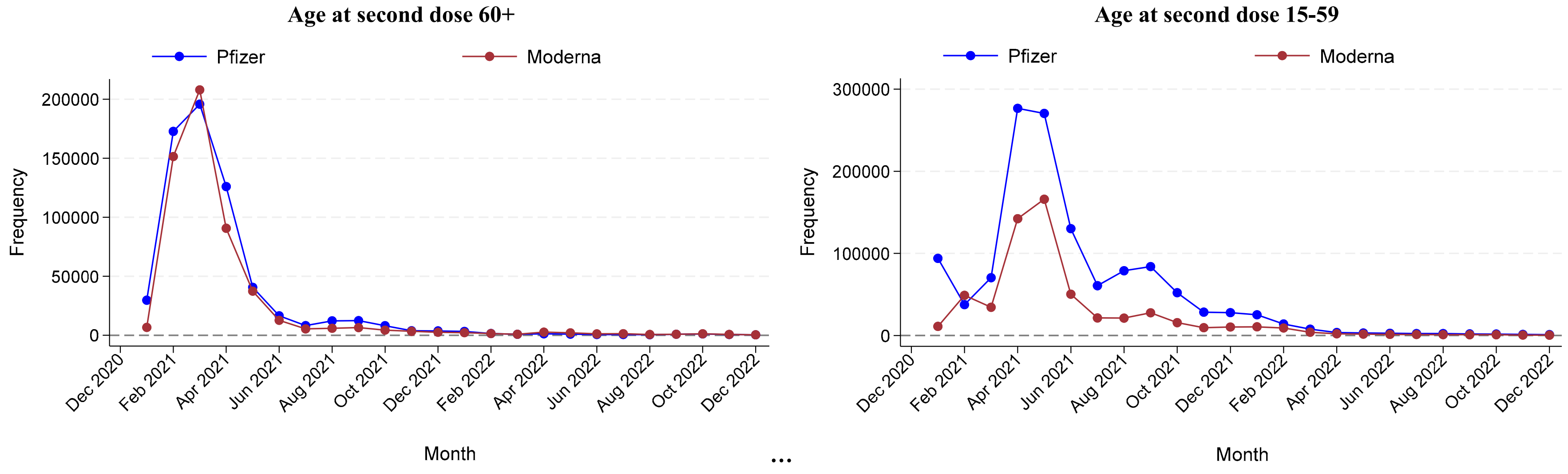

...

Panel C. Third Dose

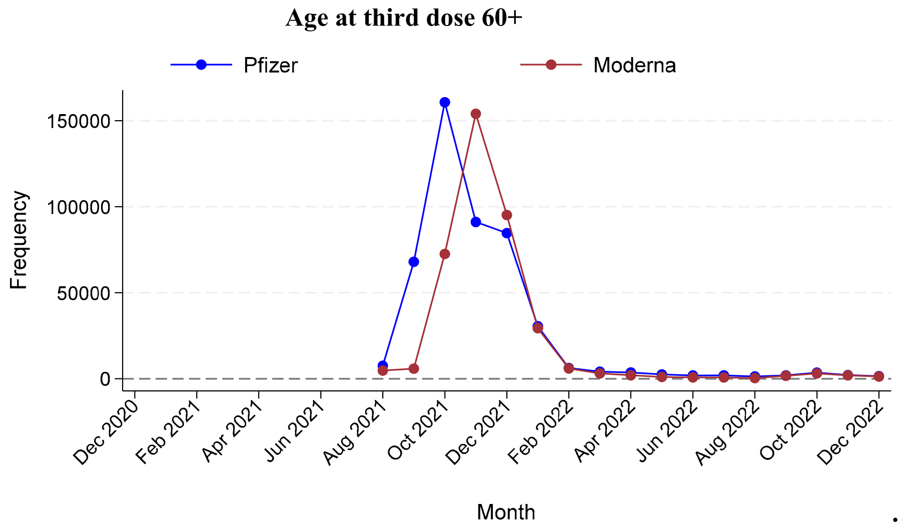

...

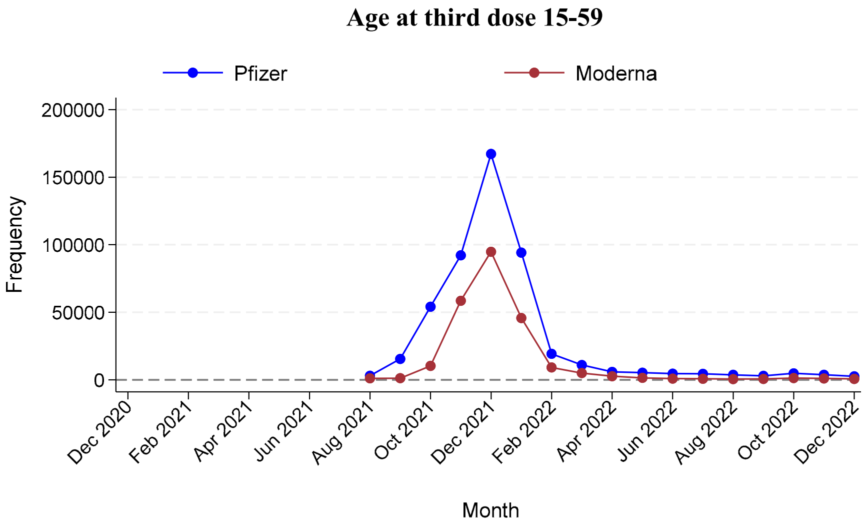

Figure S16. RMR Pfizer/Moderna ratios, univariate vs. multivariate regressions

Figure relies on data in Table S8. Figure compares Pfizer/Moderna RMR ratios based on CEMP (same as text Figure 2, Panel B for ages 60+, and Figure 3, Panel B for ages 15-59), with Pfizer/Moderna RMR ratios from multivariate logistic regressions, by calendar quarter and number of doses. **Panel A**. Ages 60+. Left panel is for primary vaccination (1 or 2 doses). Right panel is for booster recipients (3 or 4 doses). **Panel B**. Ages 15–59. Figure shows results for any number of doses. The multivariate regressions are estimated among natural-cause decedents and model the odds of COVID-19 death relative to non-COVID natural death, adjusting for a quadratic in age, sex, race, education, marital status, and quintiles of the Graham Social Deprivation Index. See Table S8 for details. Vertical bars show 95% confidence intervals. Ratios above 1.0 indicate higher RMR for Pfizer than Moderna. Dashed vertical lines indicate the core period, 2Q2021–1Q2022.

Panel A. Age 60+

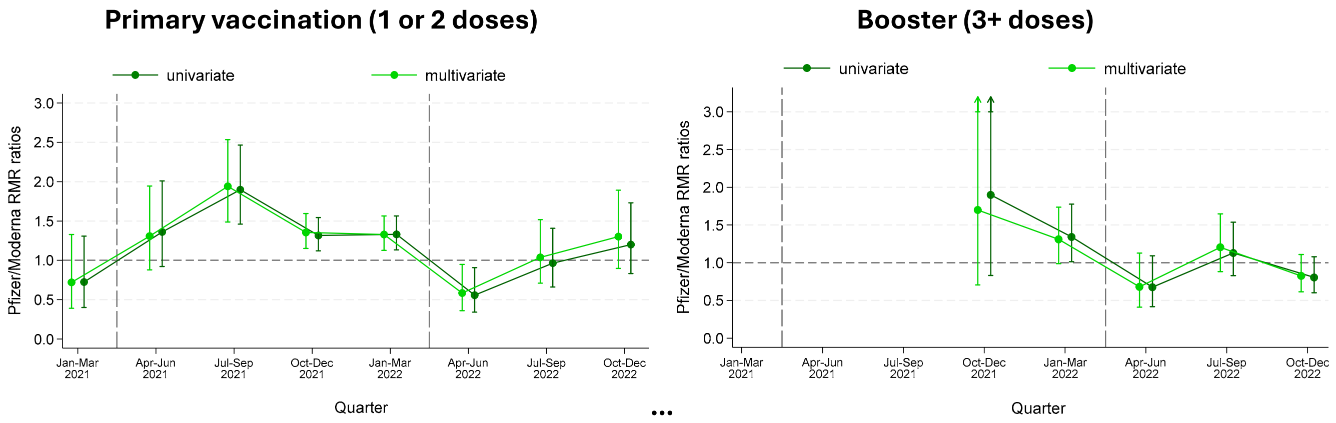

Panel B. Age 15-59

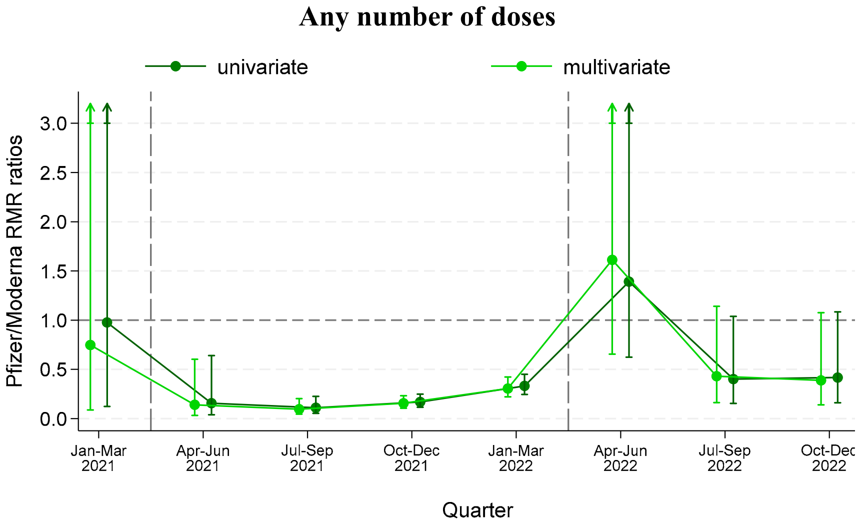

Figure S17. Mortality Relative to Vaccination Date for 1Q2021 Vaccinees

Figure shows mortality rates and CEMP, separately for Pfizer and Moderna vaccinees, for persons who received their first Pfizer or Moderna dose in 1Q2021. **Panel A**. Ages 60+. Mortality rates measured by week since first dose, beginning with week 5. **Panel B**. Ages 15-59. In Panel B, rates are shown by month since first dose (instead of week) because death counts are sparse. Month 1 is days 1-30, month 2 is days 31-60, etc. **Both Panels**. Left-hand graph is COVID-19 mortality rate (%). Middle graph is non-COVID-19 natural mortality rate (NCNMR) (%). Right-hand graph shows CEMP. Numerator for COVID-19 mortality rate and NCNMR is number of deaths among persons receiving the indicated vaccine type. Denominator is number of persons who received a first dose of indicated type in 2021Q1.

Panel A. Ages 60+, any number of doses

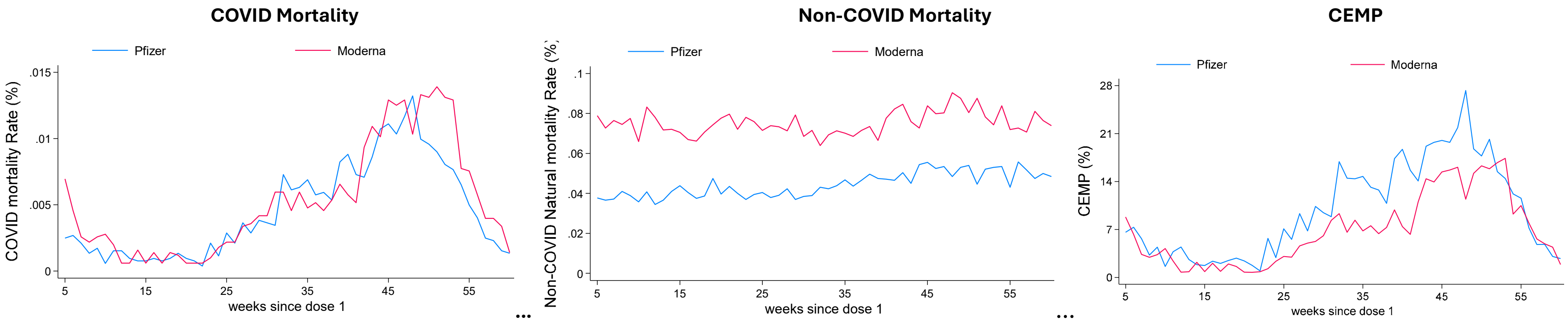

Panel B. Ages 15-59, any number of doses

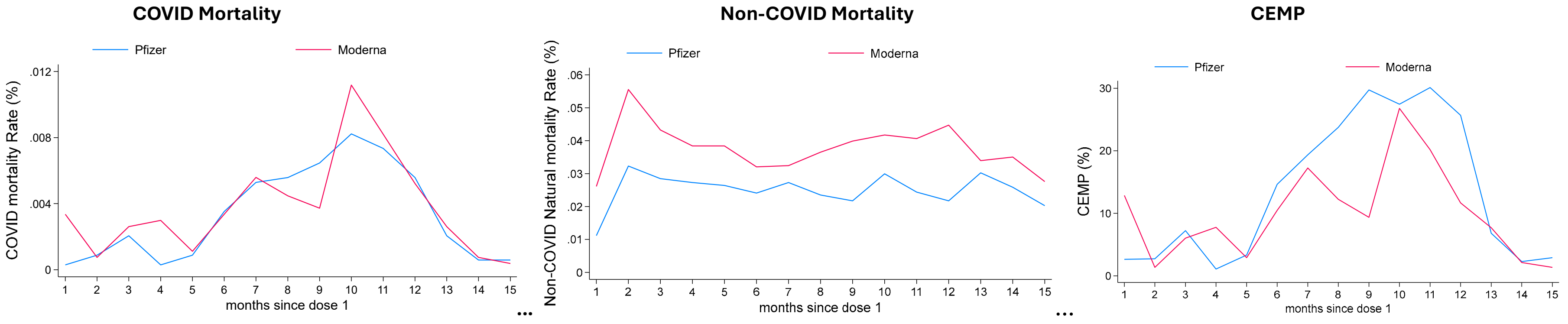

Figure S18. Sensitivity: Booster-Dose RMR for Age 60+ with Mixed Booster Dose

Figure compares booster-dose RMR estimates among persons aged 60+, allowing for mixed booster-versus-primary vaccine type, to estimates which consider only persons who received a single vaccine type. **Panel A. RMR Estimates**. The left panel compares RMR for persons three Pfizer doses (same as text Figure 2, Panel A), to RMR for persons receiving primary Pfizer vaccination plus Moderna booster. The right panel compares RMR for persons three Moderna doses (same as text Figure 2, Panel A), to RMR for persons receiving primary Moderna vaccination plus Pfizer booster. Panel B. Pfizer/Moderna RMR ratio, including versus excluding mixed vaccine types. **Both panels**. Vertical bars show 95% confidence intervals. Horizontal dashed line at 1.0 indicates no reduction in mortality risk relative to unvaccinated persons. Vertical dashed lines indicate start and end of core vaccine-available, high-mortality period 2Q2021–1Q2022.

Panel A. RMRs Including Mixed Vaccine Types

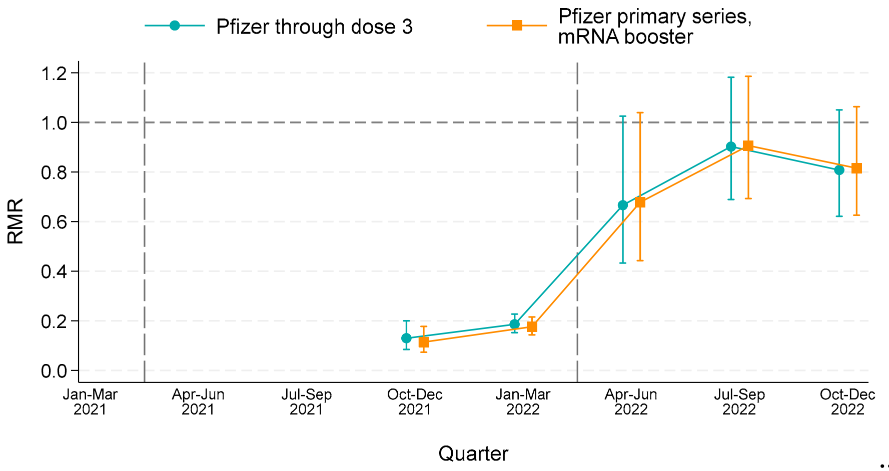

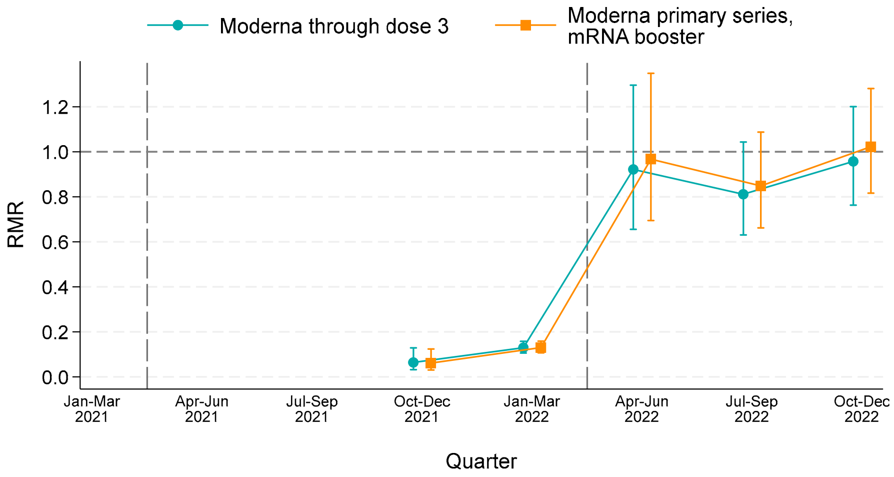

...

Panel B. Pfizer/Moderna Ratio Including Mixed Vaccine Types

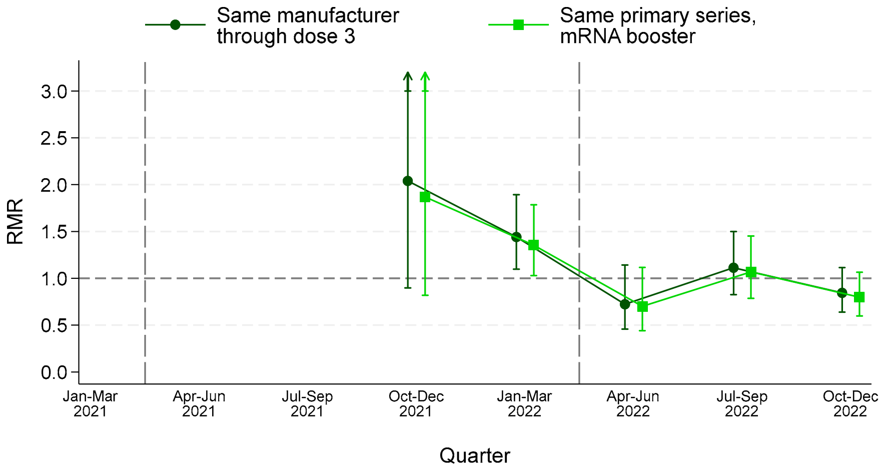

Supplement: Supplementary file 1 [file vaccines-14-00717-s001.zip › vaccines-4494269-supplementary.pdf]
